# Gene expression changes in response to aging compared to heat stress, oxidative stress and ionizing radiation in *Drosophila melanogaster*

**DOI:** 10.18632/aging.100499

**Published:** 2012-11-30

**Authors:** Gary Landis, Jie Shen, John Tower

**Affiliations:** Molecular and Computational Biology Program, Department of Biological Sciences, University of Southern California, Los Angeles, CA 90089-2910; USA

**Keywords:** Oxidative stress, ROS, mitochondria, GST, Nrf2

## Abstract

Gene expression changes in response to aging, heat stress, hyperoxia, hydrogen peroxide, and ionizing radiation were compared using microarrays. A set of 18 genes were up-regulated across all conditions, indicating a general stress response shared with aging, including the heat shock protein (Hsp) genes *Hsp70, Hsp83 and l(2)efl*, the glutathione-S-transferase gene *GstD2*, and the mitochondrial unfolded protein response (mUPR) gene *ref(2)P*. Selected gene expression changes were confirmed using quantitative PCR, Northern analysis and GstD-GFP reporter constructs. Certain genes were altered in only a subset of the conditions, for example, up-regulation of numerous developmental pathway and signaling genes in response to hydrogen peroxide. While aging shared features with each stress, aging was more similar to the stresses most associated with oxidative stress (hyperoxia, hydrogen peroxide, ionizing radiation) than to heat stress. Aging is associated with down-regulation of numerous mitochondrial genes, including electron-transport-chain (ETC) genes and mitochondrial metabolism genes, and a sub-set of these changes was also observed upon hydrogen peroxide stress and ionizing radiation stress. Aging shared the largest number of gene expression changes with hyperoxia. The extensive down-regulation of mitochondrial and ETC genes during aging is consistent with an aging-associated failure in mitochondrial maintenance, which may underlie the oxidative stress-like and proteotoxic stress-like responses observed during aging.

## INTRODUCTION

Heat shock protein (Hsp) genes are induced in response to stresses that cause protein denaturation, through activation of the heat shock factor (HSF) [1]. Up-regulation of Hsp genes is also observed during normal aging [2]. For example, both *Hsp70* and *Hsp22* are up-regulated during normal Drosophila aging, and this up-regulation requires functional HSF binding sites (Heat Shock Elements, or HSEs) in the promoters of these genes [3-5]. Genome-wide studies of gene expression changes during Drosophila aging have revealed additional features of a stress response, including the up-regulation of additional oxidative stress-response genes, and the dramatic up-regulation of innate immune response genes [6-8]. In addition, Drosophila aging is characterized by a small but across-the-board down-regulation of mitochondrial metabolism and electron transport chain (ETC) genes [6, 8], and this pattern is also observed in aging mammalian tissues [9], and at early adult ages in both Drosophila and *C. elegans* [10], indicating a conservation of aging mechanisms across species. Both innate immune response genes [6] and Hsp genes [11, 12] have been shown to be predictive biomarkers of individual animal life span when the gene promoters are fused to GFP to create transgenic reporters, thereby supporting the significance of the identified gene expression changes. Here normal aging was compared with multiple stressors to provide further insight into common and unique features.

## RESULTS

### Gene expression changes common to each stress and to aging

Micro-array analysis was used to identify genes whose expression was altered in response to normal aging, hyperoxia, hydrogen peroxide, ionizing radiation and heat stress. A core set of 18 stress-response genes were up-regulated ≥1.5-fold in response to each of the tested stresses as well as during normal aging (Table 1).

**Table 1:** Gene expression changes common to aging and each stress

**Table:** 

CG6489	Hsp70	Heat-shock-protein-70
CG3705	aay	astray
CG32130	stv	starvin
CG4533	l(2)efl	lethal (2) essential for life
CG33229	CG33229	
CG4181	GstD2	Glutathione S transferase D2
CG3821	Aats-asp	Aspartyl-tRNA synthetase
CG5966	CG5966	
CG11030	CG11030	
CG14245	CG14245	
CG14246	CG14246	
CG15784	CG15784	
CG31638	CG31638	
CG13941	Arc2	Arc2
CG1242	Hsp83	Heat shock protein 83
CG10360	ref(2)P	refractory to sigma P
CG32103	CG32103	
CG17725	Pepck	Phosphoenolpyruvate carboxykinase

**Table:** 

GO:0035079	polytene chromosome puffing(5)	5.65E-10
GO:0035080	heat shock-mediated polytene chromosome puffing(5)	5.65E-10
GO:0009408	response to heat(7)	8.87E-08
GO:0009266	response to temperature stimulus(7)	5.70E-07
GO:0034605	cellular response to heat(5)	1.12E-06
GO:0001666	response to hypoxia(5)	4.94E-05
GO:0070482	response to oxygen levels(5)	8.15E-05
GO:0009628	response to abiotic stimulus(7)	2.00E-04

**Table:** 

CG number	Symbol	Gene name
CG10026	CG10026	
CG10467	CG10467	
CG14120	CG14120	
CG14661	CG14661	
CG18302	CG18302	
CG18493	CG18493	
CG18585	CG18585	
CG31148	CG31148	
CG3290	CG3290	
CG3734	CG3734	
CG3940	CG3940	
CG5107	CG5107	
CG5150	CG5150	
CG5804	CG5804	
CG6660	CG6660	
CG8093	CG8093	
CG8147	CG8147	
CG9463	CG9463	
CG9466	CG9466	
CG9468	CG9468	
CG9682	CG9682	
CG5137	Cyp312a1	Cyp312a1
CG3360	Cyp313a1	Cyp313a1
CG8579	Jon44E	Jonah 44E
CG11669	Mal-A7	Maltase A7
CG4123	Mipp1	Multiple inositol polyphosphate phosphatase 1
CG6164	Npc2f	Niemann-Pick type C-2f
CG7754	iotaTry	iotaTrypsin
CG12388	kappaTry	kappaTry
CG12350	lambdaTry	lambdaTry
CG16834	lectin-33A	lectin-33A
CG4979	sxe2	sex-specific enzyme 2

**Table:** 

GO:0006013	mannose metabolic process(3)	0.018117

**Figure 1:**
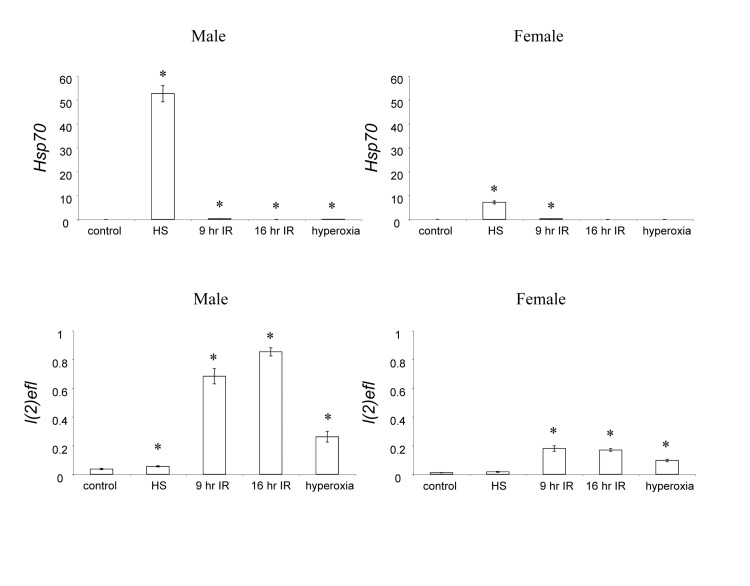
*Hsp70* and *l(2]ef)* RNA levels in response to selected stresses Quantitative real-time RT-PCR analysis was used to determine RNA levels for the genes *Hsp70* and *l(2)efl* in response to selected stresses, in both male and female flies, as indicated. HS, heat stress; IR, ionizing radiation. Stress treatment RNA levels were compared to control using unpaired, two-sided t-tests, and statistically significant differences (p < 0.05) are indicated with asterisk.

These up-regulated genes included the heat shock protein genes *Hsp70*, *Hsp83* (which is the single Drosophila Hsp90-class member), and the small Hsp gene *l(2)efl*. The up-regulation of *Hsp70* and *l(2)efl* in response to selected stressors was confirmed using quantitative real-time PCR analysis (Figure 1), and in addition *Hsp70* was analyzed by Northern blot analysis (Supplemental Figure S1; results summarized in Table 2). Also up-regulated by aging and each stressor were the glutathione S-transferase gene *GstD2*, the central metabolic regulatory enzyme gene *Pepck*, and the mitochondrial unfolded protein response (mUPR) gene *ref(2)P*. Down-regulated genes included several associated with sugar metabolism and proteolysis (Table 1).

**Table 2:** Confirmation of selected gene expression changes using qPCR and Northern analysis

	O_2_ up	O_2_ dn	H_2_O_2_ up	H_2_O_2_ dn	HS up	HS dn	IR up	IR dn	Age up	Age dn
hsp70	XQL		XN		XQ		XQ		XL	
hsp22	XQL		N		XQ		XQ		XL	
l(2)efl	XQL		Xnt		XQ		XQ		XL	
Drs	XQL				X	Q	XQf		XL	
ade3	XQL		Xnt		Q		XQ		XL	
CG11089	XQ		XN		Q		XQ		Xnt	
GstD2	X		X		X		X		X	
GstD1	X		X				X		X^a^	

X GeneChip data (this study); Q q-PCR analysis (this study); N Northern analysis (this study); L Northern analysis (Landis et al 2004 PNAS 101:7663-8); nt not tested.

a fold increase >1.2

### Gene expression changes unique to each stress

Each stress had gene expression changes that were unique to that stress (listed in Supplemental Table S1) and the enriched GO terms that uniquely characterize each stress are summarized (Table 3). Hyperoxia stress had no enriched GO terms in the uniquely up-regulated genes, and a single enriched GO term, Signal peptide processing (3 genes) among the down-regulated genes. In contrast, there were numerous up-regulated genes unique to hydrogen peroxide stress, and these up-regulated genes were enriched for many GO terms involved in developmental pathways, signaling pathways, and nucleobase metabolism (Table 3). Genes uniquely up-regulated upon heat stress included many of the Hsp60-class, and this list was consequently enriched for the GO term Protein folding (16 genes), whereas down-regulated genes unique to heat stress were enriched for the GO terms Defense response and Melanization defense response (Table 3). Finally, genes uniquely up-regulated in response to ionizing radiation included several proteasome subunit genes (Supplemental Table S1), and this gene list was enriched for the GO terms Protein catabolic process and Macromolecular catabolic process (Table 3), whereas there were no GO terms enriched among down-regulated genes.

**Table 3:** Features unique to each stress

**Table:** 

GO:0006465	signal peptide processing(3)	0.014095

**Table:** 

GO:0032501	multicellular organismal process(153)	2.79E-09
GO:0007275	multicellular organismal development(125)	1.11E-08
GO:0065007	biological regulation(143)	4.03E-08
GO:0050794	regulation of cellular process(128)	7.31E-08
GO:0050789	regulation of biological process(134)	1.20E-07
GO:0009653	anatomical structure morphogenesis(84)	1.62E-07
GO:0048856	anatomical structure development(121)	1.45E-06
GO:0050793	regulation of developmental process(39)	4.68E-06
GO:0032502	developmental process(132)	5.08E-06
GO:0048468	cell development(63)	1.34E-05
GO:0048731	system development(41)	4.96E-05
GO:0009790	embryo development(41)	1.13E-04
GO:0040011	Locomotion(35)	0.001096
GO:0048699	generation of neurons(42)	0.001396
GO:0050896	response to stimulus(106)	0.001425
GO:0051239	regulation of multicellular organismal process(34)	0.001519
GO:0045595	regulation of cell differentiation(25)	0.001776
GO:0030182	neuron differentiation(39)	0.00189
GO:0023052	Signaling(79)	0.002054
GO:0007154	cell communication(80)	0.002305
GO:0048666	neuron development(35)	0.003766
GO:0007165	signal transduction(64)	0.00438
GO:0048513	organ development(62)	0.004542
GO:0022414	reproductive process(54)	0.005853
GO:0003002	Regionalization(33)	0.007295
GO:0022603	regulation of anatomical structure morphogenesis(21)	0.00904
GO:2000026	regulation of multicellular organismal development(26)	0.01046
GO:0009880	embryonic pattern specification(21)	0.012338
GO:0009887	organ morphogenesis(37)	0.013194
GO:0007350	blastoderm segmentation(20)	0.01346
GO:0003006	developmental process involved in reproduction(37)	0.018008
GO:0051093	negative regulation of developmental process(16)	0.018841
GO:0010556	regulation of macromolecule biosynthetic process(49)	0.019198
GO:2000112	regulation of cellular macromolecule biosynthetic process(49)	0.019198
GO:0030154	cell differentiation(86)	0.019291
GO:0048869	cellular developmental process(89)	0.022952
GO:0048667	cell morphogenesis involved in neuron differentiation(29)	0.022954
GO:0007423	sensory organ development(31)	0.023396
GO:0051674	localization of cell(21)	0.024068
GO:0019219	regulation of nucleobase-containing compound metabolic process(51)	0.027513
GO:0048609	multicellular organismal reproductive process(45)	0.029739
GO:0007155	cell adhesion(19)	0.029741
GO:0030030	cell projection organization(33)	0.030221
GO:0003008	system process(43)	0.030877
GO:0051171	regulation of nitrogen compound metabolic process(51)	0.031374
GO:0007389	pattern specification process(33)	0.031638
GO:0048477	Oogenesis(33)	0.034694
GO:0048732	gland development(19)	0.034716
GO:0031326	regulation of cellular biosynthetic process(50)	0.036899
GO:0010468	regulation of gene expression(55)	0.037944
GO:0009889	regulation of biosynthetic process(50)	0.038122
GO:0000003	Reproduction(54)	0.043565
GO:0048870	cell motility(20)	0.048342
GO:0007292	female gamete generation(33)	0.049805

**Table:** 

GO:0006457	protein folding(16)	0.018356

**Table:** 

GO:0006582	melanin metabolic process(7)	0.001782
GO:0035006	melanization defense response(6)	0.006478
GO:0006952	defense response(20)	0.00679

**Table:** 

GO:0030163	protein catabolic process(16)	8.21E-05
GO:0009057	macromolecule catabolic process(16)	0.049169

### Aging is most similar to hyperoxia

As described above, a core set of stress response genes was induced during aging and by each of the stressors tested. Aging shared additional changes in gene expression with each individual stressor (Supplemental Table S2), and was found to be more similar to the stresses most associated with oxidative stress (hyperoxia, hydrogen peroxide, ionizing radiation) than it was to heat stress, based on cluster analysis (Supplemental Figure S2) and by comparison of the GO categories that were enriched in the groups of up-regulated and down-regulated genes (Supplemental Table S3). While aging shared a significant overlap in up-regulated and down-regulated genes with each of the stresses, aging shared the greatest number of gene expression changes with hyperoxia (Table 4).

**Table 4:** Number of gene expression changes shared by aging and individual stresses

Aging change	Number genes	Stress change	Number genes	Number in common	*p*
Aging up	456	Hyperoxia up	335	165	9.8 × 10^−170^
Aging up	456	Ionizing radiation up	716	171	2.9 × 10^−114^
Aging up	456	Hydrogen peroxide up	728	133	4.3 × 10^−70^
Aging up	456	Heat stress up	754	66	1.2 × 10^−15^
Aging down	1009	Hyperoxia down	556	222	1.5 × 10^−121^
Aging down	1009	Ionizing radiation down	674	166	3.6 × 10^−54^
Aging down	1009	Hydrogen peroxide down	911	132	1.1 × 10^−18^
Aging down	1009	Heast stress down	806	89	7.3 × 10^−7^

### Gene expression changes unique to aging

A number of gene expression changes were found to be unique to aging. These included up-regulation of numerous innate immune response genes, and down-regulation of numerous mitochondrial metabolism genes, including ones encoding components of the ETC (Supplemental Table S1; enriched GO terms listed in Table 4). While up-regulation of innate immune response genes is a feature of aging that is shared with hyperoxia [6] (Supplemental Table S3), the number of up-regulated innate immune response genes was significantly greater for aging, resulting in many changes in this category that were unique to aging. Also uniquely up-regulated during aging were the odorant receptor genes *Obp56a* and *Obp57d*.

Down-regulation of mitochondrial genes is a feature of aging that is shared with hydrogen peroxide and ionizing radiation (Supplemental Table S3), but the number of down-regulated mitochondrial genes was greater for aging, resulting in many changes in this category that were unique to aging. Among these many down-regulated mitochondrial genes were ones encoding mitochondrial ribosomal proteins and components of the mitochondrial membrane protein translocases (TIM and TOM), as well as the mitochondrial form of superoxide dismutase (*MnSOD* or *Sod2*).

### Confirmation of selected gene expression changes

Changes in gene expression caused by one or more stressors were confirmed by quantitative real-time PCR (Figure 1 and Supplemental Figure S3) and by Northern blot analysis (Supplemental Figure S1), and in general an excellent concordance was observed with the micro-array data and with the published literature (Summarized in Table 2). One exception was for expression of the innate immune response gene *Drosomycin* upon heat stress, which was observed to increase in the micro-array analysis, but to decrease in the qPCR analysis (Table 2). Because bacterial load and *Drosomycin* gene expression can vary significantly between different flies and vials of flies [13], we conclude that this discrepancy was most likely due to a small difference in bacterial load and *Drosomycin* gene expression in the control flies used for the qPCR analysis relative to the control flies used for micro-array analysis.

Comparison of the responses to the different stresses reveals preferential induction of certain genes. For example, *Hsp70* (Figure 1) and *Hsp22* (Supplemental Figure S3) were induced to the greatest extent by heat stress, whereas *l(2)efl* (Figure 1) and *ade3* (Supplemental Figure S3) were induced to a greater extent by ionizing radiation and hyperoxia. In addition significant sexual dimorphism in the magnitude of responses was observed. For example, the induction of *Hsp70* (Figure 1) and *Hsp22* (Supplemental Figure S3) in response to heat stress was greater in males than in females, and the induction of *l(2)efl* (Figure 1) and *ade3* (Supplemental Figure S3) in response to ionizing radiation was greater in males than in females.

### A GstD-GFP reporter construct recapitulates induction during aging

The*GstD1* gene encodes a glutathione-S-transferase, and is induced in adult flies during normal aging and when flies are challenged with oxidative stress produced by hyperoxia and paraquat [6, 7, 14], and was also found to be up-regulated in response to hydrogen peroxide and ionizing radiation stress (Summarized in Table 2). The *GstD1* promoter region contains consensus binding motifs for the stress-responsive transcription factors Nrf2 and Foxo (diagrammed in Supplemental Figure S4). A transgenic reporter has been characterized where the regulatory sequences of the *GstD1* gene are fused to GFP, and the resulting GstD-GFP reporter is induced in the adult fly by feeding flies with the oxidative stressors paraquat, arsenic or hydrogen peroxide [15]. A clustered point mutation was created to disrupt the antioxidant response element (ARE) in the GstD-GFP reporter to yield a mutant reporter called GstD-deltaARE-GFP (diagrammed in Supplemental Figure S4). These reporters have been used to demonstrate that the GstD-GFP transgene is positively regulated in the adult fly in response to genetically-altered Nrf2 expression, and in response to the cancer chemotherapeutic compound Oltipraz which is known to activate Nrf2, and these regulations required the intact ARE [15]. Quantitative PCR analysis of adult flies indicated that induction of the *GstD1* gene by paraquat is reduced in flies hemizygous for the JNKK gene *hemipterous*, suggesting additional positive regulation of GstD genes by the JNK pathway in response to oxidative stress [16]. The JNK pathway activates the transcription factor Foxo suggesting that the JNK pathway may activate GstD gene expression through the Foxo binding motif located in the GstD gene promoter region [17](diagrammed in Supplemental Figure S4). A GstD1-LacZ reporter has been reported to be up-regulated during normal aging in the enteroendocrine cells (ECs) of the fly intestine, but to be reduced during aging in the intestinal stem cells (ISCs) [18].

**Figure 2:**
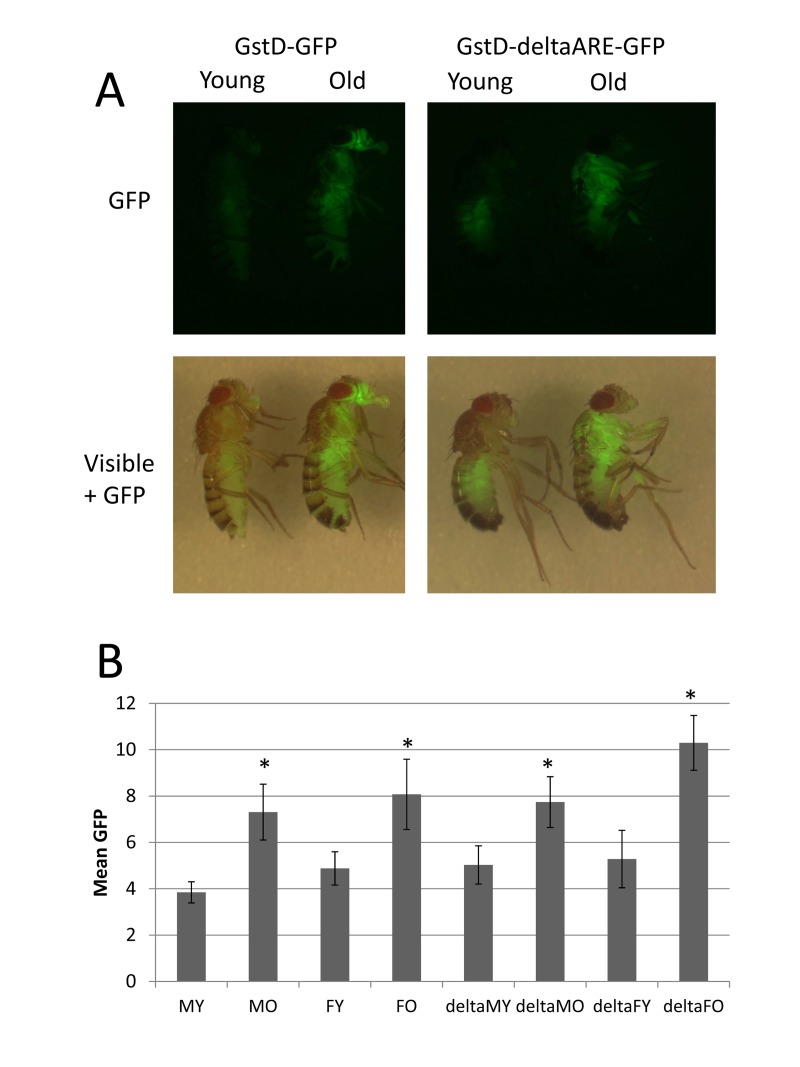
GstD-GFP transgenic reporters recapitulate GstD1 gene induction during aging (**A**) Expression of the transgenic reporter constructs GstD-GFP and GstD-deltaARE-GFP was visualized in 6 day old (Young) and 30 day old (Old) male and female flies, as indicated, using the fluorescence stereomicroscope. The GFP image and an overlay of the GFP image and the visible light image are presented, as indicated. (**B**) Quantification of the expression of the GstD-GFP and GstD-deltaARE-GFP reporters in male (M) and female (F) flies, both young (Y) and old (O), as indicated. The data for the Gst-deltaARE-GFP reporter is specified by prefix (delta). Values for old flies were compared to young using unpaired, two-sided t-tests, and statistically significant differences (p < 0.05) are indicated with asterisk.

Here the expression of the GstD-GFP and GstD-deltaARE-GFP reporters were examined in whole adult flies during normal aging. The GstD-GFP reporter was expressed at low levels in young flies, and exhibited robust induction throughout the body of the fly during aging, including the head, thorax, abdomen and legs (Figure 2A), consistent with the whole-body micro-array analyses presented above. Induction was apparent even at moderate ages (30 days; Figure 2A) and continued at high levels for the remainder of the life span (data not shown). In contrast, the GstD-deltaARE-GFP reporter was robustly induced in thorax and legs, particularly in flight muscle and leg muscle, whereas induction in the head and abdomen was either greatly reduced or absent. The GstD-deltaARE-GFP reporter was also observed to produce slightly more expression in young flies in the upper abdomen. The mean GFP intensity throughout the body was quantified from captured images of multiple flies using Image J software, and this analysis confirmed the up-regulation of both reporters during aging, in both males and females (Figure 2B). Despite the absence of GFP induction in head and abdomen tissue, the mean intensity of fluorescence produced by the GstD-deltaARE-GFP reporter throughout the fly was comparable to the un-mutated reporter (Figure 2B), as the expression in thorax and legs was relatively greater (see Figure 2A); and this difference may be due to some effect of the different chromosomal insertion sites on the overall expression levels for the reporters. Taken together, these data confirm the up-regulation of the *GstD1* gene during aging in the majority of adult tissues, and suggest that efficient expression in the head and abdomen may require the consensus ARE motif (Diagrammed in Supplemental Figure S4).

## DISCUSSION

### A core set of stress response genes shared with aging

Changes in gene expression that were common to aging and all tested stresses identified a core set of stress response genes. *Pepck* encodes an enzyme critical in gluconeogenesis and glyceroneogenesis, and its up-regulation may be part of a basic metabolic adaptation to stress [19]. Interestingly, in mice, over-expression of PEPCK specifically in muscle tissue increases movement, life span and muscle mitochondrial proliferation [20]. Also among the core set of induced genes were *Starvin, ref(2)P*, and the Hsp genes *Hsp70, Hsp83* and *l[2]efl*. *Starvin* encodes a co-chaperone involved in autophagy and muscle maintenance and its up-regulation is consistent with its role in protein turnover and the cellular response to proteotoxicity [21]. Similarly, *ref(2)P* encodes a component of the mitochondrial unfolded protein response (mUPR) pathway, consistent with proteotoxicity in the mito-chondrial compartment. Hsp genes are induced in response to protein denaturation and misfolding through activation of the HSF transcription factor, which in turn binds to HSEs in the Hsp gene promoters and activates transcription [1, 2]. In stressed cells certain Hsps have been shown to function to reduce proteotoxicity by favoring protein re-folding as well as the turnover of damaged proteins through the ubiquitin/proteasome and autophagy pathways [2, 22]. Induction of Drosophila Hsp genes during normal aging and upon oxidative stress has been shown to be dependent upon functional HSEs in the gene promoters, consistent with an increased abundance of misfolded proteins and conse-quent HSF activation under these conditions [4, 5]. The presence of Hsp genes in the set up-regulated by aging and each stressor indicates that protein denaturation/misfolding and HSF activation are common features of aging and each of the tested stresses. Induction of Hsps during aging may be part of a stress response that favors fly function by helping the fly to cope with aging-associated proteotoxicity [3]. Consistent with this idea, increased expression of certain Hsps is associated with increased fly life span [23-25]. In addition, it is also possible that chronic Hsp induction, particularly at late ages, may sometimes be mal-adaptive [2]. Interestingly, 8/18 of the common up-regulated genes and 21/32 of the common down-regulated genes have uncharacterized functions, indicating that there is much yet to be learned about the core stress response.

**Table 5:** Features unique to aging

**Table:** 

GO:0006952	defense response(24)	1.04E-10
GO:0042742	defense response to bacterium(17)	2.52E-09
GO:0009617	response to bacterium(17)	2.61E-08
GO:0050830	defense response to Gram-positive bacterium(11)	1.35E-07
GO:0051707	response to other organism(19)	1.29E-06
GO:0009607	response to biotic stimulus(19)	1.55E-06
GO:0006955	immune response(18)	4.36E-06
GO:0002376	immune system process(20)	1.56E-05
GO:0051704	multi-organism process(21)	7.84E-04
GO:0019731	antibacterial humoral response(8)	0.002999
GO:0009620	response to fungus(7)	0.010042
GO:0006959	humoral immune response(11)	0.015651
GO:0019730	antimicrobial humoral response(10)	0.039404

**Table:** 

GO:0006091	generation of precursor metabolites and energy(53)	6.12E-32
GO:0045333	cellular respiration(44)	3.06E-29
GO:0015980	energy derivation by oxidation of organic compounds(44))	2.36E-27
GO:0042773	ATP synthesis coupled electron transport(29)	9.71E-20
GO:0006119	oxidative phosphorylation(30)	1.06E-19
GO:0022900	electron transport chain(30)	1.09E-18
GO:0022904	respiratory electron transport chain(29)	1.88E-18
GO:0042775	mitochondrial ATP synthesis coupled electron transport(27)	4.70E-18
GO:0055114	oxidation-reduction process(62)	8.74E-10
GO:0006084	acetyl-CoA metabolic process(17)	1.56E-09
GO:0006099	tricarboxylic acid cycle(16)	6.58E-09
GO:0046356	acetyl-CoA catabolic process(16)	6.58E-09
GO:0009109	coenzyme catabolic process(16)	1.02E-08
GO:0009060	aerobic respiration(16)	1.57E-08
GO:0051187	cofactor catabolic process(16)	1.57E-08
GO:0006732	coenzyme metabolic process(21)	3.37E-07
GO:0006120	mitochondrial electron transport, NADH to ubiquinone(13)	7.68E-07
GO:0051186	cofactor metabolic process(22)	1.21E-06
GO:0016310	Phosphorylation(47)	1.84E-06
GO:0006793	phosphorus metabolic process(53)	7.47E-06
GO:0006796	phosphate-containing compound metabolic process(53)	7.47E-06
GO:0006839	mitochondrial transport(13)	3.31E-04
GO:0006123	mitochondrial electron transport, cytochrome c to oxygen(7)	0.002098
GO:0009056	catabolic process(46)	0.003113
GO:0044281	small molecule metabolic process(58)	0.003288
GO:0007005	mitochondrion organization(15)	0.003544
GO:0006626	protein targeting to mitochondrion(9)	0.005904
GO:0070585	protein localization in mitochondrion(9)	0.005904
GO:0072655	establishment of protein localization in mitochondrion(9)	0.005904
GO:0007283	Spermatogenesis(22)	0.007147
GO:0048232	male gamete generation(22)	0.007884
GO:0043648	dicarboxylic acid metabolic process(7)	0.010686
GO:0006096	Glycolysis(8)	0.02534
GO:0006122	mitochondrial electron transport, ubiquinol to cytochrome c(6)	0.026633
GO:0044248	cellular catabolic process(36)	0.032559
GO:0006006	glucose metabolic process(10)	0.042632

### Each stress has unique gene expression changes

While the pattern of gene expression changes during aging shared features with each of the tested stresses, each stress also had unique features (Summarized in Table 3). For example, hydrogen peroxide stress caused up-regulation of numerous genes involved in developmental pathways and signaling pathways, consistent with the fact that hydrogen peroxide also normally functions as a signaling molecule, during development and in adults, in Drosophila and other metazoans [26-30]. Genes uniquely up-regulated upon heat stress included ones of the Hsp60-class, which are important for protein trafficking and protein import into organelles [31]. Down-regulated genes unique to heat stress were enriched for the GO terms Defense response and Melanization defense response, suggesting that responses to wounding and bacterial challenge may be impaired. Finally, genes uniquely up-regulated in response to ionizing radiation included several proteasome subunit genes, which may indicate a particular requirement for protein turnover, perhaps in response to protein backbone cleavage, or alternatively this might reflect the critical role of the proteasome in DNA repair [32].

### Aging has both shared and unique features relative to the tested stresses

While aging shared features with each stress, aging was found to be more similar to the stresses most associated with oxidative stress (hyperoxia, hydrogen peroxide, ionizing radiation) than it was to heat stress. These observations are consistent with the conclusion that aging eukaryotic cells are in a pro-oxidant state [6, 33] associated with up-regulation of oxidative stress-response genes including ones encoding Gsts [34]. In addition to the shared features, a number of gene expression changes were found to be unique to aging (Supplemental Table S1). For example, the gene encoding the mitochondrial form of superoxide dismutase (*MnSOD* or *SOD2*) was uniquely down-regulated during aging, and this is of potential interest given the fact that augmenting the expression of*MnSOD* can favor life span in adult flies [35, 36] and in *C. elegans*[37]. Also uniquely up-regulated during aging were the odorant receptor genes *Obp56a* and *Obp57d*, which is interesting in light of reports of negative effects of other odorant receptor genes on fly life span [38].

**Table 6:** Features common to aging and individual stress factors

**Table:** 

GO:0009408	response to heat(16)	2.64E-11
GO:0009266	response to temperature stimulus(17)	1.15E-10
GO:0006950	response to stress(34)	9.95E-08
GO:0035079	polytene chromosome puffing(5)	3.88E-05
GO:0035080	heat shock-mediated polytene chromosome puffing(5)	3.88E-05
GO:0009628	response to abiotic stimulus(17)	8.38E-05
GO:0044271	cellular nitrogen compound biosynthetic process(14)	0.00202
GO:0034605	cellular response to heat(6)	0.002657
GO:0019731	antibacterial humoral response(7)	0.009046
GO:0009156	ribonucleoside monophosphate biosynthetic process(5)	0.032356
GO:0009161	ribonucleoside monophosphate metabolic process(5)	0.032356
GO:0006564	L-serine biosynthetic process(3)	0.04487

**Table:** 

GO:0006508	Proteolysis(56)	6.01E-18
GO:0045297	post-mating behavior(7)	9.33E-04
GO:0008152	metabolic process(131)	0.00614

**Table:** 

GO:0009408	response to heat(11)	4.75E-06
GO:0035079	polytene chromosome puffing(5)	1.44E-05
GO:0035080	heat shock-mediated polytene chromosome puffing(6)	1.44E-05
GO:0006950	response to stress(27)	2.86E-05
GO:0009266	response to temperature stimulus(11)	7.87E-05
GO:0034605	cellular response to heat(6)	8.32E-04
GO:0044271	cellular nitrogen compound biosynthetic process(13)	0.001277
GO:0009069	serine family amino acid metabolic process(5)	0.005345
GO:0044281	small molecule metabolic process(24)	0.005367
GO:0006564	L-serine biosynthetic process(3)	0.024923
GO:0051707	response to other organism(11)	0.032029
GO:0009607	response to biotic stimulus(11)	0.035404

**Table:** 

GO:0006091	generation of precursor metabolites and energy(12)	0.001493
GO:0022900	electron transport chain(9)	0.001518
GO:0055114	oxidation-reduction process(22)	0.002986
GO:0045333	cellular respiration(10)	0.003694
GO:0015980	energy derivation by oxidation of organic compounds(10)	0.008153
GO:0022904	respiratory electron transport chain(8)	0.010626
GO:0042775	mitochondrial ATP synthesis coupled electron transport(7)	0.043253

**Table:** 

GO:0009408	response to heat(18)	1.04E-21
GO:0009266	response to temperature stimulus(18)	1.68E-19
GO:0009628	response to abiotic stimulus(18)	8.84E-13
GO:0006950	response to stress(22)	1.70E-08
GO:0006457	protein folding(11)	1.90E-07
GO:0035079	polytene chromosome puffing(5)	4.29E-07
GO:0035080	heat shock-mediated polytene chromosome puffing(5)	4.29E-07
GO:0034605	cellular response to heat(6)	1.27E-05
GO:0001666	response to hypoxia(6)	0.001193
GO:0070482	response to oxygen levels(6)	0.002157
GO:0042221	response to chemical stimulus(14)	0.031137

**Table:** 

GO:0006508	Proteolysis(25)	1.75E-07

**Table:** 

GO:0006950	response to stress(33)	2.17E-06
GO:0009408	response to heat(12)	5.07E-06
GO:0035079	polytene chromosome puffing(5)	5.04E-05
GO:0035080	heat shock-mediated polytene chromosome puffing(5)	5.04E-05
GO:0009266	response to temperature stimulus(12)	1.05E-04
GO:0034605	cellular response to heat(6)	0.003611
GO:0009069	serine family amino acid metabolic process(5)	0.018171
GO:0044271	cellular nitrogen compound biosynthetic process(13)	0.022327
GO:0033554	cellular response to stress(18)	0.024714

**Table:** 

GO:0006091	generation of precursor metabolites and energy(17)	2.30E-07
GO:0045333	cellular respiration(14)	1.95E-06
GO:0015980	energy derivation by oxidation of organic compounds(14)	6.16E-06
GO:0022900	electron transport chain(11)	4.47E-05
GO:0055114	oxidation-reduction process(26)	2.65E-04
GO:0006119	oxidative phosphorylation(10)	2.91E-04
GO:0022904	respiratory electron transport chain(10)	2.91E-04
GO:0042775	mitochondrial ATP synthesis coupled electron transport(9)	9.52E-04
GO:0042773	ATP synthesis coupled electron transport(9)	0.00164

### The aging gene expression pattern indicates a failure in mitochondrial maintenance

The changes in gene expression that were found to be unique to aging included up-regulation of numerous innate immune response genes, and down-regulation of numerous mitochondrial metabolism genes, including ones encoding components of the ETC (Table 4). While up-regulation of innate immune response genes is a feature of aging that is shared with hyperoxia [6] (Supplemental Table S3), the number of up-regulated innate immune response genes was significantly greater for aging, resulting in many changes in this category that were unique to aging. Similarly, down-regulation of mitochondrial and ETC genes is a feature of aging that is shared with ionizing radiation and hydrogen peroxide stress (Supplemental Table S3), but the number of down-regulated mitochondrial and ETC genes was greater for aging, resulting in many changes in this category that were unique to aging. Girardot et al [39] examined gene expression changes during Drosophila aging separately for the head, thorax and abdomen, and found that down-regulation of mitochondrial genes is observed preferentially in the thorax; because the thorax is composed primarily of flight muscle this observation suggests that mitochondrial gene down-regulation may occur preferentially in muscle tissue.

Up-regulation of innate immune response genes during Drosophila aging is in part due to a dramatic increase in microbial load during aging, as eliminating bacteria reduces the response [13]. However, innate immune response genes are still up-regulated during aging in the absence of detectable microbes, suggesting additional mechanisms for activation of these genes during aging. Consistent with this conclusion, innate immune response genes are also up-regulated in response to oxidative stress caused by hyperoxia ([6]; this study), and therefore one possibility is that an aging-related failure in mitochondrial maintenance leads to oxidative stress that can induce innate immune response gene expression. Similarly, studies in mammals reveal that damaged mitochondria also release DNA fragments and formyl-peptides that can induce innate immune response genes [40], and therefore this may be an additional mechanism for innate immune response gene induction during aging that is a consequence of a failure in mitochondrial maintenance. The across-the-board down-regulation of Drosophila mitochondrial genes, ETC genes and mitochondrial metabolism genes observed during aging suggests a possible mechanism for a failure in mitochondrial maintenance during aging (Diagrammed in Supplemental Figure S5). The ETC and mitochondria turn over at a basal rate, and more rapidly in response to signals such as starvation, and a reduced rate of replacement is expected to result in longer-lived structures that will be more susceptible to time-dependent damage and malfunction. This idea is consistent with the observed accumulation of structural-ly abnormal mitochondria during Drosophila aging [41-44], reduced mitochondrial transcription [45], decreased ATP and increased production of ROS [46]. Decreased ATP flux is expected to reduce rates of bulk protein synthesis and turnover, and increased ROS will increase protein damage, consistent with the accumulation of damaged and misfolded proteins (proteotoxicity) and the induction of Hsp genes [2, 22, 24].

Taken together, the data support a model wherein the down-regulation of mitochondrial and ETC genes during aging leads to a failure in mitochondrial maintenance and the accumulation of abnormal mitochondria, which in turns leads to oxidative stress and proteotoxicity; these stresses in turn cause the oxidative-stress-like and proteotoxic-stress-like patterns of gene expression observed during aging (Supplemental Figure S5). Placing oxidative stress down-stream of an aging-associated failure in mitochondrial maintenance is consistent with the observation that oxidative stress correlates with, but does not directly regulate life span in Drosophila [47], and with the implication of mitochondrial malfunction in mammalian aging-related metabolic disorders [48]. Consistent with the importance of mitochondrial maintenance in aging, certain interventions that increase mitochondrial proliferation, such as over-expression of PGC1alpha in gut tissue, have recently been reported to increase life span and tissue function in aging Drosophila [49, 50], and PGC1alpha activity is also implicated in maintaining tissue function during aging in mammals [51]. In contrast, other manipulations that increase Drosophila mitochondrial proliferation, such as increased tissue-general expression of PGC1alpha [50] or cyclin D/Cdk4 [52] had negative consequences for life span and oxidative stress levels, and taken together these studies indicate that effective interventions in mitochondrial maintenance during aging will require tissue-specific targeting. Notably, certain carefully-timed interventions that reduce activity of ETC components have been shown to increase life span in both invertebrates and mammals [53-55], and this might function through a hormetic response to increase production of new mitochondria, or conceivably by inhibiting the activity of abnormal mitochondria. Critical questions for the future include determining the causes and mechanisms for the observed down-regulation of mitochondrial and ETC genes during aging - a pattern shared by Drosophila and mammalian tissues [6, 9]. Possible explanations include the inherently shorter-lived nature of mitochondrial genome sequences relative to nuclear genome sequences, genetic conflicts resulting from the uni-parental inheritance of mitochondrial genomes, and trade-offs between the costly production of new mitochondria and investments in growth, sexual differentiation and reproduction [56-61](Supplemental Figure S5), and these will be interesting areas for future research.

## METHODS

### Drosophila culture, microscopy and stress treatments

*Drosophila melanogaster* flies were cultured on a standard agar/dextrose/corn meal/yeast media at 25^°^C [62]. The transgenic strains GstD1-GFP and GstD1-deltaARE-GFP were generously provided by Dirk Bohmann [15]. Age-synchronized cohorts of flies were generated by collecting newly-eclosed flies over a period of 48 hours, followed by maintenance in vials at approximately 20 flies per vial, with every-other day transfer to fresh media, until the indicated age time points. Visible images, GFP fluorescence images, and image overlays for flies were generated using the Leica MZFLIII fluorescence stereomicroscope. GFP fluore-scence was quantified using captured GFP images and Image J software, with mean and standard deviation calculated using 6 flies per sample. Flies used for stress treatments, RNA analyses and microarray analyses were generated as follows: males of wild-type strain Oregon-R were crossed to virgins of transgenic laboratory stock *w[1118];rtTA(3)E2/TM3 Sb* to generate hybrid progeny of genotype *w[1118];rtTA(3)E2/+*, as was used for previous microarray analyses [63], and 9-10 day-old male flies were used for each stress treatment. Old flies were 61 days of age, which corresponds to approximately the 50% survival point for the cohort [6]. Vials containing 1% sucrose were prepared by adding 1.5 ml of 1% sucrose in deionized water to a Drosophila vial containing a single folded Kimwipe (Kimberly-Clark). For each stress treatment and the sugar-treated controls, replicate vials of 25 flies each were subjected to the treatment, and then the flies from each vial were separately processed for RNA, and each sample was used to generate probe for one micro-array hybridization, such that each treatment is represented by at least three biological replicates. For hyperoxia treatment flies in standard food vials were subjected to 100% oxygen atmosphere for 5 days as previously described [6]. For ionizing radiation treatment flies in standard food vials were irradiated with 5666Rads/hour for 16 hours using a Cesium source (Grammacell 40-Cesium 137, Atomic Energy, Canada) at the USC Norris Cancer Center facility, and then transferred to 1% sucrose vials for two days followed by processing for RNA. For qPCR analysis 9 hour irradiation samples were also generated. Because ionizing radiation is inhibitory to transcription, the two-day recovery period was included to allow the gene expression response to develop; recovery in sucrose vials was employed because the newly-irradiated flies have greatly reduced mobility and will adhere to the surface of a regular food vial. For hydrogen peroxide treatment flies were placed in sucrose vials adjusted to 3% hydrogen peroxide for two days, and then processed for RNA. For heat stress treatment flies were placed in sucrose vials at 37^o^C for 5.5 hours and then processed for RNA. Controls for the effects of sucrose vials (“sugar-treated controls”) were generated by placing flies in sucrose vials for two days prior to processing for RNA.

### RNA isolation and microarray hybridization

An average of 35 μg RNA was isolated from groups of 25 adult male flies using Trizol reagent (Life Technologies, Grand Island) according to the manufacturer's instructions. The RNA was further purified using the RNAqueous kit, and concentration was determined using NanoDrop spectrophotometer. A portion of the RNA (3 μg) was fractionated on 1.0% agarose gels to determine purity. 10 μg of total RNA was then used as substrate to generate biotinylated cRNA according to standard Affymetrix protocol (Childrens Hospital, Los Angeles, CA)[6]. A total of 35 Affymetrix gene chips were analyzed including at least four biological replicates for each experimental condition and control, with the exception of heat stress in which one array was omitted due to poor quality. The old, hyperoxia, and young samples were derived from our previous study [6] in which six arrays were used for the hyperoxia and young conditions and four arrays were used for old flies. Quantitative real-time RT-PCR analyses [64] and Northern blot analyses [65] were performed as previously described, using RNA samples derived independently from those used for the microarrays.

### Statistical analysis of microarray data

Gene expres-sion measures were computed based on a non-linear multi-chip model of the perfect match signal [66]. This approach enables the separation of specific and non-specific components of the microarray signal and circumvents the issue of saturation bias in the high-intensity range. The background and concentration parameters were both fit within a single global routine (rather than estimating the background parameter before computing gene expression measures), and the model that best described the observed data selected. Linear modeling and empirical Bayes analysis [67] was performed in the R statistical programming language (http://www.r-project.org/) using the Limma: Linear Models for Microarray Data package [67] to identify genes significantly differentially expressed in response to multiple stressors or during aging; Limma computes an empirical Bayes adjustment for the t-test. Because the identification of genes altered in multiple condiions was a major objective of this study, a nested F-test approach was employed as this can be more powerful at detecting genes altered in multiple contrasts. Multiple testing was corrected for using the Benjamini and Hochberg method, which controls the false discovery rate (FDR) [68] in this framework on a per-gene basis (but not across contrasts). Using this robust method, genes were found to be significantly differentially expressed both by biological and statistical criteria (±1.2 fold change, FDR 1% (*p* < 0.01); (Supplemental Table S4a, Supplemental Table S4b, Supplemental Table S4c, Supplemental Table S4d, Supplemental Table S4e, Supplemental Table S4f, Supplemental Table S4g, Supplemental Table S4h, Supplemental Table S4i, Supplemental Table S4j, Supplemental Table S4k, Supplemental Table S4l). Gene expression changes of ±1.5 fold were used for subsequent comparisons, as indicated. Hierarchical cluster analysis of the top 1000 differentially expressed genes for each condition based on the F-test *p*-value from the linear model fit was performed to visualize the gene expression patterns across different stressors, using the R package mclust. The microarray data discussed in this study have been deposited in the National Center for Biotechnology Information Gene Expression Omnibus (GEO) [69] and are accessible through GEO Series numberGSEXXX.

### Functional annotation and statistical overrepresentation of Gene Ontology classifications

Statistically over-represented GO categories were identified using Flymine [70], by the calculation of a *p*-value denoting the probability that the observed numbers of counts could have resulted from randomly distributing a particular GO term between the test and the reference group. Multiple testing was controlled for using the Holm-Bonferoni method.

### Statistical significance of overlapping gene sets

The statistical significance of the overlap between various gene sets was evaluated by computing the *p*-value representing the probability of obtaining the observed number of overlaps by chance under a hypergeometric distribution, using the R function phyper [71].

### Identification of enriched GO terms and corrections for effects of sucrose vials

Gene annotations for the AffyDrosGenome1 arrays were updated to the latest information from Flybase using the online tool Flymine [70] for all genes with expression altered ≥1.5 fold. Gene annotations identified by Flymine as matching more than one entry in the current database were resolved where possible, as follows: The Affymetrix probe ID was obtained from the limma files, and the corresponding probe sequence was obtained from the Affymetrix website. The probe sequence was then used to query the current Drosophila genome sequence annotation using the Flybase website and BLAST function to identify the correct gene. Ten probe sequences had ambiguous match that could not be resolved and were not included in the GO term analyses (Supplemental Table S5), and an additional 27 identifiers did not match genes in the current database. To control for any possible effects on gene expression patterns caused by two days maintenance of flies in sucrose vials, GeneChip analysis was performed on flies transferred to sucrose vials for two days in the absence of added stressors as a control. 258 genes were found to be up-regulated and 362 genes were found to be down-regulated relative to controls maintained on normal media (Supplemental Table S6), and these gene sets had no GO terms enriched among the up-regulated genes, and 4 GO terms enriched among the down-regulated genes: Proteolysis, Post-mating behavior, Insemination, and Lipid metabolic process (Supplemental Table S7). The genes that were up-regulated and down-regulated in response to sucrose were subtracted from the lists of genes up-regulated and down-regulated in response to hydrogen peroxide and ionizing radiation treatment to generate the final lists presented in Tables 1, 3, 4, and Supplemental Table S1, Supplemental Tables S2, Supplemental Table S3. While this simple subtraction procedure does not account for possible gene expression changes caused by interactions of sucrose with the stressors, we observe that the major GO term categories enriched in the gene sets up-regulated and down-regulated by hydrogen peroxide and ionizing radiation do not differ significantlywhen the gene expression changes caused by sucrose alone are included or excluded from the analysis (compare Supplemental Table S8 and Supplemental Table S9 where the effects of sucrose are included, to Supplemental Table S10, Supplemental Tables S11 where the effects of sucrose are excluded). In addition, cluster analysis demonstrated that the gene expression changes caused by the hydrogen peroxide stress treatment and ionizing radiation stress treatment were more similar to each other and to aging and hyperoxia than they were to the sucrose-treated control flies (Supplemental Figure S3), providing further evidence that the gene expression changes due to sucrose transfer do not make a significant contribution to the gene expression changes observed in the hydrogen peroxide and ionizing radiation samples.

## SUPPLEMENTARY MATERIAL














































