# Supplementary material for: Gene expression changes in response to aging compared to heat stress, oxidative stress and ionizing radiation in Drosophila melanogaster
Source: Aging (Albany NY). 2012 Nov 30;4(11):768–89. doi: 10.18632/aging.100499 (PMC3560439; doi:10.18632/aging.100499)
Supplement: Supplementary file 1 [file aging-04-768-s001.pdf]

63. Bieschke ET, Wheeler JC and Tower J. Doxycycline-induced transgene expression during Drosophila development and aging. Mol Gen Genet. 1998; 258:571-579.
64. Shen J and Tower J. Drosophila *foxo* acts in males to cause sexual-dimorphism in tissue-specific *p53* life span effects. Exp Gerontol. 2010; 45:97-105.
65. Ford D, Hoe N, Landis GN, Tozer K, Luu A, Bhole D, Badrinath A and Tower J. Alteration of Drosophila life span using conditional, tissue-specific expression of transgenes triggered by doxycyline or RU486/Mifepristone. Exp Gerontol. 2007; 42:483-497.
66. Abdueva D, Skvortsov D and Tavare S. Non-linear analysis of GeneChip arrays. Nucleic Acids Res. 2006; 34:e105.
67. Smyth GK, Michaud J and Scott HS. Use of within-array replicate spots for assessing differential expression in microarray experiments. Bioinformatics. 2005; 21:2067-2075.
68. Storey JD and Tibshirani R. Statistical methods for identifying differentially expressed genes in DNA microarrays. Methods Mol Biol. 2003; 224:149-157.
69. Barrett T, Suzek TO, Troup DB, Wilhite SE, Ngau WC, Ledoux P, Rudnev D, Lash AE, Fujibuchi W and Edgar R. NCBI GEO: mining millions of expression profiles--database and tools. Nucleic Acids Res. 2005; 33:D562-566.
70. Lyne R, Smith R, Rutherford K, Wakeling M, Varley A, Guillier F, Janssens H, Ji W, McLaren P, North P, Rana D, Riley T, Sullivan J, et al. FlyMine: an integrated database for Drosophila and Anopheles genomics. Genome Biol. 2007; 8:R129.
71. RDevelopmentCoreTeam. (2009). R: A language and environment for statistical computing. R Foundation for Statistical Computing, Vienna, Austria.

SUPPLEMENTAL FIGURES

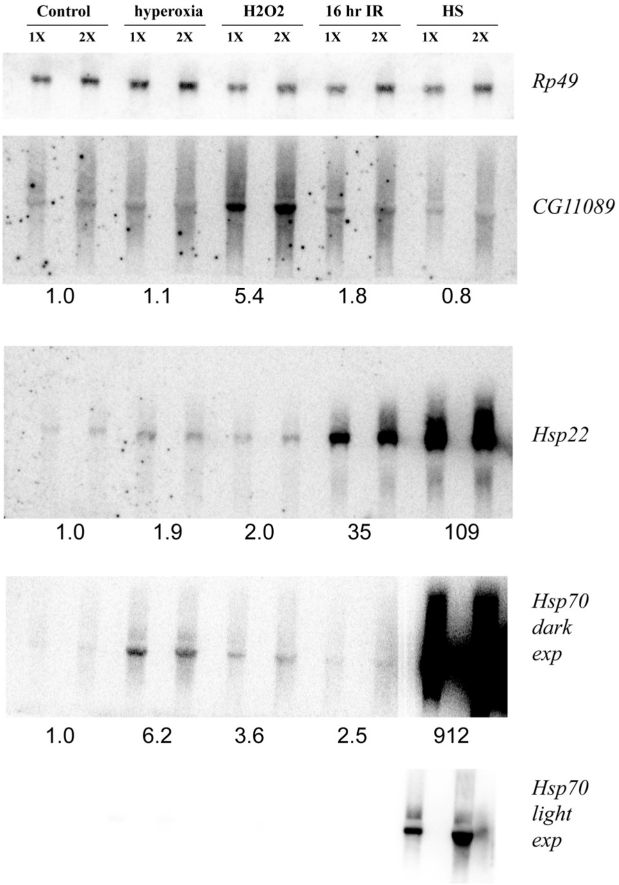

Figure S1. Northern analysis of gene expression in response to selected stresses. Total RNA was isolated from flies subjected to each of the indicated stresses, and 4ug (1X) and 8ug (2X) quantities were analyzed for each sample, as indicated. The blot was hybridized successively with the indicated gene-specific probes, and visualized by autoradiography. Signals were quantified using the phosphorimager, and the fold induction relative to control was calculated for both the 1X and 2X samples, the two values were averaged, and the result is presented below the lanes. For the *Hsp70* gene-specific probe, both a darker exposure and lighter exposure are presented, as indicated. H2O2, hydrogen peroxide.

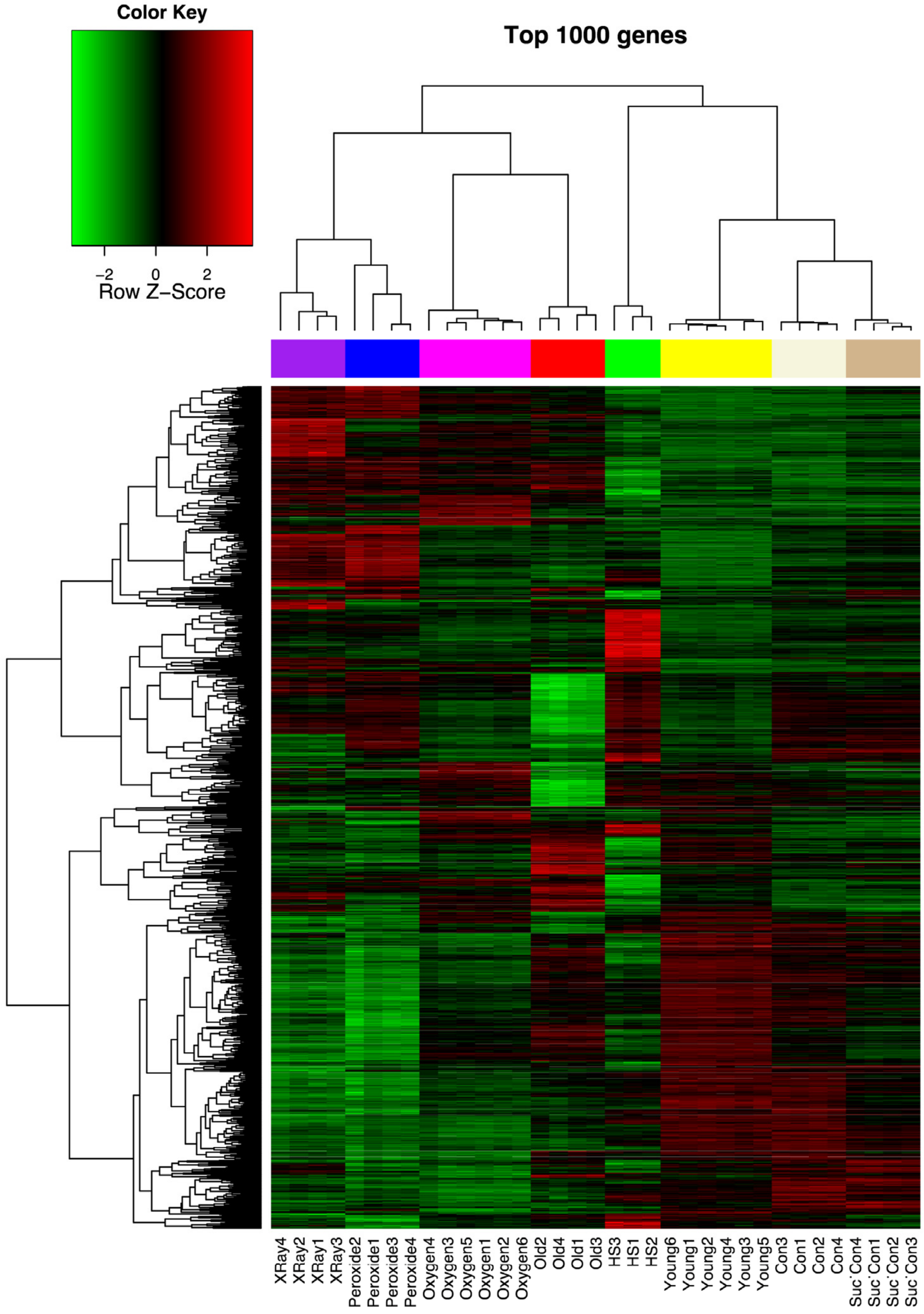

Figure S2. Cluster analysis and heatmap. A heatmap visualization of the gene expression levels across each condition for the top 1000 differentially expressed genes (according to the F-test *p*-value from the linear model fit). XRay, ionizing radiation.

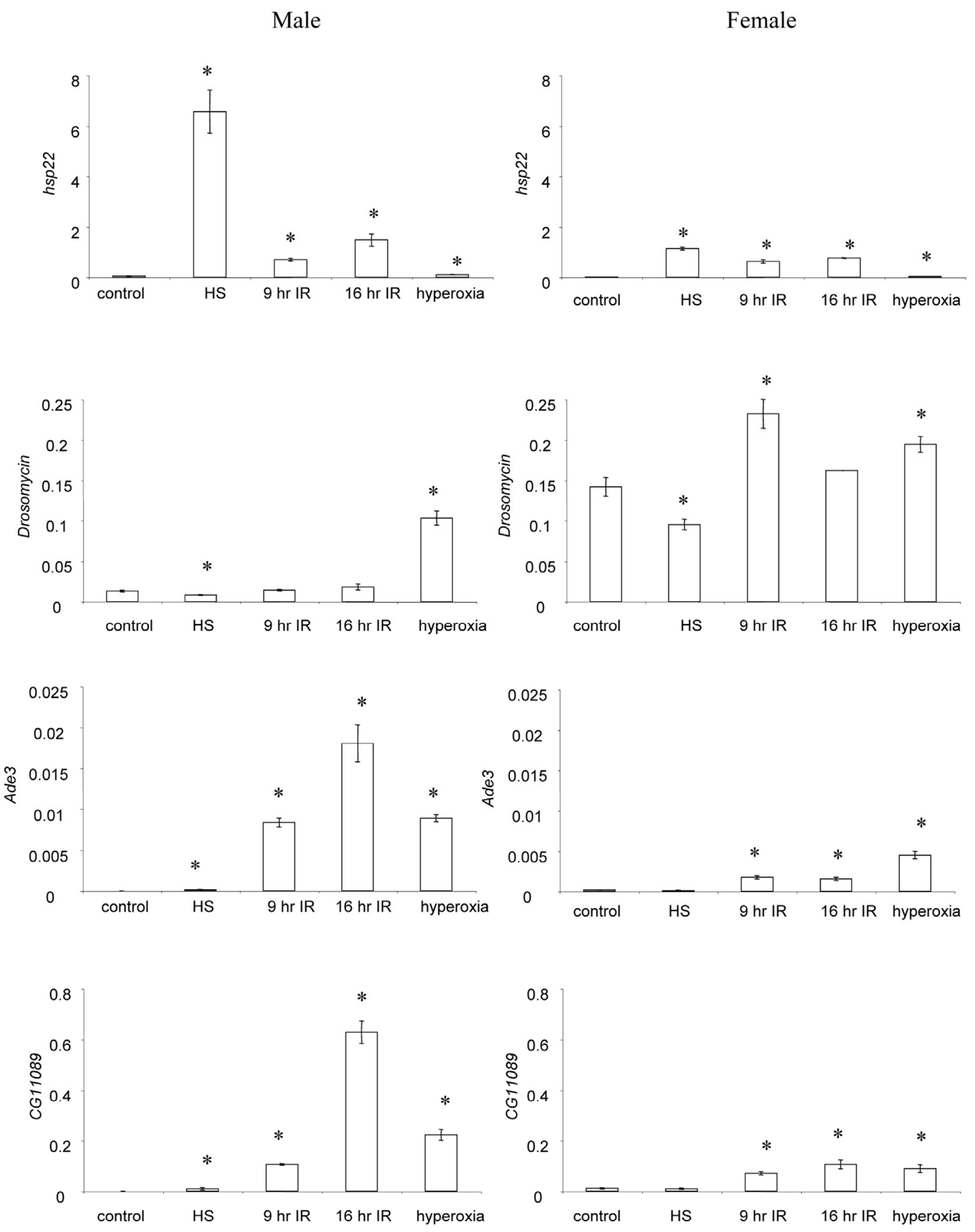

Figure S3. Gene RNA levels in response to selected stresses. Quantitative real-time RT-PCR analysis was used to determine RNA levels for the genes *Hsp22, Drosomycin, Ade3* and *CG11089* in response to selected stresses, in both male and female flies, as indicated. HS, heat stress; IR, ionizing radiation. Stress treatment RNA levels were compared to control using unpaired, two-sided t-tests, and statistically significant differences ($p < 0.05$) are indicated with asterisk.

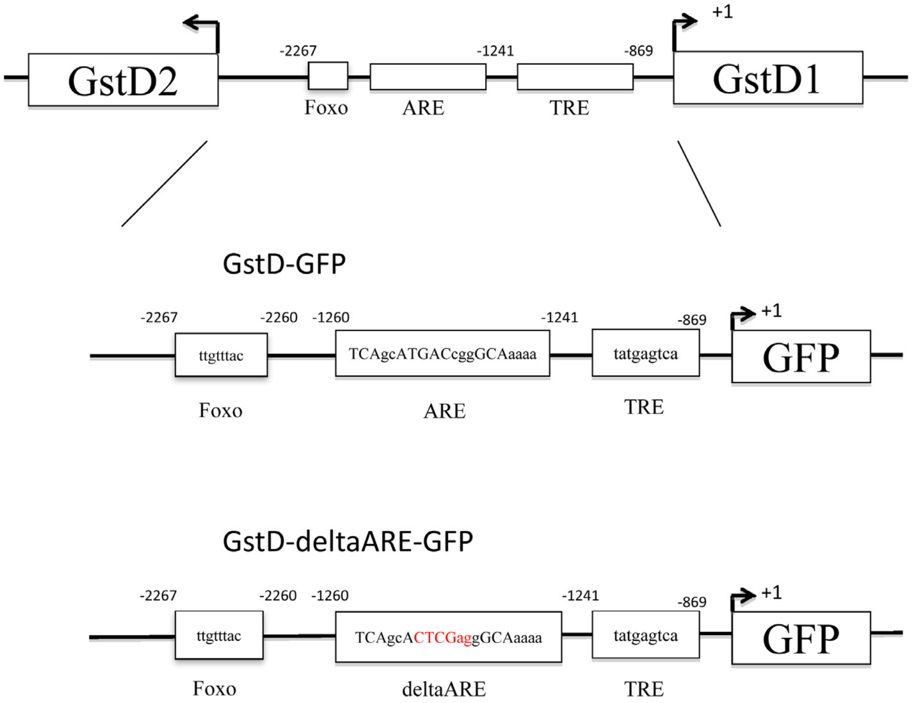

Figure S4. Diagram of control and mutant GstD1-GFP reporters. Diagram of the genomic region containing the *GstD2* and *GstD1* genes and the structure of the GstD-GFP and GstD-deltaARE-GFP reporter constructs. The conserved Foxo and ARE motifs are indicated; red lettering indicates the bases mutated in the deltaARE construct. Numbering is relative to the +1 start site for transcription of the *GstD1* gene; the diagram is not to scale.

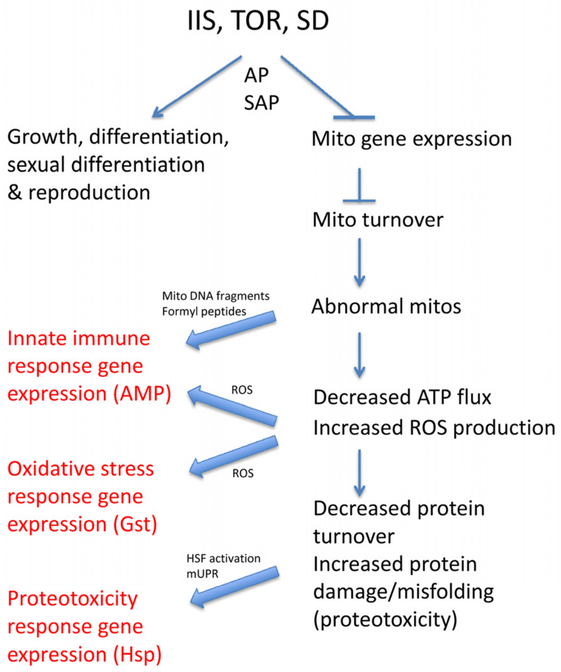

Figure S5. Model for gene expression changes during aging. The genes that comprise the Insulin/IGF1-like signaling pathway (IIS), the TOR signaling pathway (TOR) and the sex-determination pathway (SD) exhibit antagonistic pleiotropy (AP) and sexual antagonistic pleiotropy (SAP), in that these pathways act to promote growth, differentiation, sexual differentiation and reproduction at the expense of mitochondrial (Mito) gene expression and mitochondrial turnover. Decreased mitochondrial turnover results in the accumulation of abnormal and malfunctioning mitochondria, and in turn decreased ATP flux and increased ROS production. Decreased protein turnover due to decreased ATP flux and increased protein damage due to increased ROS production combine to increase levels of protein damage and misfolding, leading to proteotoxicity. Release of mitochondrial DNA fragments, formyl peptides and ROS from abnormal mitochondria (combined with increased microbial load) cause dramatic induction of innate immune response genes, including anti-microbial peptide (AMP) genes. The increased ROS production induces additional types of oxidative stress response genes, including Gluthione-S-transferase (Gst) genes. Protein misfolding activates the heat shock transcription factor (HSF) and mitochondrial unfolded protein response (mUPR) to induce proteotoxicity-response genes, including heat shock protein (Hsp) genes. Classes of genes up-regulated during aging are indicated with red color font.
