# Supplementary material for: Gene expression changes in response to aging compared to heat stress, oxidative stress and ionizing radiation in Drosophila melanogaster
Source: Aging (Albany NY). 2012 Nov 30;4(11):768–89. doi: 10.18632/aging.100499 (PMC3560439; doi:10.18632/aging.100499)
Supplement: Supplementary file 2 [file aging-04-768-s002.docx]

Supplemental Table S1. Gene expression changes unique to aging and each stress (sugar excluded)

Age up only

| Gene > Secondary Identifier | Gene > Symbol | Gene > Name |
| --- | --- | --- |
| CG9024 | Acp26Ab | Accessory gland-specific peptide 26Ab |
| CG1171 | Akh | Adipokinetic hormone |
| CG10142 | Ance-5 | Ance-5 |
| CG7558 | Arp66B | Actin-related protein 66B |
| CG4740 | AttC | Attacin-C |
| CG11967 | CAHbeta | Carbonic anhydrase beta |
| CG10157 | CG10157 |  |
| CG10184 | CG10184 |  |
| CG10205 | CG10205 |  |
| CG10332 | CG10332 |  |
| CG10445 | CG10445 |  |
| CG10512 | CG10512 |  |
| CG10799 | CG10799 |  |
| CG10910 | CG10910 |  |
| CG10943 | CG10943 |  |
| CG11459 | CG11459 |  |
| CG11841 | CG11841 |  |
| CG11854 | CG11854 |  |
| CG11878 | CG11878 |  |
| CG12824 | CG12824 |  |
| CG13077 | CG13077 |  |
| CG13311 | CG13311 |  |
| CG13422 | CG13422 |  |
| CG13618 | CG13618 |  |
| CG13641 | CG13641 |  |
| CG13794 | CG13794 |  |
| CG13795 | CG13795 |  |
| CG13841 | CG13841 |  |
| CG14526 | CG14526 |  |
| CG14527 | CG14527 |  |
| CG14567 | CG14567 |  |
| CG1468 | CG1468 |  |
| CG14872 | CG14872 |  |
| CG15067 | CG15067 |  |
| CG15083 | CG15083 |  |
| CG15117 | CG15117 |  |
| CG15263 | CG15263 |  |
| CG15293 | CG15293 |  |
| CG1667 | CG1667 |  |
| CG16704 | CG16704 |  |
| CG16712 | CG16712 |  |
| CG16713 | CG16713 |  |
| CG16743 | CG16743 |  |
| CG16836 | CG16836 |  |
| CG16965 | CG16965 |  |
| CG17107 | CG17107 |  |
| CG17224 | CG17224 |  |
| CG17549 | CG17549 |  |
| CG18547 | CG18547 |  |
| CG18563 | CG18563 |  |
| CG2157 | CG2157 |  |
| CG3077 | CG3077 |  |
| CG31272 | CG31272 |  |
| CG31445 | CG31445 |  |
| CG31743 | CG31743 |  |
| CG32164 | CG32164 |  |
| CG32165 | CG32165 |  |
| CG32428 | CG32428 |  |
| CG33110 | CG33110 |  |
| CG33307 | CG33307 |  |
| CG34227 | CG34227 |  |
| CG34349 | CG34349 |  |
| CG3604 | CG3604 |  |
| CG3699 | CG3699 |  |
| CG4250 | CG4250 |  |
| CG4267 | CG4267 |  |
| CG4680 | CG4680 |  |
| CG4757 | CG4757 |  |
| CG5508 | CG5508 |  |
| CG5791 | CG5791 |  |
| CG6045 | CG6045 |  |
| CG6357 | CG6357 |  |
| CG6409 | CG6409 |  |
| CG6429 | CG6429 |  |
| CG6639 | CG6639 |  |
| CG6805 | CG6805 |  |
| CG6910 | CG6910 |  |
| CG7016 | CG7016 |  |
| CG7523 | CG7523 |  |
| CG7627 | CG7627 |  |
| CG7675 | CG7675 |  |
| CG7695 | CG7695 |  |
| CG7763 | CG7763 |  |
| CG7966 | CG7966 |  |
| CG7997 | CG7997 |  |
| CG8066 | CG8066 |  |
| CG8121 | CG8121 |  |
| CG8199 | CG8199 |  |
| CG8353 | CG8353 |  |
| CG8369 | CG8369 |  |
| CG8468 | CG8468 |  |
| CG8546 | CG8546 |  |
| CG8665 | CG8665 |  |
| CG9008 | CG9008 |  |
| CG9027 | CG9027 |  |
| CG9150 | CG9150 |  |
| CG9338 | CG9338 |  |
| CG9391 | CG9391 |  |
| CG9701 | CG9701 |  |
| CG9759 | CG9759 |  |
| CG9894 | CG9894 |  |
| CG5413 | CREG | Cellular Repressor of E1A-stimulated Genes |
| CG1495 | CaMKI | Calcium/calmodulin-dependent protein kinase I |
| CG7563 | CalpA | Calpain-A |
| CG13281 | Cas | CAS/CSE1 segregation protein |
| CG1365 | CecA1 | Cecropin A1 |
| CG1367 | CecA2 | Cecropin A2 |
| CG18348 | Cpr67Fb | Cuticular protein 67Fb |
| CG4784 | Cpr72Ec | Cuticular protein 72Ec |
| CG4120 | Cyp12c1 | Cyp12c1 |
| CG2060 | Cyp4e2 | Cytochrome P450-4e2 |
| CG2397 | Cyp6a13 | Cyp6a13 |
| CG8050 | Cys | Cystatin-like |
| CG1385 | Def | Defensin |
| CG12763 | Dpt | Diptericin |
| CG10794 | DptB | Diptericin B |
| CG8357 | Drep-1 | DNA fragmentation factor-related protein 1 |
| CG3132 | Ect3 |  |
| CG6643 | Esyt2 |  |
| CG10060 | G-ialpha65A | G protein alphai subunit 65A |
| CG12030 | Gale | UDP-galactose 4'-epimerase |
| CG5288 | Galk | Galactokinase |
| CG1106 | Gel | Gelsolin |
| CG3694 | Ggamma30A | G protein gamma30A |
| CG6461 | Ggt-1 | gamma-glutamyl transpeptidase |
| CG7891 | Gie | GTPase indispensable for equal segregation of chromosomes |
| CG4233 | Got2 | Glutamate oxaloacetate transaminase 2 |
| CG18144 | Hand | Hand |
| CG33706 | IM18 | Immune induced molecule 18 |
| CG15231 | IM4 | Immune induced molecule 4 |
| CG4475 | Idgf2 | Imaginal disc growth factor 2 |
| CG8697 | Lcp2 | Larval cuticle protein 2 |
| CG6113 | Lip4 | Lipase 4 |
| CG2559 | Lsp1alpha | Larval serum protein 1 alpha |
| CG15162 | MESR3 | Misexpression suppressor of ras 3 |
| CG1483 | Map205 | Microtubule-associated protein 205 |
| CG7586 | Mcr | Macroglobulin complement-related |
| CG5475 | Mpk2 | Mpk2 |
| CG8175 | Mtk | Metchnikowin |
| CG5097 | MtnC | Metallothionein C |
| CG32656 | Muc11A | Mucin 11A |
| CG4357 | Ncc69 | sodium chloride cotransporter 69 |
| CG11797 | Obp56a | Odorant-binding protein 56a |
| CG11218 | Obp56d | Odorant-binding protein 56d |
| CG14704 | PGRP-LB | Peptidoglycan recognition protein LB |
| CG4437 | PGRP-LF | Peptidoglycan recognition protein LF |
| CG9681 | PGRP-SB1 | PGRP-SB1 |
| CG7496 | PGRP-SD | PGRP-SD |
| CG4574 | Plc21C | Phospholipase C at 21C |
| CG10078 | Prat2 | Phosphoribosylamidotransferase 2 |
| CG12323 | Prosbeta5 | Proteasome beta5 subunit |
| CG32412 | QC | Glutaminyl cyclase |
| CG3320 | Rab1 | Rab-protein 1 |
| CG9375 | Ras85D | Ras oncogene at 85D |
| CG9668 | Rh4 | Rhodopsin 4 |
| CG10071 | RpL29 | Ribosomal protein L29 |
| CG10944 | RpS6 | Ribosomal protein S6 |
| CG12070 | Sap-r | Saposin-related |
| CG18398 | Tango6 | Transport and Golgi organization 6 |
| CG7052 | TepII | Thiolester containing protein II |
| CG7068 | TepIII | Thiolester containing protein III |
| CG6281 | Timp | Tissue inhibitor of metalloproteases |
| CG30035 | Tret1-1 | Trehalose transporter 1-1 |
| CG8234 | Tret1-2 | Trehalose transporter 1-2 |
| CG4758 | Trp1 | Translocation protein 1 |
| CG6186 | Tsf1 | Transferrin 1 |
| CG10620 | Tsf2 | Transferrin 2 |
| CG11326 | Tsp | Thrombospondin |
| CG10106 | Tsp42Ee | Tetraspanin 42Ee |
| CG12845 | Tsp42Ef | Tetraspanin 42Ef |
| CG12142 | Tsp42Eg | Tetraspanin 42Eg |
| CG12840 | Tsp42El | Tetraspanin 42El |
| CG5055 | baz | bazooka |
| CG4501 | bgm | bubblegum |
| CG2813 | cold | coiled |
| CG4280 | crq | croquemort |
| CG1828 | dre4 | dre4 |
| CG10812 | dro5 | drosomycin-5 |
| CG10724 | flr | flare |
| CG9031 | ics | icarus |
| CG11312 | insc | inscuteable |
| CG1298 | kune | kune-kune |
| CG7106 | lectin-28C | lectin-28C |
| CG10080 | mahj | mahjong |
| CG14827 | mei-P22 | meiotic P22 |
| CG31839 | nimB2 | nimrod B2 |
| CG8942 | nimC1 | nimrod C1 |
| CG13848 | pinta | prolonged depolarization afterpotential (PDA) is not apparent |
| CG15678 | pirk | poor Imd response upon knock-in |
| CG6014 | rgn | regeneration |
| CG6134 | spz | spatzle |
| CG31006 | stops | slow termination of phototransduction |
| CG11909 | tobi | target of brain insulin |
| CG11186 | toy | twin of eyeless |
| CG7875 | trp | transient receptor potential |
| CG6705 | tsl | torso-like |
| CG12311 | tw | Protein O-mannosyltransferase 2 |
| CG2155 | v | vermilion |
| CG7178 | wupA | wings up A |
| CG4182 | yellow-c | yellow-c |

Age down only

| Gene > Secondary Identifier | Gene > Symbol |  |
| --- | --- | --- |
| CG32062 | A2bp1 |  |
| CG17176 | ACXA | ACXA |
| CG5983 | ACXC | ACXC |
| CG6030 | ATPsyn-d |  |
| CG4605 | Acp32CD |  |
| CG17673 | Acp70A |  |
| CG17924 | Acp95EF |  |
| CG16870 | Acyp |  |
| CG10143 | Adgf-E |  |
| CG6612 | Adk3 |  |
| CG6058 | Ald | Aldolase |
| CG17769 | And | Androcam |
| CG1683 | Ant2 |  |
| CG32031 | Argk |  |
| CG5409 | Arp53D |  |
| CG12334 | Atg8b |  |
| CG10014 | CG10014 |  |
| CG10064 | CG10064 |  |
| CG10077 | CG10077 |  |
| CG10164 | CG10164 |  |
| CG10177 | CG10177 |  |
| CG10202 | CG10202 |  |
| CG10219 | CG10219 |  |
| CG10252 | CG10252 |  |
| CG10307 | CG10307 |  |
| CG10317 | CG10317 |  |
| CG10396 | CG10396 |  |
| CG10561 | CG10561 |  |
| CG10589 | CG10589 |  |
| CG10749 | CG10749 |  |
| CG10750 | CG10750 |  |
| CG10841 | CG10841 |  |
| CG10859 | CG10859 |  |
| CG10862 | CG10862 |  |
| CG10869 | CG10869 |  |
| CG10919 | CG10919 |  |
| CG10920 | CG10920 |  |
| CG10924 | CG10924 |  |
| CG10934 | CG10934 |  |
| CG10969 | CG10969 |  |
| CG11043 | CG11043 |  |
| CG11068 | CG11068 |  |
| CG11106 | CG11106 |  |
| CG11125 | CG11125 |  |
| CG11145 | CG11145 |  |
| CG11226 | CG11226 |  |
| CG11251 | CG11251 |  |
| CG11262 | CG11262 |  |
| CG11298 | CG11298 |  |
| CG11327 | CG11327 |  |
| CG11368 | CG11368 |  |
| CG11373 | CG11373 |  |
| CG11562 | CG11562 |  |
| CG11635 | CG11635 |  |
| CG11656 | CG11656 |  |
| CG11663 | CG11663 |  |
| CG11714 | CG11714 |  |
| CG11876 | CG11876 |  |
| CG11984 | CG11984 |  |
| CG12027 | CG12027 |  |
| CG12111 | CG12111 |  |
| CG12118 | CG12118 |  |
| CG12126 | CG12126 |  |
| CG12162 | CG12162 |  |
| CG12201 | CG12201 |  |
| CG12229 | CG12229 |  |
| CG12289 | CG12289 |  |
| CG12307 | CG12307 |  |
| CG12362 | CG12362 |  |
| CG12400 | CG12400 |  |
| CG12470 | CG12470 |  |
| CG12498 | CG12498 |  |
| CG12506 | CG12506 |  |
| CG12592 | CG12592 |  |
| CG12617 | CG12617 |  |
| CG12620 | CG12620 |  |
| CG1265 | CG1265 |  |
| CG12679 | CG12679 |  |
| CG12689 | CG12689 |  |
| CG12703 | CG12703 |  |
| CG12784 | CG12784 |  |
| CG12853 | CG12853 |  |
| CG12859 | CG12859 |  |
| CG12860 | CG12860 |  |
| CG12861 | CG12861 |  |
| CG1287 | CG1287 |  |
| CG12902 | CG12902 |  |
| CG12907 | CG12907 |  |
| CG12983 | CG12983 |  |
| CG13010 | CG13010 |  |
| CG13168 | CG13168 |  |
| CG13186 | CG13186 |  |
| CG1324 | CG1324 |  |
| CG13245 | CG13245 |  |
| CG13385 | CG13385 |  |
| CG13428 | CG13428 |  |
| CG13442 | CG13442 |  |
| CG13473 | CG13473 |  |
| CG13477 | CG13477 |  |
| CG13494 | CG13494 |  |
| CG13564 | CG13564 |  |
| CG13569 | CG13569 |  |
| CG13581 | CG13581 |  |
| CG13700 | CG13700 |  |
| CG13723 | CG13723 |  |
| CG13725 | CG13725 |  |
| CG13733 | CG13733 |  |
| CG13747 | CG13747 |  |
| CG13838 | CG13838 |  |
| CG13891 | CG13891 |  |
| CG1394 | CG1394 |  |
| CG13946 | CG13946 |  |
| CG13965 | CG13965 |  |
| CG13989 | CG13989 |  |
| CG14013 | CG14013 |  |
| CG14022 | CG14022 |  |
| CG14069 | CG14069 |  |
| CG14070 | CG14070 |  |
| CG14077 | CG14077 |  |
| CG14101 | CG14101 |  |
| CG14113 | CG14113 |  |
| CG1418 | CG1418 |  |
| CG14183 | CG14183 |  |
| CG14270 | CG14270 |  |
| CG14290 | CG14290 |  |
| CG14297 | CG14297 |  |
| CG14305 | CG14305 |  |
| CG14316 | CG14316 |  |
| CG14353 | CG14353 |  |
| CG14355 | CG14355 |  |
| CG14402 | CG14402 |  |
| CG14488 | CG14488 |  |
| CG14508 | CG14508 |  |
| CG14540 | CG14540 |  |
| CG14579 | CG14579 |  |
| CG14605 | CG14605 |  |
| CG14609 | CG14609 |  |
| CG14619 | CG14619 |  |
| CG14644 | CG14644 |  |
| CG14658 | CG14658 |  |
| CG14718 | CG14718 |  |
| CG14721 | CG14721 |  |
| CG14739 | CG14739 |  |
| CG14757 | CG14757 |  |
| CG14763 | CG14763 |  |
| CG14785 | CG14785 |  |
| CG14835 | CG14835 |  |
| CG14974 | CG14974 |  |
| CG14995 | CG14995 |  |
| CG15034 | CG15034 |  |
| CG15086 | CG15086 |  |
| CG15109 | CG15109 |  |
| CG15128 | CG15128 |  |
| CG15147 | CG15147 |  |
| CG15172 | CG15172 |  |
| CG15177 | CG15177 |  |
| CG15198 | CG15198 |  |
| CG15200 | CG15200 |  |
| CG15208 | CG15208 |  |
| CG15213 | CG15213 |  |
| CG15219 | CG15219 |  |
| CG15258 | CG15258 |  |
| CG15260 | CG15260 |  |
| CG15286 | CG15286 |  |
| CG15306 | CG15306 |  |
| CG15425 | CG15425 |  |
| CG15452 | CG15452 |  |
| CG15461 | CG15461 |  |
| CG15475 | CG15475 |  |
| CG15482 | CG15482 |  |
| CG15510 | CG15510 |  |
| CG15577 | CG15577 |  |
| CG15578 | CG15578 |  |
| CG15631 | CG15631 |  |
| CG15657 | CG15657 |  |
| CG15891 | CG15891 |  |
| CG15892 | CG15892 |  |
| CG1628 | CG1628 |  |
| CG16719 | CG16719 |  |
| CG16741 | CG16741 |  |
| CG16781 | CG16781 |  |
| CG16825 | CG16825 |  |
| CG16853 | CG16853 |  |
| CG1690 | CG1690 |  |
| CG16957 | CG16957 |  |
| CG16972 | CG16972 |  |
| CG16979 | CG16979 |  |
| CG16984 | CG16984 |  |
| CG17010 | CG17010 |  |
| CG17083 | CG17083 |  |
| CG17098 | CG17098 |  |
| CG17118 | CG17118 |  |
| CG17122 | CG17122 |  |
| CG17230 | CG17230 |  |
| CG17237 | CG17237 |  |
| CG17261 | CG17261 |  |
| CG17344 | CG17344 |  |
| CG17376 | CG17376 |  |
| CG17377 | CG17377 |  |
| CG17470 | CG17470 |  |
| CG17567 | CG17567 |  |
| CG17575 | CG17575 |  |
| CG17597 | CG17597 |  |
| CG17744 | CG17744 |  |
| CG17751 | CG17751 |  |
| CG17819 | CG17819 |  |
| CG17856 | CG17856 |  |
| CG17917 | CG17917 |  |
| CG17944 | CG17944 |  |
| CG17991 | CG17991 |  |
| CG18170 | CG18170 |  |
| CG18266 | CG18266 |  |
| CG18335 | CG18335 |  |
| CG18336 | CG18336 |  |
| CG1835 | CG1835 |  |
| CG18418 | CG18418 |  |
| CG18449 | CG18449 |  |
| CG18662 | CG18662 |  |
| CG18675 | CG18675 |  |
| CG1882 | CG1882 |  |
| CG1970 | CG1970 |  |
| CG1979 | CG1979 |  |
| CG1999 | CG1999 |  |
| CG2022 | CG2022 |  |
| CG2061 | CG2061 |  |
| CG2113 | CG2113 |  |
| CG2127 | CG2127 |  |
| CG2147 | CG2147 |  |
| CG2267 | CG2267 |  |
| CG2291 | CG2291 |  |
| CG2336 | CG2336 |  |
| CG2533 | CG2533 |  |
| CG2574 | CG2574 |  |
| CG2871 | CG2871 |  |
| CG2921 | CG2921 |  |
| CG2955 | CG2955 |  |
| CG2964 | CG2964 |  |
| CG30222 | CG30222 |  |
| CG30278 | CG30278 |  |
| CG30324 | CG30324 |  |
| CG30350 | CG30350 |  |
| CG30412 | CG30412 |  |
| CG30493 | CG30493 |  |
| CG3085 | CG3085 |  |
| CG3092 | CG3092 |  |
| CG31178 | CG31178 |  |
| CG3121 | CG3121 |  |
| CG3124 | CG3124 |  |
| CG31275 | CG31275 |  |
| CG31482 | CG31482 |  |
| CG31546 | CG31546 |  |
| CG31679 | CG31679 |  |
| CG31948 | CG31948 |  |
| CG31988 | CG31988 |  |
| CG3199 | CG3199 |  |
| CG32081 | CG32081 |  |
| CG32086 | CG32086 |  |
| CG3213 | CG3213 |  |
| CG3214 | CG3214 |  |
| CG3222 | CG3222 |  |
| CG32236 | CG32236 |  |
| CG32238 | CG32238 |  |
| CG32250 | CG32250 |  |
| CG32262 | CG32262 |  |
| CG32392 | CG32392 |  |
| CG32396 | CG32396 |  |
| CG32436 | CG32436 |  |
| CG32440 | CG32440 |  |
| CG32548 | CG32548 |  |
| CG32628 | CG32628 |  |
| CG32647 | CG32647 |  |
| CG33017 | CG33017 |  |
| CG3306 | CG3306 |  |
| CG33189 | CG33189 |  |
| CG3321 | CG3321 |  |
| CG3330 | CG3330 |  |
| CG33340 | CG33340 |  |
| CG3345 | CG3345 |  |
| CG3348 | CG3348 |  |
| CG33791 | CG33791 |  |
| CG33934 | CG33934 |  |
| CG34168 | CG34168 |  |
| CG3473 | CG3473 |  |
| CG3483 | CG3483 |  |
| CG3492 | CG3492 |  |
| CG3499 | CG3499 |  |
| CG3517 | CG3517 |  |
| CG3557 | CG3557 |  |
| CG3581 | CG3581 |  |
| CG3621 | CG3621 |  |
| CG3687 | CG3687 |  |
| CG3731 | CG3731 |  |
| CG3788 | CG3788 |  |
| CG3809 | CG3809 |  |
| CG3955 | CG3955 |  |
| CG3964 | CG3964 |  |
| CG3982 | CG3982 |  |
| CG4068 | CG4068 |  |
| CG4073 | CG4073 |  |
| CG4161 | CG4161 |  |
| CG4169 | CG4169 |  |
| CG4198 | CG4198 |  |
| CG4218 | CG4218 |  |
| CG42319 | CG42319 |  |
| CG4238 | CG4238 |  |
| CG42666 | CG42666 |  |
| CG42675 | CG42675 |  |
| CG4270 | CG4270 |  |
| CG4286 | CG4286 |  |
| CG4390 | CG4390 |  |
| CG4434 | CG4434 |  |
| CG4461 | CG4461 |  |
| CG4480 | CG4480 |  |
| CG4520 | CG4520 |  |
| CG4538 | CG4538 |  |
| CG4546 | CG4546 |  |
| CG4669 | CG4669 |  |
| CG4673 | CG4673 |  |
| CG4686 | CG4686 |  |
| CG4691 | CG4691 |  |
| CG4692 | CG4692 |  |
| CG4701 | CG4701 |  |
| CG4706 | CG4706 |  |
| CG4712 | CG4712 |  |
| CG4769 | CG4769 |  |
| CG4825 | CG4825 |  |
| CG4836 | CG4836 |  |
| CG4907 | CG4907 |  |
| CG4955 | CG4955 |  |
| CG4983 | CG4983 |  |
| CG4995 | CG4995 |  |
| CG5017 | CG5017 |  |
| CG5024 | CG5024 |  |
| CG5028 | CG5028 |  |
| CG5045 | CG5045 |  |
| CG5089 | CG5089 |  |
| CG5103 | CG5103 |  |
| CG5139 | CG5139 |  |
| CG5144 | CG5144 |  |
| CG5155 | CG5155 |  |
| CG5177 | CG5177 |  |
| CG5214 | CG5214 |  |
| CG5217 | CG5217 |  |
| CG5280 | CG5280 |  |
| CG5343 | CG5343 |  |
| CG5398 | CG5398 |  |
| CG5500 | CG5500 |  |
| CG5538 | CG5538 |  |
| CG5539 | CG5539 |  |
| CG5565 | CG5565 |  |
| CG5703 | CG5703 |  |
| CG5718 | CG5718 |  |
| CG5762 | CG5762 |  |
| CG5790 | CG5790 |  |
| CG5823 | CG5823 |  |
| CG5862 | CG5862 |  |
| CG5886 | CG5886 |  |
| CG5906 | CG5906 |  |
| CG5987 | CG5987 |  |
| CG6020 | CG6020 |  |
| CG6091 | CG6091 |  |
| CG6140 | CG6140 |  |
| CG6209 | CG6209 |  |
| CG6255 | CG6255 |  |
| CG6262 | CG6262 |  |
| CG6279 | CG6279 |  |
| CG6304 | CG6304 |  |
| CG6332 | CG6332 |  |
| CG6380 | CG6380 |  |
| CG6404 | CG6404 |  |
| CG6412 | CG6412 |  |
| CG6439 | CG6439 |  |
| CG6441 | CG6441 |  |
| CG6470 | CG6470 |  |
| CG6527 | CG6527 |  |
| CG6569 | CG6569 |  |
| CG6628 | CG6628 |  |
| CG6652 | CG6652 |  |
| CG6661 | CG6661 |  |
| CG6709 | CG6709 |  |
| CG6750 | CG6750 |  |
| CG6752 | CG6752 |  |
| CG6790 | CG6790 |  |
| CG6873 | CG6873 |  |
| CG6878 | CG6878 |  |
| CG6888 | CG6888 |  |
| CG6914 | CG6914 |  |
| CG7024 | CG7024 |  |
| CG7045 | CG7045 |  |
| CG7094 | CG7094 |  |
| CG7131 | CG7131 |  |
| CG7164 | CG7164 |  |
| CG7196 | CG7196 |  |
| CG7202 | CG7202 |  |
| CG7208 | CG7208 |  |
| CG7215 | CG7215 |  |
| CG7251 | CG7251 |  |
| CG7311 | CG7311 |  |
| CG7335 | CG7335 |  |
| CG7349 | CG7349 |  |
| CG7366 | CG7366 |  |
| CG7387 | CG7387 |  |
| CG7441 | CG7441 |  |
| CG7557 | CG7557 |  |
| CG7603 | CG7603 |  |
| CG7630 | CG7630 |  |
| CG7669 | CG7669 |  |
| CG7707 | CG7707 |  |
| CG7712 | CG7712 |  |
| CG7742 | CG7742 |  |
| CG7755 | CG7755 |  |
| CG7772 | CG7772 |  |
| CG7794 | CG7794 |  |
| CG7841 | CG7841 |  |
| CG7848 | CG7848 |  |
| CG7886 | CG7886 |  |
| CG7920 | CG7920 |  |
| CG7927 | CG7927 |  |
| CG8001 | CG8001 |  |
| CG8043 | CG8043 |  |
| CG8129 | CG8129 |  |
| CG8136 | CG8136 |  |
| CG8138 | CG8138 |  |
| CG8141 | CG8141 |  |
| CG8193 | CG8193 |  |
| CG8252 | CG8252 |  |
| CG8257 | CG8257 |  |
| CG8292 | CG8292 |  |
| CG8299 | CG8299 |  |
| CG8349 | CG8349 |  |
| CG8407 | CG8407 |  |
| CG8478 | CG8478 |  |
| CG8508 | CG8508 |  |
| CG8517 | CG8517 |  |
| CG8520 | CG8520 |  |
| CG8531 | CG8531 |  |
| CG8564 | CG8564 |  |
| CG8565 | CG8565 |  |
| CG8654 | CG8654 |  |
| CG8680 | CG8680 |  |
| CG8701 | CG8701 |  |
| CG8746 | CG8746 |  |
| CG8768 | CG8768 |  |
| CG8813 | CG8813 |  |
| CG8838 | CG8838 |  |
| CG8840 | CG8840 |  |
| CG8979 | CG8979 |  |
| CG9010 | CG9010 |  |
| CG9014 | CG9014 |  |
| CG9029 | CG9029 |  |
| CG9034 | CG9034 |  |
| CG9065 | CG9065 |  |
| CG9072 | CG9072 |  |
| CG9090 | CG9090 |  |
| CG9106 | CG9106 |  |
| CG9129 | CG9129 |  |
| CG9130 | CG9130 |  |
| CG9133 | CG9133 |  |
| CG9140 | CG9140 |  |
| CG9172 | CG9172 |  |
| CG9173 | CG9173 |  |
| CG9222 | CG9222 |  |
| CG9235 | CG9235 |  |
| CG9254 | CG9254 |  |
| CG9263 | CG9263 |  |
| CG9279 | CG9279 |  |
| CG9297 | CG9297 |  |
| CG9313 | CG9313 |  |
| CG9314 | CG9314 |  |
| CG9350 | CG9350 |  |
| CG9389 | CG9389 |  |
| CG9483 | CG9483 |  |
| CG9570 | CG9570 |  |
| CG9572 | CG9572 |  |
| CG9602 | CG9602 |  |
| CG9624 | CG9624 |  |
| CG9722 | CG9722 |  |
| CG9821 | CG9821 |  |
| CG9875 | CG9875 |  |
| CG9920 | CG9920 |  |
| CG9975 | CG9975 |  |
| CG4774 | CLS |  |
| CG14437 | COQ7 | COQ7 |
| CR11386 | CR11386 |  |
| CR31781 | CR31781 |  |
| CR33222 | CR33222 |  |
| CR9284 | CR9284 |  |
| CG3725 | Ca-P60A |  |
| CG18330 | Cct2 |  |
| CG5450 | Cdlc2 |  |
| CG7313 | CheA75a |  |
| CG8914 | CkIIbeta2 |  |
| CG10664 | CoIV |  |
| CG7181 | CoVIII |  |
| CG14724 | CoVa |  |
| CG11015 | CoVb |  |
| CG6292 | CycT | Cyclin T |
| CG10245 | Cyp6a20 | Cyp6a20 |
| CG13263 | Cyt-c-d |  |
| CG11347 | DOR |  |
| CG6092 | Dak1 | Dak1 |
| CG14899 | Der-2 | Derlin-2 |
| CG7415 | DppIII |  |
| CG11591 | Dpy-30L2 |  |
| CG17654 | Eno | Enolase |
| CG42610 | Fhos |  |
| CG4435 | FucTB | FucTB |
| CG9169 | FucTD | FucTD |
| CG6128 | Fuca |  |
| CG2204 | G-oalpha47A |  |
| CG5732 | Gld2 |  |
| CG7975 | Grx-1 | Grx-1 |
| CG8938 | GstS1 |  |
| CG32849 | Hex-t2 | Hex-t2 |
| CG10377 | Hrb27C |  |
| CG10221 | Hrd3 |  |
| CG8937 | Hsc70-1 |  |
| CG7756 | Hsc70-2 |  |
| CG2830 | Hsp60B |  |
| CG7235 | Hsp60C | Hsp60C |
| CG31795 | IA-2 |  |
| CG33933 | Indy-2 |  |
| CG3219 | Klp59C | Klp59C |
| CG5231 | Las |  |
| CG3849 | Lasp | Lasp |
| CG1245 | MED27 |  |
| CG7964 | Menl-1 |  |
| CG7969 | Menl-2 |  |
| CG4479 | Mst35Ba |  |
| CG4478 | Mst35Bb |  |
| CG3354 | Mst77F |  |
| CG17946 | Mst84Da |  |
| CG17934 | Mst84Db |  |
| CG17945 | Mst84Dc |  |
| CG17935 | Mst84Dd |  |
| CG17956 | Mst87F |  |
| CG11719 | Mst98Ca |  |
| CG18396 | Mst98Cb |  |
| CG3944 | ND23 |  |
| CG6343 | ND42 |  |
| CG2286 | ND75 |  |
| CG6008 | NP15.6 | NP15.6 |
| CG15304 | Neb-cGP | Neb-cGP |
| CG2297 | Obp44a | Obp44a |
| CG18111 | Obp99a |  |
| CG33092 | P5CDh2 |  |
| CG12358 | Paip2 |  |
| CG6148 | Past1 |  |
| CG6988 | Pdi |  |
| CG4799 | Pen | Pendulin |
| CG17645 | Pglym87 | Pglym87 |
| CG42599 | Pif1A |  |
| CG4523 | Pink1 |  |
| CG12066 | Pka-C2 |  |
| CG8073 | Pmm45A |  |
| CG3691 | Pof |  |
| CG17137 | Porin2 | Porin2 |
| CG31025 | Ppi1 |  |
| CG12169 | Ppm1 | Ppm1 |
| CG5648 | Prosalpha6T |  |
| CG17302 | Prosbeta4R2 |  |
| CG7217 | Prx5 |  |
| CG7361 | RFeSP |  |
| CG14098 | Rcd7 |  |
| CG16988 | Roc1b | Roc1b |
| CG6155 | Roe1 | Roe1 |
| CG7014 | RpS5b |  |
| CG1279 | Rtnl2 | Rtnl2 |
| CG6372 | S-Lap1 |  |
| CG32064 | S-Lap4 |  |
| CG18369 | S-Lap5 |  |
| CG13340 | S-Lap7 |  |
| CG4439 | S-Lap8 |  |
| CG13164 | SIP2 |  |
| CG32380 | SMSr | SMSr |
| CG17320 | ScpX |  |
| CG1065 | Scsalpha |  |
| CG3283 | SdhB |  |
| CG9580 | Sdic1 |  |
| CG8905 | Sod2 |  |
| CG15632 | Taf12L |  |
| CG11611 | Tim13 | Tim13 |
| CG10090 | Tim17a1 | Tim17a1 |
| CG1728 | Tim8 | Tim8 |
| CG6756 | Tom70 |  |
| CG2981 | TpnC41C |  |
| CG3315 | TrxT |  |
| CG14468 | Tsp42A |  |
| CG12799 | Ubc84D |  |
| CG8284 | UbcD4 |  |
| CG4265 | Uch |  |
| CG6492 | Ucp4A | Ucp4A |
| CG33519 | Unc-89 | Unc-89 |
| CG5075 | Vha68-3 |  |
| CG14909 | VhaM9.7-d |  |
| CG6416 | Zasp66 |  |
| CG7811 | b | black |
| CG9359 | betaTub85D |  |
| CG3612 | blw |  |
| CG4760 | bol | boule |
| CG15623 | c-cup |  |
| CG3399 | capu |  |
| CG7533 | chrb | charybde |
| CG1618 | comt | comatose |
| CG33957 | cp309 | cp309 |
| CG31299 | cu | curled |
| CG11181 | cup | cup |
| CG14028 | cype | cyclope |
| CG1980 | dj | don juan |
| CG1984 | djl |  |
| CG31361 | dpr17 | dpr17 |
| CG8023 | eIF4E-3 | eIF4E-3 |
| CG10124 | eIF4E-4 | eIF4E-4 |
| CG8277 | eIF4E-5 | eIF4E-5 |
| CG6513 | endos |  |
| CG8994 | exu |  |
| CG9611 | f-cup | flyers-cup |
| CG7919 | fan | farinelli |
| CG7615 | fig |  |
| CG33756 | gdl | gonadal |
| CG33755 | gdl-ORF39 | gdl-ORF39 |
| CG15180 | glob2 | globin 2 |
| CG7340 | grsm |  |
| CG17090 | hipk |  |
| CG1338 | hydra | hydra |
| CG11025 | isopeptidase-T-3 |  |
| CG7931 | janB | janus B |
| CG1193 | kat-60L1 |  |
| CG8198 | l(1)G0136 |  |
| CG12233 | l(1)G0156 |  |
| CG2968 | l(1)G0230 |  |
| CG4094 | l(1)G0255 |  |
| CG1656 | lectin-46Ca |  |
| CG1652 | lectin-46Cb |  |
| CG4750 | loopin-1 | loopin-1 |
| CG10603 | mRpL13 |  |
| CG1320 | mRpL23 |  |
| CG14981 | mge | maggie |
| CG17492 | mib2 |  |
| CG9160 | mtacp1 |  |
| CG8362 | nmdyn-D7 | nmdyn-D7 |
| CG3620 | norpA |  |
| CG7929 | ocn | ocnus |
| CG3268 | phtf | phtf |
| CG8806 | prel | preli-like |
| CG42670 | ps | pasilla |
| CG7061 | rab3-GAP | rab3-GAP |
| CG13057 | retinin | retinin |
| CG9412 | rin | rasputin |
| CG15171 | robl37BC | robl37BC |
| CG17736 | schuy |  |
| CG5207 | scpr-A |  |
| CG17210 | scpr-B |  |
| CG5106 | scpr-C |  |
| CG13030 | sinah |  |
| CG9188 | sip2 |  |
| CG11963 | skap |  |
| CG8881 | skpB | skpB |
| CG12227 | skpF | skpF |
| CG12819 | sle |  |
| CG9131 | slmo | slowmo |
| CG9218 | sm | smooth |
| CG8489 | soti | scotti |
| CG15178 | sowi | solwind |
| CG30365 | spaw |  |
| CG18497 | spen | split ends |
| CG14735 | ssp5 |  |
| CG9001 | ste24b |  |
| CG1380 | sut4 |  |
| CG1232 | tipE |  |
| CG7958 | tna | tonalli |
| CG9660 | toc | toucan |
| CG14690 | tomboy20 | tomboy20 |
| CG8330 | tomboy40 | tomboy40 |
| CG9448 | trbd | trabid |
| CG12313 | ttm2 | tiny tim 2 |
| CG31137 | twin | twin |
| CG7363 | w-cup | world cup |
| CG13176 | wash | washout |
| CG14513 | yemalpha |  |
| CG12250 | ymp |  |
| CG42827 | CG42827 |  |
| CG42288 | CG42288 |  |
| CG34107 | CG34107 |  |

O2 up only

| Gene > Secondary Identifier | Gene > Symbol | Gene > Name | |  |
| --- | --- | --- | --- | --- |
| CG10687 | Aats-asn | Asparaginyl-tRNA synthetase | | |
| CG6335 | Aats-his | Histidyl-tRNA synthetase | | |
| CG11471 | Aats-ile | Isoleucyl-tRNA synthetase | | |
| CG9735 | Aats-trp | Tryptophanyl-tRNA synthetase | | |
| CG3017 | Alas | Aminolevulinate synthase | | |
| CG1021 | CG1021 |  |  |  |
| CG11760 | CG11760 |  |  |  |
| CG11897 | CG11897 |  |  |  |
| CG12171 | CG12171 |  |  |  |
| CG12262 | CG12262 |  |  |  |
| CG12428 | CG12428 |  |  |  |
| CG12784 | CG12784 |  |  |  |
| CG13315 | CG13315 |  |  |  |
| CG13659 | CG13659 |  |  |  |
| CG13838 | CG13838 |  |  |  |
| CG14782 | CG14782 |  |  |  |
| CG14974 | CG14974 |  |  |  |
| CG15012 | CG15012 |  |  |  |
| CG15661 | CG15661 |  |  |  |
| CG15818 | CG15818 |  |  |  |
| CG18522 | CG18522 |  |  |  |
| CG1890 | CG1890 |  |  |  |
| CG1942 | CG1942 |  |  |  |
| CG30152 | CG30152 |  |  |  |
| CG31549 | CG31549 |  |  |  |
| CG33282 | CG33282 |  |  |  |
| CG3376 | CG3376 |  |  |  |
| CG3397 | CG3397 |  |  |  |
| CG4726 | CG4726 |  |  |  |
| CG5323 | CG5323 |  |  |  |
| CG5646 | CG5646 |  |  |  |
| CG5853 | CG5853 |  |  |  |
| CG6115 | CG6115 |  |  |  |
| CG6234 | CG6234 |  |  |  |
| CG6459 | CG6459 |  |  |  |
| CG7224 | CG7224 |  |  |  |
| CG8249 | CG8249 |  |  |  |
| CG8520 | CG8520 |  |  |  |
| CG8974 | CG8974 |  |  |  |
| CG9009 | CG9009 |  |  |  |
| CG9065 | CG9065 |  |  |  |
| CG5203 | CHIP | CHIP |  |  |
| CG8203 | Cdk5 | Cyclin-dependent kinase 5 | | |
| CG7313 | CheA75a | Chemosensory protein A 75a | | |
| CG18559 | Cyp309a2 | Cyp309a2 |  |  |
| CG8345 | Cyp6w1 | Cyp6w1 |  |  |
| CG17035 | GXIVsPLA2 | GXIVsPLA2 | |  |
| CG30359 | Mal-A5 | Maltase A5 | |  |
| CG14709 | Mrp4 | Multidrug resistance protein 4 ortholog | | |
| CG5330 | Nap1 | Nucleosome assembly protein 1 | | |
| CG11661 | Nc73EF | Neural conserved at 73EF | | |
| CG30443 | Opbp | Optix-binding protein | | |
| CG4909 | POSH | Plenty of SH3s | |  |
| CG1554 | RpII215 | RNA polymerase II 215kD subunit | | |
| CG12775 | RpL21 | Ribosomal protein L21 | | |
| CG6764 | RpL24-like | Ribosomal protein L24-like | | |
| CG4918 | RpLP2 | Ribosomal protein LP2 | | |
| CG8396 | Ssb-c31a | Single stranded-binding protein c31A | | |
| CG2981 | TpnC41C | Troponin C at 41C | |  |
| CG2257 | Ubc-E2H | Ubc-E2H |  |  |
| CG5798 | Ubpy | UBPY ortholog | |  |
| CG6815 | bor | belphegor |  |  |
| CG31299 | cu | curled |  |  |
| CG6494 | h | hairy |  |  |
| CG4779 | hgo | homogentisate 1,2-dioxygenase | | |
| CG6718 | iPLA2-VIA | calcium-independent phospholipase A2 VIA | | |
| CG17330 | jhamt | juvenile hormone acid methyltransferase | | |
| CG8198 | l(1)G0136 | lethal (1) G0136 | |  |
| CG10691 | l(2)37Cc | lethal (2) 37Cc | |  |
| CG6302 | l(3)01239 | lethal (3) 01239 | |  |
| CG9165 | l(3)02640 | lethal (3) 02640 | |  |
| CG17227 | lig3 | DNA ligase III | |  |
| CG14648 | lost | lost |  |  |
| CG1692 | mal | maroon-like | |  |
| CG7936 | mex1 | midgut expression 1 | |  |
| CG8153 | mus210 | mutagen-sensitive 210 | | |
| CG6501 | ns2 | nucleostemin 2 | |  |
| CG13057 | retinin | retinin |  |  |
| CG10751 | robl | roadblock |  |  |
| CG14029 | vri | vrille |  |  |
| CG4600 | yip2 | yippee interacting protein 2 | | |
| CG42827 | CG42827 |  |  | note: this gene is found in the age down list as well. |

O2 down only

| Gene > Secondary Identifier | Gene > Symbol | Gene > Name |
| --- | --- | --- |
| CG8626 | Acp53C14a | Acp53C14a |
| CG15616 | Acp53C14b | Acp53C14b |
| CG8622 | Acp53Ea | Accessory gland-specific peptide 53Ea |
| CG3971 | Baldspot | Baldspot |
| CG10096 | CG10096 |  |
| CG10097 | CG10097 |  |
| CG10170 | CG10170 |  |
| CG11037 | CG11037 |  |
| CG11391 | CG11391 |  |
| CG11590 | CG11590 |  |
| CG11878 | CG11878 |  |
| CG11961 | CG11961 |  |
| CG1246 | CG1246 |  |
| CG13283 | CG13283 |  |
| CG14153 | CG14153 |  |
| CG14302 | CG14302 |  |
| CG14500 | CG14500 |  |
| CG14528 | CG14528 |  |
| CG14879 | CG14879 |  |
| CG15116 | CG15116 |  |
| CG15126 | CG15126 |  |
| CG15199 | CG15199 |  |
| CG15202 | CG15202 |  |
| CG15408 | CG15408 |  |
| CG15649 | CG15649 |  |
| CG1681 | CG1681 |  |
| CG17323 | CG17323 |  |
| CG17637 | CG17637 |  |
| CG17839 | CG17839 |  |
| CG18258 | CG18258 |  |
| CG2233 | CG2233 |  |
| CG2254 | CG2254 |  |
| CG2862 | CG2862 |  |
| CG31659 | CG31659 |  |
| CG3168 | CG3168 |  |
| CG32521 | CG32521 |  |
| CG33080 | CG33080 |  |
| CG34324 | CG34324 |  |
| CG34330 | CG34330 |  |
| CG3857 | CG3857 |  |
| CG3868 | CG3868 |  |
| CG3906 | CG3906 |  |
| CG4288 | CG4288 |  |
| CG4666 | CG4666 |  |
| CG5326 | CG5326 |  |
| CG5758 | CG5758 |  |
| CG5767 | CG5767 |  |
| CG5773 | CG5773 |  |
| CG6012 | CG6012 |  |
| CG6071 | CG6071 |  |
| CG6950 | CG6950 |  |
| CG7025 | CG7025 |  |
| CG7300 | CG7300 |  |
| CG7720 | CG7720 |  |
| CG7724 | CG7724 |  |
| CG7992 | CG7992 |  |
| CG9396 | CG9396 |  |
| CG9427 | CG9427 |  |
| CG9436 | CG9436 |  |
| CG9449 | CG9449 |  |
| CG9701 | CG9701 |  |
| CG9911 | CG9911 |  |
| CG10531 | Cht9 | Cht9 |
| CG3244 | Clect27 | C-type lectin 27kD |
| CG4784 | Cpr72Ec | Cuticular protein 72Ec |
| CG13977 | Cyp6a18 | Cyp6a18 |
| CG8983 | ERp60 | ERp60 |
| CG9847 | Fkbp13 | Fkbp13 |
| CG1780 | Idgf4 | Imaginal disc growth factor 4 |
| CG3953 | Invadolysin | Invadolysin |
| CG8867 | Jon25Bi | Jonah 25Bi |
| CG6806 | Lsp2 | Larval serum protein 2 |
| CG1742 | Mgstl | Microsomal glutathione S-transferase-like |
| CG7874 | Mur18B | Mucin related 18B |
| CG11051 | Nplp2 | Neuropeptide-like precursor 2 |
| CG13873 | Obp56g | Odorant-binding protein 56g |
| CG9022 | Ost48 | Oligosaccharyltransferase 48kD subunit |
| CG9720 | PH4alphaNE2 | prolyl-4-hydroxylase-alpha NE2 |
| CG9358 | Phk-3 | Pherokine 3 |
| CG5826 | Prx3 | Peroxiredoxin 3 |
| CG5279 | Rh5 | Rhodopsin 5 |
| CG33113 | Rtnl1 | Rtnl1 |
| CG9539 | Sec61alpha | Sec61alpha |
| CG11500 | Spase12 | Spase 12-subunit |
| CG1751 | Spase25 | Spase 25-subunit |
| CG9456 | Spn1 | Serine protease inhibitor 1 |
| CG9334 | Spn3 | Serine protease inhibitor 3 |
| CG6717 | Spn7 | Serine protease inhibitor 7 |
| CG6289 | Spn77Bc | Serpin 77Bc |
| CG10363 | TepIV | Thiolester containing protein IV |
| CG6186 | Tsf1 | Transferrin 1 |
| CG9494 | Tsp29Fa | Tetraspanin 29Fa |
| CG9481 | Ugt37b1 | UDP-glycosyltransferase 37b1 |
| CG18617 | Vha100-2 | Vacuolar H[+] ATPase subunit 100-2 |
| CG3994 | ZnT35C |  |
| CG2139 | aralar1 | aralar1 |
| CG3401 | betaTub60D | beta-Tubulin at 60D |
| CG3504 | inaD | inactivation no afterpotential D |
| CG12794 | lcs | la costa |
| CG3359 | mfas | midline fasciclin |
| CG14560 | msopa | male-specific opa containing gene |
| CG3966 | ninaA | neither inactivation nor afterpotential A |
| CG5125 | ninaC | neither inactivation nor afterpotential C |
| CG4550 | ninaE | neither inactivation nor afterpotential E |
| CG12548 | nompB | no mechanoreceptor potential B |
| CG11142 | obst-E | obstructor-E |
| CG3019 | su(w[a]) | suppressor of white-apricot |
| CG18000 | sw | short wing |
| CG12120 | t | tan |
| CG2358 | twr | twisted bristles roughened eye |
| CG4827 | veil | veil |
| CG7225 | wbl | windbeutel |
| CG34130 | CG34130 |  |

H2O2 up only

| Gene > Secondary Identifier | Gene > Symbol | Gene > Name |
| --- | --- | --- |
| CG42573 | 2mit |  |
| CG6671 | AGO1 | Argonaute-1 |
| CG4027 | Act5C | Actin 5C |
| CG11062 | Actbeta | Activin-beta |
| CG1451 | Apc | APC-like |
| CG14444 | Apc7 | Anaphase promoting complex 7 |
| CG17252 | BCL7-like | BCL7-like |
| CG11848 | Bili | Band4.1 inhibitor LRP interactor |
| CG11177 | BthD | BthD selenoprotein |
| CG1435 | CBP | sarcoplasmic calcium-binding protein |
| CG10301 | CG10301 |  |
| CG10376 | CG10376 |  |
| CG10444 | CG10444 |  |
| CG10466 | CG10466 |  |
| CG10588 | CG10588 |  |
| CG10700 | CG10700 |  |
| CG10814 | CG10814 |  |
| CG1090 | CG1090 |  |
| CG1129 | CG1129 |  |
| CG11617 | CG11617 |  |
| CG11671 | CG11671 |  |
| CG11815 | CG11815 |  |
| CG11859 | CG11859 |  |
| CG11873 | CG11873 |  |
| CG11883 | CG11883 |  |
| CG12128 | CG12128 |  |
| CG12179 | CG12179 |  |
| CG12214 | CG12214 |  |
| CG12402 | CG12402 |  |
| CG12483 | CG12483 |  |
| CG12506 | CG12506 |  |
| CG12520 | CG12520 |  |
| CG12546 | CG12546 |  |
| CG12702 | CG12702 |  |
| CG12713 | CG12713 |  |
| CG12728 | CG12728 |  |
| CG12851 | CG12851 |  |
| CG13075 | CG13075 |  |
| CG13436 | CG13436 |  |
| CG13604 | CG13604 |  |
| CG13624 | CG13624 |  |
| CG13653 | CG13653 |  |
| CG13704 | CG13704 |  |
| CG13946 | CG13946 |  |
| CG14022 | CG14022 |  |
| CG14053 | CG14053 |  |
| CG14088 | CG14088 |  |
| CG14258 | CG14258 |  |
| CG14416 | CG14416 |  |
| CG1463 | CG1463 |  |
| CG14693 | CG14693 |  |
| CG14841 | CG14841 |  |
| CG14957 | CG14957 |  |
| CG15024 | CG15024 |  |
| CG15042 | CG15042 |  |
| CG15144 | CG15144 |  |
| CG15249 | CG15249 |  |
| CG15423 | CG15423 |  |
| CG15445 | CG15445 |  |
| CG15739 | CG15739 |  |
| CG15817 | CG15817 |  |
| CG16721 | CG16721 |  |
| CG1678 | CG1678 |  |
| CG16956 | CG16956 |  |
| CG1698 | CG1698 |  |
| CG17177 | CG17177 |  |
| CG17265 | CG17265 |  |
| CG17760 | CG17760 |  |
| CG17780 | CG17780 |  |
| CG17786 | CG17786 |  |
| CG17801 | CG17801 |  |
| CG17834 | CG17834 |  |
| CG17994 | CG17994 |  |
| CG18213 | CG18213 |  |
| CG18420 | CG18420 |  |
| CG18586 | CG18586 |  |
| CG18643 | CG18643 |  |
| CG18659 | CG18659 |  |
| CG1894 | CG1894 |  |
| CG2034 | CG2034 |  |
| CG2678 | CG2678 |  |
| CG30007 | CG30007 |  |
| CG30020 | CG30020 |  |
| CG30095 | CG30095 |  |
| CG30101 | CG30101 |  |
| CG30259 | CG30259 |  |
| CG30485 | CG30485 |  |
| CG3061 | CG3061 |  |
| CG31004 | CG31004 |  |
| CG31248 | CG31248 |  |
| CG31291 | CG31291 |  |
| CG31345 | CG31345 |  |
| CG31368 | CG31368 |  |
| CG3168 | CG3168 |  |
| CG31729 | CG31729 |  |
| CG32091 | CG32091 |  |
| CG32109 | CG32109 |  |
| CG32302 | CG32302 |  |
| CG32365 | CG32365 |  |
| CG32512 | CG32512 |  |
| CG3253 | CG3253 |  |
| CG32541 | CG32541 |  |
| CG32683 | CG32683 |  |
| CG33253 | CG33253 |  |
| CG33275 | CG33275 |  |
| CG3349 | CG3349 |  |
| CG33543 | CG33543 |  |
| CG33691 | CG33691 |  |
| CG33692 | CG33692 |  |
| CG34126 | CG34126 |  |
| CG34386 | CG34386 |  |
| CG3726 | CG3726 |  |
| CG4004 | CG4004 |  |
| CG4066 | CG4066 |  |
| CG4213 | CG4213 |  |
| CG42232 | CG42232 |  |
| CG42342 | CG42342 |  |
| CG42343 | CG42343 |  |
| CG42673 | CG42673 |  |
| CG4367 | CG4367 |  |
| CG4650 | CG4650 |  |
| CG4658 | CG4658 |  |
| CG4683 | CG4683 |  |
| CG4733 | CG4733 |  |
| CG4768 | CG4768 |  |
| CG4953 | CG4953 |  |
| CG5078 | CG5078 |  |
| CG5550 | CG5550 |  |
| CG5568 | CG5568 |  |
| CG5630 | CG5630 |  |
| CG5807 | CG5807 |  |
| CG6040 | CG6040 |  |
| CG6175 | CG6175 |  |
| CG6277 | CG6277 |  |
| CG6398 | CG6398 |  |
| CG6498 | CG6498 |  |
| CG6569 | CG6569 |  |
| CG6688 | CG6688 |  |
| CG6788 | CG6788 |  |
| CG6808 | CG6808 |  |
| CG6954 | CG6954 |  |
| CG6981 | CG6981 |  |
| CG7053 | CG7053 |  |
| CG7071 | CG7071 |  |
| CG7083 | CG7083 |  |
| CG7139 | CG7139 |  |
| CG7180 | CG7180 |  |
| CG7252 | CG7252 |  |
| CG7288 | CG7288 |  |
| CG7362 | CG7362 |  |
| CG7370 | CG7370 |  |
| CG7372 | CG7372 |  |
| CG7409 | CG7409 |  |
| CG7824 | CG7824 |  |
| CG7922 | CG7922 |  |
| CG8089 | CG8089 |  |
| CG8117 | CG8117 |  |
| CG8247 | CG8247 |  |
| CG8552 | CG8552 |  |
| CG9003 | CG9003 |  |
| CG9005 | CG9005 |  |
| CG9068 | CG9068 |  |
| CG9236 | CG9236 |  |
| CG9317 | CG9317 |  |
| CG9363 | CG9363 |  |
| CG9500 | CG9500 |  |
| CG9628 | CG9628 |  |
| CG9801 | CG9801 |  |
| CR32496 | CR32496 |  |
| CR9162 | CR9162 |  |
| CG5603 | CYLD | cylindromatosis |
| CG13664 | Cad96Cb | Cad96Cb |
| CG42332 | Camta | Calmodulin-binding transcription activator |
| CG2360 | Ccp84Aa | Ccp84Aa |
| CG1252 | Ccp84Ab | Ccp84Ab |
| CG31536 | Cdep | Cdep |
| CG31258 | Cenp-C | Cenp-C |
| CG17081 | Cep135 |  |
| CG14996 | Chd64 | Chd64 |
| CG3986 | Cht4 | Chitinase 4 |
| CG42701 | Cng | Cyclic-nucleotide-gated ion channel protein |
| CG13934 | Cpr62Ba | Cuticular protein 62Ba |
| CG18349 | Cpr67Fa2 | Cuticular protein 67Fa2 |
| CG5940 | CycA | Cyclin A |
| CG3938 | CycE | Cyclin E |
| CG15807 | Cyp313a5 | Cyp313a5 |
| CG8540 | Cyp316a1 | Cyp316a1 |
| CG1786 | Cyp318a1 | Cyp318a1 |
| CG12759 | Dbp45A | DEAD box protein 45A |
| CG6794 | Dif | Dorsal-related immunity factor |
| CG8529 | Dyb | Dystrobrevin-like |
| CG7776 | E(Pc) | Enhancer of Polycomb |
| CG9930 | E5 | E5 |
| CG7325 | Eig71Ek | Eig71Ek |
| CG15855 | Eip63F-1 | Ecdysone-induced protein 63F 1 |
| CG32180 | Eip74EF | Ecdysone-induced protein 74EF |
| CG11801 | Elo68beta | Elongase 68beta |
| CG15573 | Femcoat | Femcoat |
| CG34368 | Fili | Fish-lips |
| CG14991 | Fit1 | Fermitin 1 |
| CG5907 | Frq2 | Frequenin 2 |
| CG33466 | Fs | Follistatin |
| CG2995 | G9a | G9a |
| CG10287 | Gasp | Gasp |
| CG15396 | Gr23a | Gustatory receptor 23a |
| CG31748 | Gr36c | Gustatory receptor 36c |
| CG32256 | Gr64c | Gustatory receptor 64c |
| CG11607 | H2.0 | Homeodomain protein 2.0 |
| CG8120 | HP1e | HP1e |
| CG1745 | HP5 | Heterochromatin protein 5 |
| CG12367 | Hen1 |  |
| CG4026 | IP3K1 | Inositol 1,4,5-triphosphate kinase 1 |
| CG7788 | Ice | Ice |
| CG34143 | Ir10a | Ionotropic receptor 10a |
| CG17382 | Ir94h | Ionotropic receptor 94h |
| CG1063 | Itp-r83A | Inositol 1,4,5,-tris-phosphate receptor |
| CG1848 | LIMK1 | LIM-kinase1 |
| CG10255 | Lap1 | Lap1 |
| CG10443 | Lar | Leukocyte-antigen-related-like |
| CG10533 | Lcp65Af | Lcp65Af |
| CG8440 | Lis-1 | Lissencephaly-1 |
| CG5959 | MCO3 | multicopper oxidase 3 |
| CG6896 | MYPT-75D | MYPT-75D |
| CG12399 | Mad | Mothers against dpp |
| CG9241 | Mcm10 | Sensitized chromosome inheritance modifier 19 |
| CG11100 | Mes2 | Mes2 |
| CG43369 | Mitf | Mitf |
| CG32774 | Muc4B | Mucin 4B |
| CG11172 | NFAT | NFAT homolog |
| CG31216 | Naam | Nicotinamide amidase |
| CG5994 | Nelf-E | Negative elongation factor E |
| CG4058 | Nep4 | Neprilysin 4 |
| CG5811 | NepYr | Neuropeptide Y receptor-like |
| CG18657 | NetA | Netrin-A |
| CG6417 | Oatp33Eb | Organic anion transporting polypeptide 33Eb |
| CG7485 | Oct-TyrR | Octopamine-Tyramine receptor |
| CG16960 | Or33a | Odorant receptor 33a |
| CG16961 | Or33b | Odorant receptor 33b |
| CG1978 | Or45a | Odorant receptor 45a |
| CG12501 | Or56a | Odorant receptor 56a |
| CG17911 | Or85c | Odorant receptor 85c |
| CG17916 | Or92a | Odorant receptor 92a |
| CG5540 | Or98a | Odorant receptor 98a |
| CG2163 | Pabp2 | Pabp2 |
| CG5208 | Patr-1 | Protein associated with topo II related - 1 |
| CG34341 | Pde11 | Phosphodiesterase 11 |
| CG7001 | Pk17E | Protein kinase-like 17E |
| CG8169 | Pms2 | Pms2 |
| CG3691 | Pof | Painting of fourth |
| CG4851 | Ppt2 | Palmitoyl-protein Thioesterase 2 |
| CG17302 | Prosbeta4R2 | Proteasome beta4R2 subunit |
| CG9181 | Ptp61F | Protein tyrosine phosphatase 61F |
| CG7103 | Pvf1 | PDGF- and VEGF-related factor 1 |
| CG5053 | RASSF8 |  |
| CG8085 | RN-tre | tre oncogene-related protein |
| CG12190 | RYBP | Ring and YY1 Binding Protein |
| CG7062 | Rab-RP3 | Rab-related protein 3 |
| CG5771 | Rab11 | Rab-protein 11 |
| CG32678 | Rab9D | Rab GTPase 9D |
| CG9807 | Rab9Db | Rab GTPase 9Db |
| CG32671 | Rab9Fa | Rab GTPase 9Fa |
| CG4879 | RecQ5 | homolog of RecQ |
| CG4937 | RhoGAP15B | RhoGAP15B |
| CG11274 | SRm160 | SRm160 |
| CG11299 | Sesn | Sestrin |
| CG8582 | Sh3beta | Sh3beta |
| CG10155 | Spred | Sprouty-related protein with EVH-1 domain |
| CG4931 | Sra-1 | specifically Rac1-associated protein 1 |
| CG4852 | Sras | severas |
| CG6521 | Stam | Signal transducing adaptor molecule |
| CG12864 | Su(var)2-HP2 | Su(var)2-HP2 |
| CG7869 | SuUR | Suppressor of Under-Replication |
| CG2381 | Syt7 | Syt7 |
| CG11278 | Syx13 | Syntaxin 13 |
| CG14503 | Tango8 | Transport and Golgi organization 8 |
| CG11415 | Tsp2A | Tetraspanin 2A |
| CG5476 | TwdlN | TweedleN |
| CR14638 | TwdlU | TweedleU |
| CG32744 | Ubi-p5E | Ubiquitin-5E |
| CG3981 | Unc-76 | Unc-76 |
| CG6842 | Vps4 | Vacuolar protein sorting 4 |
| CG7670 | WRNexo | WRN exonuclease |
| CG1989 | Yippee | Yippee |
| CG11937 | amn | amnesiac |
| CG4531 | aos | argos |
| CG13402 | betaNACtes1 | |
| CG4722 | bib | big brain |
| CG11491 | br | broad |
| CG5264 | btn | buttonless |
| CG4249 | c(2)M | crossover suppressor on 2 of Manheim |
| CG17604 | c(3)G | crossover suppressor on 3 of Gowen |
| CG6577 | can | cannonball |
| CG6027 | cdi | center divider |
| CG8681 | clumsy | clumsy |
| CG6392 | cmet | CENP-meta |
| CG43286 | cnc | cap-n-collar |
| CG33957 | cp309 | cp309 |
| CG31243 | cpo | couch potato |
| CG12410 | cv | crossveinless |
| CG34389 | cv-c | crossveinless c |
| CG32498 | dnc | dunce |
| CG14226 | dome | domeless |
| CG9885 | dpp | decapentaplegic |
| CG7863 | dream | dream |
| CG32279 | dro2 | drosomycin-2 |
| CG32474 | dys | dysfusion |
| CG6611 | ect | ectodermal |
| CG7383 | eg | eagle |
| CG4114 | ex | expanded |
| CG7693 | fray | frayed |
| CG4790 | fs(1)M3 | female sterile (1) M3 |
| CG4059 | ftz-f1 | ftz transcription factor 1 |
| CG1500 | fw | furrowed |
| CG12245 | gcm | glial cells missing |
| CG2679 | gol | goliath |
| CG7897 | gp210 | gp210 |
| CG9656 | grn | grain |
| CG31043 | gukh | GUK-holder |
| CG31753 | ham | hamlet |
| CG17090 | hipk | homeodomain interacting protein kinase |
| CG3696 | kis | kismet |
| CG15707 | krimp | krimper |
| CG32597 | l(1)G0469 | lethal (1) G0469 |
| CG32210 | l(3)76BDr |  |
| CG2684 | lds | lodestar |
| CG3697 | mei-9 | meiotic 9 |
| CG12063 | mey | morpheyus |
| CG42611 | mgl | Megalin |
| CG5360 | mi | minus |
| CG16992 | mthl6 | methuselah-like 6 |
| CG1560 | mys | myospheroid |
| CG10250 | nau | nautilus |
| CG13906 | nerfin-1 | nervous fingers 1 |
| CG11988 | neur | neuralized |
| CG33119 | nimB1 | nimrod B1 |
| CG4211 | nonA | no on or off transient A |
| CG5637 | nos | nanos |
| CG1133 | opa | odd paired |
| CG43113 | orb2 |  |
| CG10901 | osk | oskar |
| CG8967 | otk | off-track |
| CG6824 | ovo | ovo |
| CG8201 | par-1 | par-1 |
| CG4445 | pgant3 | polypeptide GalNAc transferase 3 |
| CG17743 | pho | pleiohomeotic |
| CG9614 | pip | pipe |
| CG3978 | pnr | pannier |
| CG33349 | ppk25 | pickpocket 25 |
| CG43140 | pyd | polychaetoid |
| CG18572 | r | rudimentary |
| CG6831 | rhea | rhea |
| CG10683 | rhi | rhino |
| CG7230 | rib | ribbon |
| CG7036 | rno | rhinoceros |
| CG1058 | rpk | ripped pocket |
| CG4922 | sala | spalt-adjacent |
| CG8095 | scb | scab |
| CG5227 | sdk | sidekick |
| CG5341 | sec6 | sec6 |
| CG13209 | sha | shavenoid |
| CG8055 | shrb | shrub |
| CG31062 | side | sidestep |
| CG5263 | smg | smaug |
| CG33141 | sns | sticks and stones |
| CG10334 | spi | spitz |
| CG10076 | spir | spire |
| CG7847 | sr | stripe |
| CG11489 | srpk79D | serine-arginine protein kinase at 79D |
| CG11628 | step | steppke |
| CG3429 | swa | swallow |
| CG9765 | tacc | transforming acidic coiled-coil protein |
| CG7659 | tap | target of Poxn |
| CG8961 | tef | teflon |
| CG1378 | tll | tailless |
| CG5619 | trk | trunk |
| CG10619 | tup | tailup |
| CG6235 | tws | twins |
| CG32381 | unc-13-4A | unc-13-4A |
| CG8166 | unc-5 | unc-5 |
| CG4620 | unk | unkempt |
| CG5988 | upd2 | unpaired 2 |
| CG9326 | vari | varicose |
| CG43081 | vas | vasa |
| CG42677 | wb | wing blister |
| CG31732 | yuri | yuri gagarin |
| CG1449 | zfh2 | Zn finger homeodomain 2 |
| CG42818 | CG42818 |  |
| CG42666 | CG42666 |  |
| CG43155 | CG43155 |  |

H2O2 down only

| Gene > Secondary Identifier | Gene > Symbol | Gene > Name |
| --- | --- | --- |
| CG8947 | 26-29-p | 26-29kD-proteinase |
| CG10001 | AR-2 | Allatostatin Receptor 2 |
| CG31322 | Aats-met | Methionyl-tRNA synthetase |
| CG9749 | Abi | Abelson interacting protein |
| CG10067 | Act57B | Actin 57B |
| CG1361 | Anp | Andropin |
| CG11419 | Apc10 | Anaphase promoting complex subunit 10 |
| CG10851 | B52 | B52 |
| CG1845 | Br140 |  |
| CG1911 | CAP-D2 | CAP-D2 condensin subunit |
| CG10165 | CG10165 |  |
| CG10474 | CG10474 |  |
| CG10834 | CG10834 |  |
| CG10858 | CG10858 |  |
| CG10877 | CG10877 |  |
| CG11007 | CG11007 |  |
| CG11151 | CG11151 |  |
| CG11162 | CG11162 |  |
| CG11163 | CG11163 |  |
| CG11227 | CG11227 |  |
| CG1124 | CG1124 |  |
| CG11267 | CG11267 |  |
| CG11334 | CG11334 |  |
| CG1142 | CG1142 |  |
| CG11504 | CG11504 |  |
| CG11693 | CG11693 |  |
| CG11779 | CG11779 |  |
| CG11835 | CG11835 |  |
| CG11837 | CG11837 |  |
| CG1240 | CG1240 |  |
| CG12419 | CG12419 |  |
| CG12848 | CG12848 |  |
| CG12951 | CG12951 |  |
| CG12963 | CG12963 |  |
| CG13018 | CG13018 |  |
| CG13024 | CG13024 |  |
| CG13059 | CG13059 |  |
| CG13060 | CG13060 |  |
| CG13064 | CG13064 |  |
| CG13073 | CG13073 |  |
| CG13081 | CG13081 |  |
| CG13082 | CG13082 |  |
| CG13084 | CG13084 |  |
| CG13360 | CG13360 |  |
| CG13566 | CG13566 |  |
| CG13674 | CG13674 |  |
| CG13912 | CG13912 |  |
| CG13919 | CG13919 |  |
| CG14275 | CG14275 |  |
| CG14377 | CG14377 |  |
| CG14568 | CG14568 |  |
| CG1458 | CG1458 |  |
| CG14607 | CG14607 |  |
| CG14798 | CG14798 |  |
| CG1492 | CG1492 |  |
| CG14937 | CG14937 |  |
| CG14945 | CG14945 |  |
| CG15120 | CG15120 |  |
| CG15221 | CG15221 |  |
| CG15450 | CG15450 |  |
| CG15506 | CG15506 |  |
| CG15515 | CG15515 |  |
| CG15547 | CG15547 |  |
| CG15555 | CG15555 |  |
| CG15599 | CG15599 |  |
| CG15887 | CG15887 |  |
| CG1640 | CG1640 |  |
| CG16713 | CG16713 |  |
| CG16884 | CG16884 |  |
| CG16978 | CG16978 |  |
| CG17107 | CG17107 |  |
| CG17929 | CG17929 |  |
| CG17931 | CG17931 |  |
| CG18519 | CG18519 |  |
| CG18547 | CG18547 |  |
| CG1998 | CG1998 |  |
| CG2493 | CG2493 |  |
| CG2915 | CG2915 |  |
| CG30194 | CG30194 |  |
| CG30502 | CG30502 |  |
| CG3078 | CG3078 |  |
| CG31189 | CG31189 |  |
| CG31371 | CG31371 |  |
| CG31619 | CG31619 |  |
| CG31646 | CG31646 |  |
| CG31886 | CG31886 |  |
| CG3198 | CG3198 |  |
| CG3215 | CG3215 |  |
| CG32269 | CG32269 |  |
| CG32270 | CG32270 |  |
| CG32512 | CG32512 |  |
| CG32603 | CG32603 |  |
| CG3267 | CG3267 |  |
| CG32732 | CG32732 |  |
| CG32983 | CG32983 |  |
| CG33214 | CG33214 |  |
| CG33253 | CG33253 |  |
| CG34127 | CG34127 |  |
| CG3420 | CG3420 |  |
| CG34227 | CG34227 |  |
| CG34353 | CG34353 |  |
| CG34409 | CG34409 |  |
| CG3630 | CG3630 |  |
| CG3792 | CG3792 |  |
| CG3984 | CG3984 |  |
| CG4022 | CG4022 |  |
| CG42593 | CG42593 |  |
| CG4266 | CG4266 |  |
| CG4313 | CG4313 |  |
| CG4338 | CG4338 |  |
| CG4382 | CG4382 |  |
| CG4592 | CG4592 |  |
| CG4607 | CG4607 |  |
| CG4914 | CG4914 |  |
| CG5044 | CG5044 |  |
| CG5112 | CG5112 |  |
| CG5167 | CG5167 |  |
| CG5171 | CG5171 |  |
| CG5446 | CG5446 |  |
| CG5484 | CG5484 |  |
| CG5532 | CG5532 |  |
| CG5697 | CG5697 |  |
| CG5746 | CG5746 |  |
| CG5835 | CG5835 |  |
| CG5885 | CG5885 |  |
| CG5946 | CG5946 |  |
| CG6055 | CG6055 |  |
| CG6178 | CG6178 |  |
| CG6370 | CG6370 |  |
| CG6432 | CG6432 |  |
| CG6459 | CG6459 |  |
| CG6465 | CG6465 |  |
| CG6659 | CG6659 |  |
| CG6674 | CG6674 |  |
| CG7080 | CG7080 |  |
| CG7231 | CG7231 |  |
| CG7272 | CG7272 |  |
| CG7329 | CG7329 |  |
| CG7365 | CG7365 |  |
| CG7414 | CG7414 |  |
| CG7442 | CG7442 |  |
| CG7458 | CG7458 |  |
| CG7778 | CG7778 |  |
| CG7829 | CG7829 |  |
| CG7910 | CG7910 |  |
| CG7943 | CG7943 |  |
| CG8036 | CG8036 |  |
| CG8051 | CG8051 |  |
| CG8067 | CG8067 |  |
| CG8083 | CG8083 |  |
| CG8311 | CG8311 |  |
| CG8359 | CG8359 |  |
| CG8446 | CG8446 |  |
| CG8449 | CG8449 |  |
| CG8641 | CG8641 |  |
| CG8738 | CG8738 |  |
| CG8778 | CG8778 |  |
| CG8788 | CG8788 |  |
| CG8814 | CG8814 |  |
| CG8910 | CG8910 |  |
| CG8925 | CG8925 |  |
| CG9335 | CG9335 |  |
| CG9486 | CG9486 |  |
| CG9510 | CG9510 |  |
| CG9515 | CG9515 |  |
| CG9527 | CG9527 |  |
| CG9603 | CG9603 |  |
| CG9657 | CG9657 |  |
| CG9662 | CG9662 |  |
| CG9672 | CG9672 |  |
| CG9743 | CG9743 |  |
| CG9796 | CG9796 |  |
| CG9903 | CG9903 |  |
| CG9915 | CG9915 |  |
| CG9917 | CG9917 |  |
| CG14884 | CSN5 | COP9 complex homolog subunit 5 |
| CG42601 | Cad86C | Cad86C |
| CG4236 | Caf1 | Chromatin assembly factor 1 subunit |
| CG1455 | CanA1 | Calcineurin A1 |
| CG7037 | Cbl | Cbl |
| CG4145 | Cg25C | Collagen type IV |
| CG3433 | Coprox | Coproporphyrinogen oxidase |
| CG2555 | Cpr11B | Cuticular protein 11B |
| CG13214 | Cpr47Ef | Cuticular protein 47Ef |
| CG10112 | Cpr51A | Cuticular protein 51A |
| CG13935 | Cpr62Bb | Cuticular protein 62Bb |
| CG15006 | Cpr64Aa | Cuticular protein 64Aa |
| CG1259 | Cpr64Ad | Cuticular protein 64Ad |
| CG2261 | CstF-50 | CstF-50 |
| CG3466 | Cyp4d2 | Cytochrome P450-4d2 |
| CG14686 | Desi | Desiccate |
| CG12370 | Dh44-R2 | Diuretic hormone 44 receptor 2 |
| CG5170 | Dp1 | Dodeca-satellite-binding protein 1 |
| CG7350 | Eig71Ed | Ecdysone-induced gene 71Ed |
| CG10215 | Ercc1 | Ercc1 |
| CG12389 | Fpps | Farnesyl pyrophosphate synthase |
| CG4604 | GLaz | Glial Lazarillo |
| CG9232 | Galt | Galactose-1-phosphate uridylyltransferase |
| CG33206 | Gmap | Golgi microtubule-associated protein |
| CG3495 | Gmer | GDP-4-keto-6-deoxy-D-mannose 3,5-epimerase/4-reductase |
| CG1119 | Gnf1 | Germ line transcription factor 1 |
| CG17527 | GstE5 | Glutathione S transferase E5 |
| CG17530 | GstE6 | Glutathione S transferase E6 |
| CG10658 | Hf | Helical Factor |
| CG3379 | His4r | Histone H4 replacement |
| CG8542 | Hsc70-5 | Heat shock protein cognate 5 |
| CG1059 | Karybeta3 | Karyopherin beta 3 |
| CG5183 | KdelR | KDEL receptor |
| CG10530 | Lcp65Ag1 | Lcp65Ag1 |
| CG10534 | Lcp65Ag2 | Lcp65Ag2 |
| CG18779 | Lcp65Ag3 | Larval cuticle protein |
| CG8523 | Mdr50 | Multi drug resistance 50 |
| CG11301 | Mes4 | Mes4 |
| CG3415 | Mfe2 | peroxisomal Multifunctional enzyme type 2 |
| CG17927 | Mhc | Myosin heavy chain |
| CG4357 | Ncc69 | sodium chloride cotransporter 69 |
| CG9212 | Nipsnap | Nipsnap |
| CG11856 | Nup358 | Nucleoporin 358 |
| CG5427 | Oatp33Ea | Organic anion transporting polypeptide 33Ea |
| CG12905 | Obp46a | Odorant-binding protein 46a |
| CG33478 | Or46a | Odorant receptor 46a |
| CG2890 | PPP4R2r | Protein phosphatase 4 regulatory subunit 2-related protein |
| CG8363 | Papss | PAPS synthetase |
| CG3127 | Pgk | Phosphoglycerate kinase |
| CG4574 | Plc21C | Phospholipase C at 21C |
| CG12110 | Pld | Phospholipase D |
| CG3083 | Prx6005 | Peroxiredoxin 6005 |
| CG3289 | Ptpa | Phosphotyrosyl phosphatase activator |
| CG1447 | Ptx1 | Ptx1 |
| CG12002 | Pxn | Peroxidasin |
| CG7070 | PyK | Pyruvate kinase |
| CG7576 | Rab3 | Rab-protein 3 |
| CG1987 | Rbp1-like | Rbp1-like |
| CG32169 | Rbp6 | RNA-binding protein 6 |
| CG32555 | RhoGAPp190 | RhoGAPp190 |
| CG12775 | RpL21 | Ribosomal protein L21 |
| CG7434 | RpL22 | Ribosomal protein L22 |
| CG6090 | RpL34a | Ribosomal protein L34a |
| CG2998 | RpS28b | Ribosomal protein S28b |
| CG10849 | Sc2 | Sc2 |
| CG6584 | SelR | SelR |
| CG1403 | 1-Sep | Septin-1 |
| CG5094 | Sgt | small glutamine-rich tetratricopeptide containing protein |
| CG11793 | Sod | Superoxide dismutase |
| CG8327 | SpdS | Spermidine Synthase |
| CG7873 | Src42A | Src oncogene at 42A |
| CG8409 | Su(var)205 | Suppressor of variegation 205 |
| CG1467 | Syx16 | Syntaxin 16 |
| CG14503 | Tango8 | Transport and Golgi organization 8 |
| CG4800 | Tctp | Translationally controlled tumor protein |
| CG7654 | Tom20 | Translocase of outer membrane 20 |
| CG9073 | TpnC47D | Troponin C at 47D |
| CG4104 | Tps1 | Trehalose-6-phosphate synthase 1 |
| CG8234 | Tret1-2 | Trehalose transporter 1-2 |
| CG31884 | Trx-2 | thioredoxin-2 |
| CG11326 | Tsp | Thrombospondin |
| CG5471 | TwdlJ | TweedleJ |
| CG5468 | TwdlM | TweedleM |
| CG17932 | Ugt36Bc | Ugt36Bc |
| CG2505 | alpha-Est2 | alpha-Esterase-2 |
| CG9277 | betaTub56D | beta-Tubulin at 56D |
| CG43065 | bru-2 | bruno-2 |
| CG5813 | chif | chiffon |
| CG7554 | comm2 | comm2 |
| CG33516 | dpr3 | dpr3 |
| CG32474 | dys | dysfusion |
| CG12238 | e(y)3 | enhancer of yellow 3 |
| CG31774 | fred | friend of echinoid |
| CG9526 | frj | farjavit |
| CG3218 | fs(1)K10 | female sterile (1) K10 |
| CG6551 | fu | fused |
| CG16785 | fz3 | frizzled 3 |
| CG9623 | if | inflated |
| CG1448 | inx3 | innexin 3 |
| CG5976 | isoQC | iso Glutaminyl cyclase |
| CG3082 | l(2)k09913 | lethal (2) k09913 |
| CG10733 | loj | logjam |
| CG7494 | mRpL1 | mitochondrial ribosomal protein L1 |
| CG11258 | mRpL20 | mitochondrial ribosomal protein L20 |
| CG5818 | mRpL4 | mitochondrial ribosomal protein L4 |
| CG2937 | mRpS2 | mitochondrial ribosomal protein S2 |
| CG5497 | mRpS28 | mitochondrial ribosomal protein S28 |
| CG31217 | modSP | modular serine protease |
| CG18146 | nimC2 | nimrod C2 |
| CG8663 | nrv3 | nervana 3 |
| CG3851 | odd | odd skipped |
| CG7467 | osa | osa |
| CG1212 | p130CAS | p130CAS |
| CG7393 | p38b | p38b |
| CG15812 | pfk | piefke |
| CG5786 | ppan | peter pan |
| CG1837 | prtp | pretaporter |
| CG4444 | px | plexus |
| CG11838 | rempA | reduced mechanoreceptor potential A |
| CG42277 | rn | rotund |
| CG9373 | rump | rumpelstiltskin |
| CG1893 | scramb2 | scramblase 2 |
| CG7113 | scu | scully |
| CG32209 | serp | serpentine |
| CG3135 | shf | shifted |
| CG9339 | sky | skywalker |
| CG4494 | smt3 | smt3 |
| CG10076 | spir | spire |
| CG7855 | timeout | timeout |
| CG34412 | tlk | Tousled-like kinase |
| CG10520 | tub | tube |
| CG11416 | uri | unconventional prefoldin RPB5 interactor |
| CG17914 | yellow-b | yellow-b |
| CG42648 | mRpS34 |  |
| CG42813 | CG42813 |  |
| CG43102 | CG43102 |  |

Heat Shock up only

| Gene > Secondary Identifier | Gene > Symbol | Gene > Name | |
| --- | --- | --- | --- |
| CG17176 | ACXA | ACXA |  |
| CG8626 | Acp53C14a | Acp53C14a | |
| CG15616 | Acp53C14b | Acp53C14b | |
| CG17673 | Acp70A | Accessory gland peptide 70A | |
| CG3801 | Acp76A | Accessory gland-specific peptide 76A | |
| CG7478 | Act79B | Actin 79B |  |
| CG1683 | Ant2 | Adenine nucleotide translocase 2 | |
| CG3675 | Art2 | Arginine methyltransferase 2 | |
| CG5489 | Atg7 | Autophagy-specific gene 7 | |
| CG1567 | C901 | C901 |  |
| CG14375 | CCHa2 | CCHamide-2 | |
| CG17753 | CCS | CCS |  |
| CG10041 | CG10041 |  |  |
| CG10063 | CG10063 |  |  |
| CG10107 | CG10107 |  |  |
| CG10177 | CG10177 |  |  |
| CG10317 | CG10317 |  |  |
| CG10396 | CG10396 |  |  |
| CG10514 | CG10514 |  |  |
| CG10543 | CG10543 |  |  |
| CG10660 | CG10660 |  |  |
| CG10702 | CG10702 |  |  |
| CG10841 | CG10841 |  |  |
| CG10990 | CG10990 |  |  |
| CG11043 | CG11043 |  |  |
| CG11050 | CG11050 |  |  |
| CG11267 | CG11267 |  |  |
| CG11367 | CG11367 |  |  |
| CG11475 | CG11475 |  |  |
| CG11529 | CG11529 |  |  |
| CG11598 | CG11598 |  |  |
| CG11659 | CG11659 |  |  |
| CG11679 | CG11679 |  |  |
| CG11714 | CG11714 |  |  |
| CG11756 | CG11756 |  |  |
| CG11889 | CG11889 |  |  |
| CG11891 | CG11891 |  |  |
| CG11892 | CG11892 |  |  |
| CG11920 | CG11920 |  |  |
| CG12057 | CG12057 |  |  |
| CG12069 | CG12069 |  |  |
| CG12071 | CG12071 |  |  |
| CG12121 | CG12121 |  |  |
| CG12140 | CG12140 |  |  |
| CG12162 | CG12162 |  |  |
| CG12307 | CG12307 |  |  |
| CG12362 | CG12362 |  |  |
| CG12374 | CG12374 |  |  |
| CG12470 | CG12470 |  |  |
| CG12498 | CG12498 |  |  |
| CG12605 | CG12605 |  |  |
| CG12617 | CG12617 |  |  |
| CG12795 | CG12795 |  |  |
| CG12860 | CG12860 |  |  |
| CG12907 | CG12907 |  |  |
| CG12983 | CG12983 |  |  |
| CG12992 | CG12992 |  |  |
| CG13042 | CG13042 |  |  |
| CG13101 | CG13101 |  |  |
| CG13135 | CG13135 |  |  |
| CG1316 | CG1316 |  |  |
| CG13168 | CG13168 |  |  |
| CG13186 | CG13186 |  |  |
| CG13215 | CG13215 |  |  |
| CG13280 | CG13280 |  |  |
| CG13314 | CG13314 |  |  |
| CG13427 | CG13427 |  |  |
| CG13472 | CG13472 |  |  |
| CG13564 | CG13564 |  |  |
| CG13581 | CG13581 |  |  |
| CG13733 | CG13733 |  |  |
| CG13747 | CG13747 |  |  |
| CG13748 | CG13748 |  |  |
| CG13868 | CG13868 |  |  |
| CG13928 | CG13928 |  |  |
| CG13978 | CG13978 |  |  |
| CG13982 | CG13982 |  |  |
| CG14087 | CG14087 |  |  |
| CG14101 | CG14101 |  |  |
| CG14132 | CG14132 |  |  |
| CG1416 | CG1416 |  |  |
| CG14164 | CG14164 |  |  |
| CG14183 | CG14183 |  |  |
| CG14305 | CG14305 |  |  |
| CG14354 | CG14354 |  |  |
| CG14391 | CG14391 |  |  |
| CG14395 | CG14395 |  |  |
| CG14448 | CG14448 |  |  |
| CG14605 | CG14605 |  |  |
| CG14658 | CG14658 |  |  |
| CG14691 | CG14691 |  |  |
| CG14718 | CG14718 |  |  |
| CG14785 | CG14785 |  |  |
| CG14835 | CG14835 |  |  |
| CG14926 | CG14926 |  |  |
| CG14961 | CG14961 |  |  |
| CG14965 | CG14965 |  |  |
| CG15035 | CG15035 |  |  |
| CG15086 | CG15086 |  |  |
| CG15109 | CG15109 |  |  |
| CG15128 | CG15128 |  |  |
| CG15155 | CG15155 |  |  |
| CG15200 | CG15200 |  |  |
| CG15239 | CG15239 |  |  |
| CG15434 | CG15434 |  |  |
| CG15475 | CG15475 |  |  |
| CG15482 | CG15482 |  |  |
| CG15530 | CG15530 |  |  |
| CG15572 | CG15572 |  |  |
| CG15576 | CG15576 |  |  |
| CG15617 | CG15617 |  |  |
| CG15635 | CG15635 |  |  |
| CG15673 | CG15673 |  |  |
| CG15708 | CG15708 |  |  |
| CG15742 | CG15742 |  |  |
| CG16710 | CG16710 |  |  |
| CG16711 | CG16711 |  |  |
| CG16717 | CG16717 |  |  |
| CG16739 | CG16739 |  |  |
| CG16782 | CG16782 |  |  |
| CG16790 | CG16790 |  |  |
| CG17010 | CG17010 |  |  |
| CG17097 | CG17097 |  |  |
| CG17098 | CG17098 |  |  |
| CG17192 | CG17192 |  |  |
| CG17230 | CG17230 |  |  |
| CG17352 | CG17352 |  |  |
| CG17470 | CG17470 |  |  |
| CG17717 | CG17717 |  |  |
| CG17738 | CG17738 |  |  |
| CG17819 | CG17819 |  |  |
| CG1826 | CG1826 |  |  |
| CG18335 | CG18335 |  |  |
| CG18336 | CG18336 |  |  |
| CG18418 | CG18418 |  |  |
| CG18449 | CG18449 |  |  |
| CG18469 | CG18469 |  |  |
| CG18519 | CG18519 |  |  |
| CG18662 | CG18662 |  |  |
| CG1951 | CG1951 |  |  |
| CG1979 | CG1979 |  |  |
| CG2267 | CG2267 |  |  |
| CG2291 | CG2291 |  |  |
| CG3016 | CG3016 |  |  |
| CG30222 | CG30222 |  |  |
| CG30278 | CG30278 |  |  |
| CG30391 | CG30391 |  |  |
| CG3085 | CG3085 |  |  |
| CG3092 | CG3092 |  |  |
| CG31150 | CG31150 |  |  |
| CG31161 | CG31161 |  |  |
| CG31178 | CG31178 |  |  |
| CG31358 | CG31358 |  |  |
| CG31473 | CG31473 |  |  |
| CG31635 | CG31635 |  |  |
| CG31679 | CG31679 |  |  |
| CG31872 | CG31872 |  |  |
| CG31924 | CG31924 |  |  |
| CG32081 | CG32081 |  |  |
| CG32138 | CG32138 |  |  |
| CG3222 | CG3222 |  |  |
| CG32236 | CG32236 |  |  |
| CG32238 | CG32238 |  |  |
| CG3226 | CG3226 |  |  |
| CG32369 | CG32369 |  |  |
| CG32396 | CG32396 |  |  |
| CG32436 | CG32436 |  |  |
| CG32459 | CG32459 |  |  |
| CG32462 | CG32462 |  |  |
| CG32628 | CG32628 |  |  |
| CG32650 | CG32650 |  |  |
| CG33271 | CG33271 |  |  |
| CG33340 | CG33340 |  |  |
| CG3345 | CG3345 |  |  |
| CG34104 | CG34104 |  |  |
| CG34354 | CG34354 |  |  |
| CG34375 | CG34375 |  |  |
| CG34394 | CG34394 |  |  |
| CG3492 | CG3492 |  |  |
| CG3513 | CG3513 |  |  |
| CG3517 | CG3517 |  |  |
| CG3638 | CG3638 |  |  |
| CG3790 | CG3790 |  |  |
| CG3884 | CG3884 |  |  |
| CG3927 | CG3927 |  |  |
| CG3982 | CG3982 |  |  |
| CG4068 | CG4068 |  |  |
| CG4161 | CG4161 |  |  |
| CG4480 | CG4480 |  |  |
| CG4669 | CG4669 |  |  |
| CG4691 | CG4691 |  |  |
| CG4712 | CG4712 |  |  |
| CG4836 | CG4836 |  |  |
| CG4907 | CG4907 |  |  |
| CG5056 | CG5056 |  |  |
| CG5103 | CG5103 |  |  |
| CG5177 | CG5177 |  |  |
| CG5180 | CG5180 |  |  |
| CG5217 | CG5217 |  |  |
| CG5343 | CG5343 |  |  |
| CG5521 | CG5521 |  |  |
| CG5525 | CG5525 |  |  |
| CG5556 | CG5556 |  |  |
| CG5565 | CG5565 |  |  |
| CG5790 | CG5790 |  |  |
| CG5805 | CG5805 |  |  |
| CG5987 | CG5987 |  |  |
| CG6012 | CG6012 |  |  |
| CG6091 | CG6091 |  |  |
| CG6129 | CG6129 |  |  |
| CG6145 | CG6145 |  |  |
| CG6262 | CG6262 |  |  |
| CG6279 | CG6279 |  |  |
| CG6300 | CG6300 |  |  |
| CG6418 | CG6418 |  |  |
| CG6424 | CG6424 |  |  |
| CG6441 | CG6441 |  |  |
| CG6497 | CG6497 |  |  |
| CG6511 | CG6511 |  |  |
| CG6527 | CG6527 |  |  |
| CG6628 | CG6628 |  |  |
| CG6661 | CG6661 |  |  |
| CG6709 | CG6709 |  |  |
| CG6776 | CG6776 |  |  |
| CG6785 | CG6785 |  |  |
| CG6792 | CG6792 |  |  |
| CG6928 | CG6928 |  |  |
| CG6966 | CG6966 |  |  |
| CG7024 | CG7024 |  |  |
| CG7099 | CG7099 |  |  |
| CG7131 | CG7131 |  |  |
| CG7140 | CG7140 |  |  |
| CG7154 | CG7154 |  |  |
| CG7202 | CG7202 |  |  |
| CG7251 | CG7251 |  |  |
| CG7309 | CG7309 |  |  |
| CG7381 | CG7381 |  |  |
| CG7387 | CG7387 |  |  |
| CG7420 | CG7420 |  |  |
| CG7441 | CG7441 |  |  |
| CG7510 | CG7510 |  |  |
| CG7567 | CG7567 |  |  |
| CG7634 | CG7634 |  |  |
| CG7707 | CG7707 |  |  |
| CG7720 | CG7720 |  |  |
| CG7742 | CG7742 |  |  |
| CG7755 | CG7755 |  |  |
| CG7804 | CG7804 |  |  |
| CG7879 | CG7879 |  |  |
| CG7945 | CG7945 |  |  |
| CG7946 | CG7946 |  |  |
| CG8043 | CG8043 |  |  |
| CG8086 | CG8086 |  |  |
| CG8129 | CG8129 |  |  |
| CG8297 | CG8297 |  |  |
| CG8389 | CG8389 |  |  |
| CG8517 | CG8517 |  |  |
| CG8564 | CG8564 |  |  |
| CG8813 | CG8813 |  |  |
| CG8838 | CG8838 |  |  |
| CG8839 | CG8839 |  |  |
| CG8907 | CG8907 |  |  |
| CG8908 | CG8908 |  |  |
| CG8918 | CG8918 |  |  |
| CG9090 | CG9090 |  |  |
| CG9129 | CG9129 |  |  |
| CG9153 | CG9153 |  |  |
| CG9173 | CG9173 |  |  |
| CG9222 | CG9222 |  |  |
| CG9254 | CG9254 |  |  |
| CG9293 | CG9293 |  |  |
| CG9297 | CG9297 |  |  |
| CG9314 | CG9314 |  |  |
| CG9389 | CG9389 |  |  |
| CG9392 | CG9392 |  |  |
| CG9570 | CG9570 |  |  |
| CG9589 | CG9589 |  |  |
| CG9624 | CG9624 |  |  |
| CG9650 | CG9650 |  |  |
| CG9776 | CG9776 |  |  |
| CG9996 | CG9996 |  |  |
| CG6198 | CHORD | CHORD |  |
| CG11778 | CNT1 | Concentrative Nucleoside Transporter 1 | |
| CR30029 | CR30029 |  |  |
| CR31781 | CR31781 |  |  |
| CR33317 | CR33317 |  |  |
| CG3725 | Ca-P60A | Calcium ATPase at 60A | |
| CG16708 | Cerk | Ceramide kinase | |
| CG8914 | CkIIbeta2 | Casein kinase II beta2 subunit | |
| CG7391 | Clk | Clock |  |
| CG3433 | Coprox | Coproporphyrinogen oxidase | |
| CG6517 | Cp18 | Chorion protein 18 | |
| CG10243 | Cyp6a19 | Cyp6a19 |  |
| CG10245 | Cyp6a20 | Cyp6a20 |  |
| CG11347 | DOR |  |  |
| CG6169 | Dcp2 | Decapping protein 2 | |
| CG6493 | Dcr-2 | Dicer-2 |  |
| CG8348 | Dh44 | Diuretic hormone 44 | |
| CG4323 | Dic2 | Dicarboxylate carrier 2 | |
| CG9828 | DnaJ-H | DnaJ homolog | |
| CG11591 | Dpy-30L2 | Dpy-30-like 2 | |
| CG8863 | Droj2 | DnaJ-like-2 | |
| CG12756 | Eaf6 | Eaf6 |  |
| CG5345 | Eip55E | Eip55E |  |
| CG4535 | FKBP59 | FK506-binding protein FKBP59 | |
| CG3763 | Fbp2 | Fat body protein 2 | |
| CG6128 | Fuca | alpha-L-fucosidase | |
| CG17257 | GABPI | beta4GalNAcTB pilot | |
| CG1152 | Gld | Glucose dehydrogenase | |
| CG14979 | Gr63a | Gustatory receptor 63a | |
| CG7189 | Gr66a | Gustatory receptor 66a | |
| CG14901 | Gr89a | Gustatory receptor 89a | |
| CG7975 | Grx-1 | Grx-1 |  |
| CG18548 | GstD10 | Glutathione S transferase D10 | |
| CG4423 | GstD6 | Glutathione S transferase D6 | |
| CG1470 | Gycbeta100B | Guanylyl cyclase beta-subunit at 100B | |
| CG2720 | Hop | Hsp70/Hsp90 organizing protein homolog | |
| CG7756 | Hsc70-2 | Heat shock protein cognate 2 | |
| CG4147 | Hsc70-3 | Heat shock protein cognate 3 | |
| CG6603 | Hsc70Cb | Hsc70Cb |  |
| CG5748 | Hsf | Heat shock factor | |
| CG12101 | Hsp60 | Heat shock protein 60 | |
| CG7235 | Hsp60C | Hsp60C |  |
| CG16954 | Hsp60D | Hsp60D |  |
| CG4167 | Hsp67Ba | Heat shock gene 67Ba | |
| CG31795 | IA-2 | IA-2 ortholog | |
| CG2723 | ImpE3 | Ecdysone-inducible gene E3 | |
| CG1120 | IntS10 | Integrator 10 | |
| CG2961 | Ipod | Interaction partner of Dnmt2 | |
| CG8867 | Jon25Bi | Jonah 25Bi | |
| CG18030 | Jon99Fi | Jonah 99Fi |  |
| CG3962 | Keap1 | Keap1 |  |
| CG3219 | Klp59C | Klp59C |  |
| CG10119 | LamC | Lamin C |  |
| CG3849 | Lasp | Lasp |  |
| CG10530 | Lcp65Ag1 | Lcp65Ag1 |  |
| CG10534 | Lcp65Ag2 | Lcp65Ag2 |  |
| CG18779 | Lcp65Ag3 | Larval cuticle protein | |
| CG9057 | Lsd-2 | Lipid storage droplet-2 | |
| CG4178 | Lsp1beta | Larval serum protein 1 beta | |
| CG6821 | Lsp1gamma | Larval serum protein 1 gamma | |
| CG42572 | MCPH1 | Microcephalin | |
| CG30388 | Magi | Magi |  |
| CG14934 | Mal-B1 | Maltase B1 | |
| CG1775 | Med | Medea |  |
| CG7964 | Menl-1 | Malic enzyme like-1 | |
| CG7969 | Menl-2 | Malic enzyme like-2 | |
| CG6803 | Mf | Myofilin |  |
| CG8295 | Mlf | Myelodysplasia/myeloid leukemia factor | |
| CG33048 | Mocs1 | Molybdenum cofactor synthesis 1 ortholog | |
| CG9074 | Mst57Da | Male-specific RNA 57Da | |
| CG5016 | Mst57Db | Male-specific RNA 57Db | |
| CG4986 | Mst57Dc | Male-specific RNA 57Dc | |
| CG11993 | Mst85C | Mst85C |  |
| CG32602 | Muc12Ea | Mucin 12Ea | |
| CG18331 | Muc68Ca | Mucin 68Ca | |
| CG32721 | NELF-B | NELF-B |  |
| CG32190 | NUCB1 | NUCB1 |  |
| CG1553 | Nop17l | Nop17 like | |
| CG3811 | Oatp30B | Organic anion transporting polypeptide 30B | |
| CG17871 | Or71a | Odorant receptor 71a | |
| CG2087 | PEK | pancreatic eIF-2alpha kinase | |
| CG31794 | Pax | Paxillin |  |
| CG17645 | Pglym87 | Pglym87 |  |
| CG42349 | Pkcdelta | Protein kinase C delta | |
| CG17137 | Porin2 | Porin2 |  |
| CG31025 | Ppi1 | Protein phosphatase 1c interacting protein 1 | |
| CG5648 | Prosalpha6T | Proteasome alpha6T subunit | |
| CG9100 | Rab30 | Rab30 |  |
| CG42378 | RhoGEF3 |  |  |
| CG7014 | RpS5b | Ribosomal protein S5b | |
| CG10149 | Rpn6 | Proteasome p44.5 subunit | |
| CG10844 | Rya-r44F | Ryanodine receptor 44F | |
| CG4385 | S | Star |  |
| CG32064 | S-Lap4 | Sperm-Leucylaminopeptidase 4 | |
| CG13340 | S-Lap7 | Sperm-Leucylaminopeptidase 7 | |
| CG4439 | S-Lap8 | Sperm-Leucylaminopeptidase 8 | |
| CG13164 | SIP2 | Syntaxin Interacting Protein 2 | |
| CG11006 | Sap130 | Sin3A-associated protein 130 | |
| CG9617 | Sgt1 |  |  |
| CG5216 | Sir2 | Sir2 |  |
| CG11305 | Sirt7 | Sirt7 |  |
| CG8137 | Spn2 | Serine protease inhibitor 2 | |
| CG9334 | Spn3 | Serine protease inhibitor 3 | |
| CG6289 | Spn77Bc | Serpin 77Bc | |
| CG18255 | Strn-Mlck | Stretchin-Mlck | |
| CG7452 | Syx17 | Syntaxin 17 | |
| CG11201 | TTLL3B | Tubulin tyrosine ligase-like 3B | |
| CG2670 | Taf7 | TBP-associated factor 7 | |
| CG7128 | Taf8 | TBP-associated factor 8 | |
| CG4821 | Tequila | Tequila |  |
| CG10415 | TfIIEalpha | Transcription factor IIEalpha | |
| CG4843 | Tm2 | Tropomyosin 2 | |
| CG15104 | Topors | Topoisomerase I-interacting protein | |
| CG2857 | Tpc2 | Thiamine pyrophosphate carrier protein 2 | |
| CG2848 | Trn-SR | Transportin-Serine/Arginine rich | |
| CG3315 | TrxT | Thioredoxin T | |
| CG12841 | Tsp42Ek | Tetraspanin 42Ek | |
| CG16991 | Tsp66A | Tetraspanin 66A | |
| CG12799 | Ubc84D | Ubiquitin conjugating enzyme 84D | |
| CG14224 | Ubqn | Ubiquilin |  |
| CG6644 | Ugt35a | UDP-glycosyltransferase 35a | |
| CG33519 | Unc-89 | Unc-89 |  |
| CG5075 | Vha68-3 | Vacuolar H[+] ATPase subunit 68-3 | |
| CG1121 | alpha-Est8 | alpha-Esterase-8 | |
| CG4241 | att-ORFA | alternative testis transcripts open reading frame A | |
| CG33488 | att-ORFB | alternative testis transcripts open reading frame B | |
| CG3068 | aur | aurora |  |
| CG42230 | bbg | big bang |  |
| CG10630 | blanks | blanks |  |
| CG42281 | bun | bunched |  |
| CG12135 | c12.1 | c12.1 |  |
| CG7134 | cdc14 | cdc14 |  |
| CG5813 | chif | chiffon |  |
| CG7595 | ck | crinkled |  |
| CG4832 | cnn | centrosomin | |
| CG7554 | comm2 | comm2 |  |
| CG12489 | dnr1 | defense repressor 1 | |
| CG9696 | dom | domino |  |
| CG31361 | dpr17 | dpr17 |  |
| CG8277 | eIF4E-5 | eIF4E-5 |  |
| CG7919 | fan | farinelli |  |
| CG6817 | foi | fear-of-intimacy | |
| CG9739 | fz2 | frizzled 2 |  |
| CG16783 | fzr2 | fizzy-related 2 | |
| CG3183 | geminin | geminin |  |
| CG4353 | hep | hemipterous | |
| CG31000 | heph | hephaestus | |
| CG9484 | hyd | hyperplastic discs | |
| CG8873 | jet | jetlag |  |
| CG11546 | kermit | kermit |  |
| CG32742 | l(1)G0148 | lethal (1) G0148 | |
| CG4943 | lack | lethal with a checkpoint kinase | |
| CG17334 | lin-28 | lin-28 |  |
| CG4677 | lmd | lame duck |  |
| CG11896 | m-cup | mann-cup |  |
| CG8342 | m1 | E(spl) region transcript m1 | |
| CG11258 | mRpL20 | mitochondrial ribosomal protein L20 | |
| CG13852 | mats | mob as tumor suppressor | |
| CG31991 | mdy | midway |  |
| CG14981 | mge | maggie |  |
| CG17492 | mib2 | mind bomb 2 | |
| CG14560 | msopa | male-specific opa containing gene | |
| CG17248 | n-syb | n-synaptobrevin | |
| CG3620 | norpA | no receptor potential A | |
| CG10855 | ntc | nutcracker | |
| CG9710 | nudC | nudC |  |
| CG7929 | ocn | ocnus |  |
| CG11430 | olf186-F | olf186-F |  |
| CG14489 | olf186-M | olf186-M |  |
| CG3428 | pall | pallbearer |  |
| CG7081 | pex2 | peroxin 2 |  |
| CG42670 | ps | pasilla |  |
| CG10998 | r-cup | ryder cup |  |
| CG7061 | rab3-GAP | rab3-GAP |  |
| CG9528 | retm | real-time |  |
| CG32096 | rols | rolling pebbles | |
| CG31152 | rumi | rumi |  |
| CG33193 | sav | salvador |  |
| CG17736 | schuy | schumacher-levy | |
| CG1472 | sec24 | sec24 |  |
| CG18102 | shi | shibire |  |
| CG13030 | sinah | sina homologue | |
| CG32434 | siz | schizo |  |
| CG9339 | sky | skywalker |  |
| CG1417 | slgA | sluggish A |  |
| CG9131 | slmo | slowmo |  |
| CG7390 | smp-30 | Senescence marker protein-30 | |
| CG4494 | smt3 | smt3 |  |
| CG30365 | spaw | spacewatch | |
| CG14735 | ssp5 | short spindle 5 | |
| CG15179 | sunz | sungrazer |  |
| CG18000 | sw | short wing | |
| CG12120 | t | tan |  |
| CG4719 | tankyrase | tankyrase |  |
| CG6868 | tld | tolloid |  |
| CG9660 | toc | toucan |  |
| CG14690 | tomboy20 | tomboy20 |  |
| CG8330 | tomboy40 | tomboy40 |  |
| CG16724 | tra | transformer | |
| CG18345 | trpl | trp-like |  |
| CG12313 | ttm2 | tiny tim 2 |  |
| CG31137 | twin | twin |  |
| CG7107 | up | upheld |  |
| CG13176 | wash | washout |  |
| CG12250 | ymp | yellow-emperor | |
| CG43343 | CG43343 |  |  |
| CG42288 | CG42288 |  | Also found in age down |
| CG34107 | CG34107 |  | Also found in age down |
| CG42863 | CG42863 |  |  |

Heat Shock down only

| Gene > Secondary Identifier | Gene > Symbol | Gene > Name |
| --- | --- | --- |
| CG1168 | 7B2 | 7B2 |
| CG11471 | Aats-ile | Isoleucyl-tRNA synthetase |
| CG9390 | AcCoAS | Acetyl Coenzyme A synthase |
| CG9621 | Adgf-D | Adenosine deaminase-related growth factor D |
| CG9538 | Ag5r | Antigen 5-related |
| CG6554 | Art1 | Arginine methyltransferase 1 |
| CG32446 | Atox1 |  |
| CG1471 | CDase | Ceramidase |
| CG10131 | CG10131 |  |
| CG10157 | CG10157 |  |
| CG10175 | CG10175 |  |
| CG10433 | CG10433 |  |
| CG10444 | CG10444 |  |
| CG10688 | CG10688 |  |
| CG1074 | CG1074 |  |
| CG10911 | CG10911 |  |
| CG10912 | CG10912 |  |
| CG10992 | CG10992 |  |
| CG11123 | CG11123 |  |
| CG1143 | CG1143 |  |
| CG11436 | CG11436 |  |
| CG11842 | CG11842 |  |
| CG11852 | CG11852 |  |
| CG11854 | CG11854 |  |
| CG11897 | CG11897 |  |
| CG11899 | CG11899 |  |
| CG11926 | CG11926 |  |
| CG11985 | CG11985 |  |
| CG12016 | CG12016 |  |
| CG1208 | CG1208 |  |
| CG12253 | CG12253 |  |
| CG12272 | CG12272 |  |
| CG1236 | CG1236 |  |
| CG12384 | CG12384 |  |
| CG12424 | CG12424 |  |
| CG1275 | CG1275 |  |
| CG12780 | CG12780 |  |
| CG12926 | CG12926 |  |
| CG13117 | CG13117 |  |
| CG13315 | CG13315 |  |
| CG13323 | CG13323 |  |
| CG13324 | CG13324 |  |
| CG13704 | CG13704 |  |
| CG13794 | CG13794 |  |
| CG13810 | CG13810 |  |
| CG13933 | CG13933 |  |
| CG14105 | CG14105 |  |
| CG14125 | CG14125 |  |
| CG14273 | CG14273 |  |
| CG14523 | CG14523 |  |
| CG1461 | CG1461 |  |
| CG14630 | CG14630 |  |
| CG14645 | CG14645 |  |
| CG14782 | CG14782 |  |
| CG14933 | CG14933 |  |
| CG14949 | CG14949 |  |
| CG14977 | CG14977 |  |
| CG15019 | CG15019 |  |
| CG15065 | CG15065 |  |
| CG15067 | CG15067 |  |
| CG15083 | CG15083 |  |
| CG15098 | CG15098 |  |
| CG15152 | CG15152 |  |
| CG15210 | CG15210 |  |
| CG15394 | CG15394 |  |
| CG15422 | CG15422 |  |
| CG1561 | CG1561 |  |
| CG1572 | CG1572 |  |
| CG15771 | CG15771 |  |
| CG1578 | CG1578 |  |
| CG15818 | CG15818 |  |
| CG1607 | CG1607 |  |
| CG16743 | CG16743 |  |
| CG16965 | CG16965 |  |
| CG1707 | CG1707 |  |
| CG17278 | CG17278 |  |
| CG17737 | CG17737 |  |
| CG17904 | CG17904 |  |
| CG18048 | CG18048 |  |
| CG18067 | CG18067 |  |
| CG18095 | CG18095 |  |
| CG18292 | CG18292 |  |
| CG18522 | CG18522 |  |
| CG18537 | CG18537 |  |
| CG18539 | CG18539 |  |
| CG18540 | CG18540 |  |
| CG18619 | CG18619 |  |
| CG18636 | CG18636 |  |
| CG18661 | CG18661 |  |
| CG1889 | CG1889 |  |
| CG1907 | CG1907 |  |
| CG2004 | CG2004 |  |
| CG2698 | CG2698 |  |
| CG2811 | CG2811 |  |
| CG2846 | CG2846 |  |
| CG2926 | CG2926 |  |
| CG31030 | CG31030 |  |
| CG31098 | CG31098 |  |
| CG31199 | CG31199 |  |
| CG31248 | CG31248 |  |
| CG31324 | CG31324 |  |
| CG31380 | CG31380 |  |
| CG31743 | CG31743 |  |
| CG31823 | CG31823 |  |
| CG32054 | CG32054 |  |
| CG32428 | CG32428 |  |
| CG3257 | CG3257 |  |
| CG32649 | CG32649 |  |
| CG32810 | CG32810 |  |
| CG33082 | CG33082 |  |
| CG33123 | CG33123 |  |
| CG33307 | CG33307 |  |
| CG3505 | CG3505 |  |
| CG3590 | CG3590 |  |
| CG3604 | CG3604 |  |
| CG3662 | CG3662 |  |
| CG3759 | CG3759 |  |
| CG3781 | CG3781 |  |
| CG3800 | CG3800 |  |
| CG3835 | CG3835 |  |
| CG3860 | CG3860 |  |
| CG42335 | CG42335 |  |
| CG4250 | CG4250 |  |
| CG42542 | CG42542 |  |
| CG4259 | CG4259 |  |
| CG42668 | CG42668 |  |
| CG4341 | CG4341 |  |
| CG43427 | CG43427 |  |
| CG4716 | CG4716 |  |
| CG4741 | CG4741 |  |
| CG4781 | CG4781 |  |
| CG4783 | CG4783 |  |
| CG4858 | CG4858 |  |
| CG4942 | CG4942 |  |
| CG5126 | CG5126 |  |
| CG5189 | CG5189 |  |
| CG5399 | CG5399 |  |
| CG5431 | CG5431 |  |
| CG5508 | CG5508 |  |
| CG5541 | CG5541 |  |
| CG5793 | CG5793 |  |
| CG5819 | CG5819 |  |
| CG5910 | CG5910 |  |
| CG6115 | CG6115 |  |
| CG6188 | CG6188 |  |
| CG6283 | CG6283 |  |
| CG6337 | CG6337 |  |
| CG6357 | CG6357 |  |
| CG6385 | CG6385 |  |
| CG6409 | CG6409 |  |
| CG6429 | CG6429 |  |
| CG6453 | CG6453 |  |
| CG6475 | CG6475 |  |
| CG6553 | CG6553 |  |
| CG6673 | CG6673 |  |
| CG6690 | CG6690 |  |
| CG6769 | CG6769 |  |
| CG6900 | CG6900 |  |
| CG6910 | CG6910 |  |
| CG6981 | CG6981 |  |
| CG7016 | CG7016 |  |
| CG7054 | CG7054 |  |
| CG7324 | CG7324 |  |
| CG7338 | CG7338 |  |
| CG7470 | CG7470 |  |
| CG7488 | CG7488 |  |
| CG7911 | CG7911 |  |
| CG7979 | CG7979 |  |
| CG8046 | CG8046 |  |
| CG8066 | CG8066 |  |
| CG8112 | CG8112 |  |
| CG8132 | CG8132 |  |
| CG8230 | CG8230 |  |
| CG8249 | CG8249 |  |
| CG8303 | CG8303 |  |
| CG8317 | CG8317 |  |
| CG8358 | CG8358 |  |
| CG8468 | CG8468 |  |
| CG8860 | CG8860 |  |
| CG8924 | CG8924 |  |
| CG8939 | CG8939 |  |
| CG9009 | CG9009 |  |
| CG9270 | CG9270 |  |
| CG9286 | CG9286 |  |
| CG9312 | CG9312 |  |
| CG9319 | CG9319 |  |
| CG9336 | CG9336 |  |
| CG9363 | CG9363 |  |
| CG9400 | CG9400 |  |
| CG9628 | CG9628 |  |
| CG9629 | CG9629 |  |
| CG9630 | CG9630 |  |
| CG9631 | CG9631 |  |
| CG9649 | CG9649 |  |
| CG9780 | CG9780 |  |
| CG9886 | CG9886 |  |
| CG9997 | CG9997 |  |
| CG5203 | CHIP | CHIP |
| CG1411 | CRMP | Collapsin Response Mediator Protein |
| CG6871 | Cat | Catalase |
| CG1962 | Cen | Centrocortin |
| CG3637 | Cortactin | Cortactin |
| CG6395 | Csp | Cysteine string protein |
| CG6042 | Cyp12a4 | Cyp12a4 |
| CG1488 | Cyp311a1 | Cyp311a1 |
| CG2060 | Cyp4e2 | Cytochrome P450-4e2 |
| CG10842 | Cyp4p1 | Cytochrome P450-4p1 |
| CG2397 | Cyp6a13 | Cyp6a13 |
| CG10241 | Cyp6a17 | Cyp6a17 |
| CG17577 | Cyp9h1 | Cyp9h1 |
| CG42234 | Dbx | Dbx |
| CG4665 | Dhpr | Dihydropteridine reductase |
| CG6413 | Dis3 | Dis3 |
| CG18741 | DopR2 | Dopamine receptor 2 |
| CG12223 | Dsp1 | Dorsal switch protein 1 |
| CG8280 | Ef1alpha48D | Elongation factor 1alpha48D |
| CG9291 | Elongin-C | Elongin C |
| CG1474 | Es2 | Es2 |
| CG4144 | GNBP2 | Gram-negative bacteria binding protein 2 |
| CG12030 | Gale | UDP-galactose 4'-epimerase |
| CG5288 | Galk | Galactokinase |
| CG8430 | Got1 | Glutamate oxaloacetate transaminase 1 |
| CG5820 | Gp150 | Gp150 |
| CG13417 | Gr93a | Gustatory receptor 93a |
| CG17525 | GstE4 | Glutathione S transferase E4 |
| CG12855 | HPS1 | Hermansky-Pudlak Syndrome 1 ortholog |
| CG17950 | HmgD | High mobility group protein D |
| CG7399 | Hn | Henna |
| CG18108 | IM1 | Immune induced molecule 1 |
| CG18106 | IM2 | Immune induced molecule 2 |
| CG15066 | IM23 | Immune induced molecule 23 |
| CG16844 | IM3 | Immune induced molecule 3 |
| CG15231 | IM4 | Immune induced molecule 4 |
| CG4559 | Idgf3 | Imaginal disc growth factor 3 |
| CG5154 | Idgf5 | Imaginal disc growth factor 5 |
| CG7176 | Idh | Isocitrate dehydrogenase |
| CG30295 | Ipk1 | Ipk1 |
| CG8913 | Irc | Immune-regulated catalase |
| CG9423 | Kap-alpha3 | karyopherin alpha3 |
| CR33790 | Kaz1-ORFA | Kaz1-ORFA |
| CG1220 | Kaz1-ORFB | Kaz1-ORFB |
| CG7144 | LKR | lysine ketoglutarate reductase |
| CG12369 | Lac | Lachesin |
| CG8823 | Lip3 | Lip3 |
| CG1179 | LysB | Lysozyme B |
| CG9111 | LysC | Lysozyme C |
| CG9118 | LysD | Lysozyme D |
| CG1180 | LysE | Lysozyme E |
| CG3034 | MED22 | Mediator complex subunit 22 |
| CG15669 | MESK2 | Misexpression suppressor of KSR 2 |
| CG15162 | MESR3 | Misexpression suppressor of ras 3 |
| CG1102 | MP1 | Melanization Protease 1 |
| CG3879 | Mdr49 | Multi drug resistance 49 |
| CG10701 | Moe | Moesin |
| CG9470 | MtnA | Metallothionein A |
| CG32656 | Muc11A | Mucin 11A |
| CG5258 | NHP2 | NHP2 |
| CG5330 | Nap1 | Nucleosome assembly protein 1 |
| CG7067 | NitFhit | Nitrilase and fragile histidine triad fusion protein |
| CG13849 | Nop56 | Nop56 |
| CG7421 | Nopp140 | Nopp140 |
| CG6417 | Oatp33Eb | Organic anion transporting polypeptide 33Eb |
| CG15883 | Obp18a | Odorant-binding protein 18a |
| CG11748 | Obp19a | Odorant-binding protein 19a |
| CG11422 | Os-E | Olfactory-specific E |
| CG15188 | Osi20 | Osiris 20 |
| CG7496 | PGRP-SD | PGRP-SD |
| CG10436 | Pbprp1 | Pheromone-binding protein-related protein 1 |
| CG11421 | Pbprp3 | Pheromone-binding protein-related protein 3 |
| CG5547 | Pect | Phosphoethanolamine cytidylyltransferase |
| CG3400 | Pfrx | 6-phosphofructo-2-kinase |
| CG5373 | Pi3K59F | Phosphotidylinositol 3 kinase 59F |
| CG5629 | Ppcs | Phosphopantothenoylcysteine synthetase |
| CG8877 | Prp8 | pre-mRNA processing factor 8 |
| CG3129 | Rab-RP4 | Rab-related protein 4 |
| CG17060 | Rab10 | Rab-protein 10 |
| CG5915 | Rab7 | Rab-protein 7 |
| CG2248 | Rac1 | Rac1 |
| CG10354 | Rat1 |  |
| CG3200 | Reg-2 | Rhythmically expressed gene 2 |
| CG8416 | Rho1 | Rho1 |
| CG1554 | RpII215 | RNA polymerase II 215kD subunit |
| CG16916 | Rpt3 | Rpt3 |
| CG3051 | SNF1A | SNF1A/AMP-activated protein kinase |
| CG2674 | Sam-S | S-adenosylmethionine Synthetase |
| CG2471 | Sclp | Sclp |
| CG4649 | Sodh-2 | Sorbitol dehydrogenase-2 |
| CG3066 | Sp7 | Serine protease 7 |
| CG11331 | Spn27A | Serpin 27A |
| CG6680 | Spn77Ba | Serpin 77Ba |
| CG9126 | Stim | Stromal interaction molecule |
| CG5723 | Ten-m | Tenascin major |
| CG5163 | TfIIA-S | Transcription-factor-IIA-S |
| CG3024 | Torsin | Torsin |
| CG4394 | Traf-like | TNF-receptor-associated factor-like |
| CG33261 | Trl | Trithorax-like |
| CG3666 | Tsf3 | Transferrin 3 |
| CG11415 | Tsp2A | Tetraspanin 2A |
| CG10106 | Tsp42Ee | Tetraspanin 42Ee |
| CG12142 | Tsp42Eg | Tetraspanin 42Eg |
| CG2257 | Ubc-E2H | Ubc-E2H |
| CG7171 | Uro | Urate oxidase |
| CG3161 | Vha16-1 | Vacuolar H[+] ATPase subunit 16-1 |
| CG2934 | VhaAC39-1 | Vacuolar H[+] ATPase subunit AC39-1 |
| CG7625 | VhaM9.7-b | Vacuolar H[+] ATPase subunit M9.7-b |
| CG11589 | VhaM9.7-c | Vacuolar H[+] ATPase subunit M9.7-c |
| CG7007 | VhaPPA1-1 | Vacuolar H[+] ATPase subunit PPA1-1 |
| CG14804 | Vps26 | Vacuolar protein sorting 26 |
| CG1089 | alpha-Est5 | alpha-Esterase-5 |
| CG42275 | alpha-Man-I | alpha Mannosidase I |
| CG6438 | amon | amontillado |
| CG11579 | arm | armadillo |
| CG4276 | aru | arouser |
| CG1107 | aux | auxillin |
| CG3717 | bcn92 | bcn92 |
| CG3644 | bic | bicaudal |
| CG3350 | bigmax | bigmax |
| CG5680 | bsk | basket |
| CG16987 | daw | dawdle |
| CG7098 | dik | diskette |
| CG10161 | eIF-3p66 | Eukaryotic initiation factor 3 p66 subunit |
| CG3525 | eas | easily shocked |
| CG12386 | etaTry | etaTrypsin |
| CG7127 | exo70 | exo70 |
| CG7773 | fidipidine | fidipidine |
| CG15825 | fon | fondue |
| CG3874 | frc | fringe connection |
| CG2944 | gus | gustavus |
| CG1623 | hebe | hebe |
| CG4779 | hgo | homogentisate 1,2-dioxygenase |
| CG9078 | ifc | infertile crescent |
| CG18285 | igl | igloo |
| CG17383 | jigr1 | jing interacting gene regulatory 1 |
| CG6302 | l(3)01239 | lethal (3) 01239 |
| CG32210 | l(3)76BDr |  |
| CG4162 | lace | lace |
| CG2958 | lectin-24Db | lectin-24Db |
| CG11488 | mRpL10 | mitochondrial ribosomal protein L10 |
| CG9353 | mRpL54 | mitochondrial ribosomal protein L54 |
| CG1221 | miple | miple |
| CG2050 | mod | modulo |
| CG1857 | nec | necrotic |
| CG9655 | nes | nessy |
| CG31839 | nimB2 | nimrod B2 |
| CG8942 | nimC1 | nimrod C1 |
| CG6501 | ns2 | nucleostemin 2 |
| CG43479 | nwk | nervous wreck |
| CG14779 | pck | pickel |
| CG8241 | pea | peanuts |
| CG8705 | pnut | peanut |
| CG8588 | pst | pastrel |
| CG4067 | pug | pugilist |
| CG31240 | repo | reversed polarity |
| CG7642 | ry | rosy |
| CG11124 | sPLA2 | secretory Phospholipase A2 |
| CG12789 | santa-maria | scavenger receptor acting in neural tissue and majority of rhodopsin is absent |
| CG6159 | sec10 | sec10 |
| CG8055 | shrb | shrub |
| CG3992 | srp | serpent |
| CG6863 | tok | tolkin |
| CG4254 | tsr | twinstar |
| CG16707 | vsg | visgun |
| CG18550 | yellow-f | yellow-f |
| CG42825 | CG42825 |  |

Irradiation up only

| Gene > Secondary Identifier | Gene > Symbol | Gene > Name |
| --- | --- | --- |
| CG7439 | AGO2 | Argonaute 2 |
| CG7828 | APP-BP1 | beta-Amyloid precursor protein binding protein 1 |
| CG13391 | Aats-ala | Alanyl-tRNA synthetase |
| CG12141 | Aats-lys | Lysyl-tRNA synthetase |
| CG9638 | Ada2b | Ada2b |
| CG17146 | Adk1 | Adenylate kinase-1 |
| CG12534 | Alr | Augmenter of liver regeneration |
| CG9579 | AnnX | Annexin X |
| CG12235 | Arp11 | Arp11 |
| CG8421 | Asph | Aspartyl beta-hydroxylase |
| CG9204 | Ate1 | Ate1 |
| CG5429 | Atg6 | Autophagy-specific gene 6 |
| CG3615 | Atg9 | Autophagy-specific gene 9 |
| CG4272 | Axud1 |  |
| CG18408 | CAP | CAP |
| CG14358 | CCHa1 | CCHamide-1 |
| CG10251 | CG10251 |  |
| CG10277 | CG10277 |  |
| CG10306 | CG10306 |  |
| CG10341 | CG10341 |  |
| CG10732 | CG10732 |  |
| CG11106 | CG11106 |  |
| CG11414 | CG11414 |  |
| CG11695 | CG11695 |  |
| CG11710 | CG11710 |  |
| CG11982 | CG11982 |  |
| CG12065 | CG12065 |  |
| CG12203 | CG12203 |  |
| CG1233 | CG1233 |  |
| CG12679 | CG12679 |  |
| CG12689 | CG12689 |  |
| CG12991 | CG12991 |  |
| CG13124 | CG13124 |  |
| CG13306 | CG13306 |  |
| CG13349 | CG13349 |  |
| CG13603 | CG13603 |  |
| CG1387 | CG1387 |  |
| CG13920 | CG13920 |  |
| CG14215 | CG14215 |  |
| CG14613 | CG14613 |  |
| CG14619 | CG14619 |  |
| CG14701 | CG14701 |  |
| CG1486 | CG1486 |  |
| CG14963 | CG14963 |  |
| CG15098 | CG15098 |  |
| CG15147 | CG15147 |  |
| CG15173 | CG15173 |  |
| CG15220 | CG15220 |  |
| CG15260 | CG15260 |  |
| CG15452 | CG15452 |  |
| CG1550 | CG1550 |  |
| CG15828 | CG15828 |  |
| CG15873 | CG15873 |  |
| CG1637 | CG1637 |  |
| CG1657 | CG1657 |  |
| CG1703 | CG1703 |  |
| CG17259 | CG17259 |  |
| CG17278 | CG17278 |  |
| CG17294 | CG17294 |  |
| CG17666 | CG17666 |  |
| CG17739 | CG17739 |  |
| CG17776 | CG17776 |  |
| CG18011 | CG18011 |  |
| CG18367 | CG18367 |  |
| CG18596 | CG18596 |  |
| CG18616 | CG18616 |  |
| CG18641 | CG18641 |  |
| CG2453 | CG2453 |  |
| CG2533 | CG2533 |  |
| CG2656 | CG2656 |  |
| CG2658 | CG2658 |  |
| CG30269 | CG30269 |  |
| CG3198 | CG3198 |  |
| CG3224 | CG3224 |  |
| CG32495 | CG32495 |  |
| CG32528 | CG32528 |  |
| CG3295 | CG3295 |  |
| CG33057 | CG33057 |  |
| CG33487 | CG33487 |  |
| CG33491 | CG33491 |  |
| CG33496 | CG33496 |  |
| CG33498 | CG33498 |  |
| CG34383 | CG34383 |  |
| CG4017 | CG4017 |  |
| CG4038 | CG4038 |  |
| CG4198 | CG4198 |  |
| CG42347 | CG42347 |  |
| CG4404 | CG4404 |  |
| CG4409 | CG4409 |  |
| CG4461 | CG4461 |  |
| CG4764 | CG4764 |  |
| CG4858 | CG4858 |  |
| CG4880 | CG4880 |  |
| CG5168 | CG5168 |  |
| CG5391 | CG5391 |  |
| CG5767 | CG5767 |  |
| CG5823 | CG5823 |  |
| CG5830 | CG5830 |  |
| CG6385 | CG6385 |  |
| CG6415 | CG6415 |  |
| CG6686 | CG6686 |  |
| CG6704 | CG6704 |  |
| CG6719 | CG6719 |  |
| CG6723 | CG6723 |  |
| CG6762 | CG6762 |  |
| CG6763 | CG6763 |  |
| CG6769 | CG6769 |  |
| CG6770 | CG6770 |  |
| CG7332 | CG7332 |  |
| CG7488 | CG7488 |  |
| CG8010 | CG8010 |  |
| CG8145 | CG8145 |  |
| CG8209 | CG8209 |  |
| CG8360 | CG8360 |  |
| CG8736 | CG8736 |  |
| CG8740 | CG8740 |  |
| CG8786 | CG8786 |  |
| CG9987 | CG9987 |  |
| CG12690 | CHES-1-like | Checkpoint suppressor homologue |
| CG10618 | CHKov1 | CHKov1 |
| CR18854 | CR18854 |  |
| CG1753 | Cbs | Cystathionine beta-synthase |
| CG11847 | Clbn | Caliban |
| CG18066 | Cpr57A | Cuticular protein 57A |
| CG11525 | CycG | Cyclin G |
| CG12767 | Dip3 | Dorsal interacting protein 3 |
| CG12363 | Dlc90F | Dynein light chain 90F |
| CG43119 | Ect4 | Ect4 |
| CG6588 | Fas1 | Fasciclin 1 |
| CG2346 | Fmrf | FMRFamide-related |
| CG6835 | GS | Glutathione Synthetase |
| CG11086 | Gadd45 | Gadd45 |
| CG5469 | Gint3 | GDI interacting protein 3 |
| CG17530 | GstE6 | Glutathione S transferase E6 |
| CG1898 | HBS1 | HBS1 |
| CG14548 | HLHmbeta | E(spl) region transcript mbeta |
| CG5825 | His3.3A | Histone H3.3A |
| CG2903 | Hrs | Hepatocyte growth factor regulated tyrosine kinase substrate |
| CG6342 | Irp-1B | Iron regulatory protein 1B |
| CG1633 | Jafrac1 | thioredoxin peroxidase 1 |
| CG3767 | JhI-26 | Juvenile hormone-inducible protein 26 |
| CG4139 | Karl | Karl |
| CG5231 | Las | Lipoic acid synthase |
| CG12176 | Lig4 | Ligase4 |
| CG7614 | Mat1 | Mat1 |
| CG42309 | Mlp60A | Muscle LIM protein at 60A |
| CG4859 | Mmp1 | Matrix metalloproteinase 1 |
| CG34413 | NKAIN | Na,K-ATPase Interacting |
| CG13645 | Nmnat | Nicotinamide mononucleotide adenylyltransferase |
| CG1740 | Ntf-2 | Nuclear transport factor-2 |
| CG11856 | Nup358 | Nucleoporin 358 |
| CG7360 | Nup58 | Nucleoporin 58 |
| CG10198 | Nup98-96 | Nucleoporin 98-96 |
| CG8782 | Oat | Ornithine aminotransferase precursor |
| CG5472 | Pal2 | Peptidyl-alpha-hydroxyglycine-alpha-amidating lyase 2 |
| CG9358 | Phk-3 | Pherokine 3 |
| CG4268 | Pitslre | Pitslre |
| CG1954 | Pkc98E | Protein C kinase 98E |
| CG10371 | Plip | PTEN-like phosphatase |
| CG8073 | Pmm45A | Phosphomannomutase 45A |
| CG33103 | Ppn | Papilin |
| CG5266 | Pros25 | Proteasome 25kD subunit |
| CG4097 | Pros26 | Proteasome 26kD subunit |
| CG1519 | Prosalpha7 | Proteasome alpha7 subunit |
| CG8392 | Prosbeta1 | Proteasome beta1 subunit |
| CG11981 | Prosbeta3 | Proteasome beta3 subunit |
| CG12000 | Prosbeta7 | Proteasome beta7 subunit |
| CG1591 | REG | REG |
| CG9273 | RPA2 | Replication protein A2 |
| CG4212 | Rab14 | Rab-protein 14 |
| CG9862 | Rae1 | Rae1 |
| CG2849 | Rala | Ras-related protein |
| CG42236 | RanBPM | Ran-binding protein M |
| CG14213 | Rcd-1 | Required for cell differentiation 1 ortholog |
| CG8233 | Rcd1 | Reduction in Cnn dots 1 |
| CG14999 | RfC4 | Replication factor C subunit 4 |
| CG5192 | Rh6 | Rhodopsin 6 |
| CG8975 | RnrS | Ribonucleoside diphosphate reductase small subunit |
| CG3180 | RpII140 | RNA polymerase II 140kD subunit |
| CG8615 | RpL18 | Ribosomal protein L18 |
| CG5502 | RpL4 | Ribosomal protein L4 |
| CG4046 | RpS16 | Ribosomal protein S16 |
| CG2986 | RpS21 | Ribosomal protein S21 |
| CG10423 | RpS27 | Ribosomal protein S27 |
| CG11888 | Rpn2 | Rpn2 |
| CG42641 | Rpn3 | Regulatory particle non-ATPase 3 |
| CG16916 | Rpt3 | Rpt3 |
| CG3455 | Rpt4 | Rpt4 |
| CG1844 | SelG | Selenoprotein G |
| CG5352 | SmB | Small ribonucleoprotein particle protein SmB |
| CG10913 | Spn6 | Serine protease inhibitor 6 |
| CG4214 | Syx5 | Syntaxin 5 |
| CG4672 | TMS1 | TMS1 |
| CG32211 | Taf6 | TBP-associated factor 6 |
| CG8231 | Tcp-1zeta | T-cp1zeta |
| CG4898 | Tm1 | Tropomyosin 1 |
| CG7930 | TpnC73F | Troponin C at 73F |
| CG9998 | U2af50 | U2 small nuclear riboprotein auxiliary factor 50 |
| CG5486 | Ubp64E | Ubiquitin-specific protease 64E |
| CG4265 | Uch | Ubiquitin carboxy-terminal hydrolase |
| CG8310 | Vha36-3 | Vacuolar H[+] ATPase subunit 36-3 |
| CG3299 | Vinc | Vinculin |
| CG2179 | Xe7 | Xe7 |
| CG6898 | Zip3 | Zinc/iron regulated transporter-related protein 3 |
| CG8057 | alc | alicorn |
| CG1257 | alpha-Est3 | alpha-Esterase-3 |
| CG3717 | bcn92 | bcn92 |
| CG6223 | betaCop | beta-coatomer protein |
| CG7088 | bnb | bangles and beads |
| CG9424 | bocksbeutel | bocksbeutel |
| CG1676 | cactin | cactin |
| CG11024 | cl | clot |
| CG11949 | cora | coracle |
| CG14902 | decay | death executioner caspase related to Apopain/Yama |
| CG16969 | dgt2 | dim gamma-tubulin 2 |
| CG5452 | dnk | deoxyribonucleoside kinase |
| CG10161 | eIF-3p66 | Eukaryotic initiation factor 3 p66 subunit |
| CG7883 | eIF2B-alpha | eIF2B-alpha |
| CG8825 | gkt | glaikit |
| CG7570 | hale | hale-bopp |
| CG8019 | hay | haywire |
| CG10653 | hk | hook |
| CG11025 | isopeptidase-T-3 | isopeptidase-T-3 |
| CG11738 | l(1)G0004 | lethal (1) G0004 |
| CG2206 | l(1)G0193 | lethal (1) G0193 |
| CG4180 | l(2)35Bg | lethal (2) 35Bg |
| CG5931 | l(3)72Ab | lethal (3) 72Ab |
| CG13139 | lft | lowfat |
| CG5248 | loco | locomotion defects |
| CG3359 | mfas | midline fasciclin |
| CG7163 | mkg-p | monkey king protein |
| CG7012 | nct | nicastrin |
| CG10718 | neb | nebbish |
| CG33115 | nimB4 | nimrod B4 |
| CG16876 | nimC4 | nimrod C4 |
| CG7411 | ort | ora transientless |
| CG12085 | pUf68 | poly U binding factor 68kD |
| CG7228 | pes | peste |
| CG14472 | poe | purity of essence |
| CG7642 | ry | rosy |
| CG1664 | sbr | small bristles |
| CG5186 | slim | scruin like at the midline |
| CG7447 | slow | slowdown |
| CG1391 | sol | small optic lobes |
| CG14792 | sta | stubarista |
| CG5753 | stau | staufen |
| CG2194 | su(r) | suppressor of rudimentary |
| CG7925 | tko | technical knockout |
| CG10704 | toe | twin of eyg |
| CG8566 | unc-104 | unc-104 |
| CG4008 | und | uninitiated |
| CG4699 | wah | waharan |
| CG4448 | wda | will decrease acetylation |
| CG1454 | wdn | wings down |
| CG9089 | wus | wurst |
| CG18426 | ytr | yantar |

Irradiation down only

| Gene > Secondary Identifier | Gene > Symbol | Gene > Name |
| --- | --- | --- |
| CG6582 | Aac11 | Aac11 |
| CG9024 | Acp26Ab | Accessory gland-specific peptide 26Ab |
| CG12327 | Best3 | Bestrophin 3 |
| CG6500 | Bx | Beadex |
| CG10104 | CG10104 |  |
| CG10550 | CG10550 |  |
| CG10651 | CG10651 |  |
| CG11253 | CG11253 |  |
| CG11455 | CG11455 |  |
| CG12895 | CG12895 |  |
| CG13405 | CG13405 |  |
| CG13471 | CG13471 |  |
| CG13481 | CG13481 |  |
| CG13597 | CG13597 |  |
| CG13843 | CG13843 |  |
| CG13947 | CG13947 |  |
| CG14383 | CG14383 |  |
| CG14443 | CG14443 |  |
| CG14684 | CG14684 |  |
| CG14687 | CG14687 |  |
| CG14817 | CG14817 |  |
| CG15023 | CG15023 |  |
| CG1545 | CG1545 |  |
| CG16727 | CG16727 |  |
| CG16775 | CG16775 |  |
| CG1732 | CG1732 |  |
| CG17625 | CG17625 |  |
| CG18109 | CG18109 |  |
| CG18135 | CG18135 |  |
| CG18136 | CG18136 |  |
| CG18173 | CG18173 |  |
| CG18281 | CG18281 |  |
| CG18598 | CG18598 |  |
| CG1908 | CG1908 |  |
| CG30281 | CG30281 |  |
| CG30460 | CG30460 |  |
| CG31674 | CG31674 |  |
| CG31752 | CG31752 |  |
| CG31958 | CG31958 |  |
| CG31960 | CG31960 |  |
| CG32043 | CG32043 |  |
| CG32087 | CG32087 |  |
| CG32633 | CG32633 |  |
| CG32736 | CG32736 |  |
| CG3285 | CG3285 |  |
| CG33287 | CG33287 |  |
| CG3690 | CG3690 |  |
| CG3719 | CG3719 |  |
| CG42613 | CG42613 |  |
| CG4329 | CG4329 |  |
| CG4468 | CG4468 |  |
| CG4572 | CG4572 |  |
| CG4653 | CG4653 |  |
| CG5261 | CG5261 |  |
| CG5681 | CG5681 |  |
| CG5770 | CG5770 |  |
| CG6834 | CG6834 |  |
| CG7135 | CG7135 |  |
| CG7295 | CG7295 |  |
| CG7381 | CG7381 |  |
| CG7433 | CG7433 |  |
| CG7634 | CG7634 |  |
| CG7813 | CG7813 |  |
| CG7881 | CG7881 |  |
| CG7907 | CG7907 |  |
| CG8176 | CG8176 |  |
| CG9171 | CG9171 |  |
| CG9360 | CG9360 |  |
| CG9507 | CG9507 |  |
| CG9568 | CG9568 |  |
| CG9896 | CG9896 |  |
| CR13656 | CR13656 |  |
| CG7962 | CdsA | CDP diglyceride synthetase |
| CG15033 | CheA7a | Chemosensory protein A 7a |
| CG8050 | Cys | Cystatin-like |
| CG7266 | Eip71CD | Ecdysone-induced protein 28/29kD |
| CG9461 | FBX011 | FBX011 ortholog |
| CG10763 | Gbeta5 | Gbeta5 |
| CG3694 | Ggamma30A | G protein gamma30A |
| CG1743 | Gs2 | Glutamine synthetase 2 |
| CG6964 | Gug | Grunge |
| CG5460 | H | Hairless |
| CG9983 | Hrb98DE | Heterogeneous nuclear ribonucleoprotein at 98DE |
| CG4466 | Hsp27 | Heat shock protein 27 |
| CG14934 | Mal-B1 | Maltase B1 |
| CG5784 | Mapmodulin | Mapmodulin |
| CG12092 | Npc1b | Niemann-Pick type C-1b |
| CG3382 | Oatp58Db | Organic anion transporting polypeptide 58Db |
| CG11390 | PebIII | Ejaculatory bulb protein III |
| CG4710 | Pino | Pinocchio |
| CG9441 | Pu | Punch |
| CG7808 | RpS8 | Ribosomal protein S8 |
| CG8174 | SRPK | SRPK |
| CG4257 | Stat92E | Signal-transducer and activator of transcription protein at 92E |
| CG5878 | alpha4GT2 | alpha4GT2 |
| CG11910 | alrm | astrocytic leucine-rich repeat molecule |
| CG32601 | betaNACtes3 | |
| CG32598 | betaNACtes6 | |
| CG10630 | blanks | blanks |
| CG12478 | bru-3 | bruno-3 |
| CG9472 | brv1 | brivido-1 |
| CG43122 | cic | capicua |
| CG9075 | eIF-4a | Eukaryotic initiation factor 4a |
| CG9659 | egh | egghead |
| CG4568 | fzo | fuzzy onions |
| CG17117 | hth | homothorax |
| CG12199 | kek5 | kekkon5 |
| CG17046 | klar | klarsicht |
| CG1639 | l(1)10Bb | lethal (1) 10Bb |
| CG3920 | l(2)k16918 | lethal (2) k16918 |
| CG2826 | lectin-21Ca | lectin-21Ca |
| CG8470 | mRpS30 | mitochondrial ribosomal protein S30 |
| CG12218 | mei-P26 | mei-P26 |
| CG7437 | mub | mushroom-body expressed |
| CG1560 | mys | myospheroid |
| CG10722 | nesd | nessun dorma |
| CG9347 | ninaB | neither inactivation nor afterpotential B |
| CG7892 | nmo | nemo |
| CG9258 | nrv1 | nervana 1 |
| CG9261 | nrv2 | nervana 2 |
| CG3875 | nsr | novel spermatogenesis regulator |
| CG5052 | pim | pimples |
| CG42612 | plx | pollux |
| CG17228 | pros | prospero |
| CG10233 | rtp | retinophilin |
| CG10693 | slo | slowpoke |
| CG1915 | sls | sallimus |
| CG32383 | sphinx1 | sphinx1 |
| CG3056 | ssx | sister-of-Sex-lethal |
| CG3533 | uzip | unzipped |
| CG32754 | vanin-like | vanin-like |
| CG13549 | yip3 | yippee interacting protein 3 |
