# Supplementary material for: Gene expression changes in response to aging compared to heat stress, oxidative stress and ionizing radiation in Drosophila melanogaster
Source: Aging (Albany NY). 2012 Nov 30;4(11):768–89. doi: 10.18632/aging.100499 (PMC3560439; doi:10.18632/aging.100499)
Supplement: Supplementary file 3 [file aging-04-768-s003.docx]

Supplemental Table S2. Gene expression changes shared by aging and individual stresses (sugar excluded)

Age up and O2 up

| Gene > Secondary Identifier | Gene > Symbol | Gene > Name |
| --- | --- | --- |
| CG12876 | ALiX | ALG-2 interacting protein X |
| CG3821 | Aats-asp | Aspartyl-tRNA synthetase |
| CG9621 | Adgf-D | Adenosine deaminase-related growth factor D |
| CG11654 | Ahcy13 | Adenosylhomocysteinase at 13 |
| CG5730 | AnnIX | Annexin IX |
| CG13941 | Arc2 | Arc2 |
| CG10146 | AttA | Attacin-A |
| CG18372 | AttB | Attacin-B |
| CG10103 | CG10103 |  |
| CG10126 | CG10126 |  |
| CG10621 | CG10621 |  |
| CG10638 | CG10638 |  |
| CG10912 | CG10912 |  |
| CG10916 | CG10916 |  |
| CG11030 | CG11030 |  |
| CG11089 | CG11089 |  |
| CG11893 | CG11893 |  |
| CG11899 | CG11899 |  |
| CG12224 | CG12224 |  |
| CG12310 | CG12310 |  |
| CG12780 | CG12780 |  |
| CG12868 | CG12868 |  |
| CG12877 | CG12877 |  |
| CG13117 | CG13117 |  |
| CG13887 | CG13887 |  |
| CG13905 | CG13905 |  |
| CG13947 | CG13947 |  |
| CG14109 | CG14109 |  |
| CG14207 | CG14207 |  |
| CG14245 | CG14245 |  |
| CG14246 | CG14246 |  |
| CG14907 | CG14907 |  |
| CG14933 | CG14933 |  |
| CG15043 | CG15043 |  |
| CG15065 | CG15065 |  |
| CG1572 | CG1572 |  |
| CG15784 | CG15784 |  |
| CG16978 | CG16978 |  |
| CG17904 | CG17904 |  |
| CG18067 | CG18067 |  |
| CG2064 | CG2064 |  |
| CG2065 | CG2065 |  |
| CG2217 | CG2217 |  |
| CG2909 | CG2909 |  |
| CG3008 | CG3008 |  |
| CG3011 | CG3011 |  |
| CG3036 | CG3036 |  |
| CG31638 | CG31638 |  |
| CG31793 | CG31793 |  |
| CG32103 | CG32103 |  |
| CG32549 | CG32549 |  |
| CG33229 | CG33229 |  |
| CG3448 | CG3448 |  |
| CG3505 | CG3505 |  |
| CG3590 | CG3590 |  |
| CG4269 | CG4269 |  |
| CG43427 | CG43427 |  |
| CG4500 | CG4500 |  |
| CG5224 | CG5224 |  |
| CG5290 | CG5290 |  |
| CG5493 | CG5493 |  |
| CG5778 | CG5778 |  |
| CG5869 | CG5869 |  |
| CG5955 | CG5955 |  |
| CG5966 | CG5966 |  |
| CG6188 | CG6188 |  |
| CG6272 | CG6272 |  |
| CG6353 | CG6353 |  |
| CG6767 | CG6767 |  |
| CG7033 | CG7033 |  |
| CG7130 | CG7130 |  |
| CG7192 | CG7192 |  |
| CG8112 | CG8112 |  |
| CG8206 | CG8206 |  |
| CG8678 | CG8678 |  |
| CG9547 | CG9547 |  |
| CG9689 | CG9689 |  |
| CG9989 | CG9989 |  |
| CG4618 | CHMP2B | Charged multivesicular body protein 2b |
| CG8107 | CalpB | Calpain-B |
| CG8439 | Cct5 | T-complex Chaperonin 5 |
| CG1878 | CecB | Cecropin B |
| CG3642 | Clp | Clipper |
| CG8864 | Cyp28a5 | Cyp28a5 |
| CG9964 | Cyp309a1 | Cyp309a1 |
| CG10842 | Cyp4p1 | Cytochrome P450-4p1 |
| CG10248 | Cyp6a8 | Cytochrome P450-6a8 |
| CG5729 | Dgp-1 | Dgp-1 |
| CG10578 | DnaJ-1 | DnaJ-like-1 |
| CG10810 | Drs | Drosomycin |
| CG4930 | EndoGI | Endonuclease G inhibitor |
| CG2216 | Fer1HCH | Ferritin 1 heavy chain homologue |
| CG9434 | Fst | Frost |
| CG2718 | Gs1 | Glutamine synthetase 1 |
| CG4181 | GstD2 | Glutathione S transferase D2 |
| CG12242 | GstD5 | Glutathione S transferase D5 |
| CG5164 | GstE1 | Glutathione S transferase E1 |
| CG17531 | GstE7 | Glutathione S transferase E7 |
| CG17533 | GstE8 | Glutathione S transferase E8 |
| CG4460 | Hsp22 | Heat shock protein 22 |
| CG4463 | Hsp23 | Heat shock protein 23 |
| CG4183 | Hsp26 | Heat shock protein 26 |
| CG4456 | Hsp67Bb | Heat shock gene 67Bb |
| CG5436 | Hsp68 | Heat shock protein 68 |
| CG18743 | Hsp70Ab | Heat-shock-protein-70Ab |
| CG31449 | Hsp70Ba | Heat-shock-protein-70Ba |
| CG31359 | Hsp70Bb | Heat-shock-protein-70Bb |
| CG5834 | Hsp70Bbb | Hsp70Bbb |
| CG6489 | Hsp70Bc | Heat-shock-protein-70Bc |
| CG1242 | Hsp83 | Heat shock protein 83 |
| CG18108 | IM1 | Immune induced molecule 1 |
| CG18106 | IM2 | Immune induced molecule 2 |
| CG15066 | IM23 | Immune induced molecule 23 |
| CG16844 | IM3 | Immune induced molecule 3 |
| CG4472 | Idgf1 | Imaginal disc growth factor 1 |
| CG10160 | ImpL3 | Ecdysone-inducible gene L3 |
| CG5247 | Irbp | Inverted repeat-binding protein |
| CG8913 | Irc | Immune-regulated catalase |
| CG10922 | La | La autoantigen-like |
| CG3743 | MTF-1 | Metal response element-binding Transcription Factor-1 |
| CG14935 | Mal-B2 | Maltase B2 |
| CG9470 | MtnA | Metallothionein A |
| CG2330 | Neurochondrin | Neurochondrin |
| CG3798 | Nmda1 | N-methyl-D-aspartate receptor-associated protein |
| CG18466 | Nmdmc | NAD-dependent methylenetetrahydrofolate dehydrogenase |
| CG17725 | Pepck | Phosphoenolpyruvate carboxykinase |
| CG31864 | Qtzl | Quetzalcoatl |
| CG34374 | Rapgap1 | Rapgap1 |
| CG11992 | Rel | Relish |
| CG5371 | RnrL | Ribonucleoside diphosphate reductase large subunit |
| CG9633 | RpA-70 | Replication Protein A 70 |
| CG2471 | Sclp | Sclp |
| CG3926 | Spat | Serine pyruvate aminotransferase |
| CG11331 | Spn27A | Serpin 27A |
| CG7219 | Spn28D | Serpin 28D |
| CG6687 | Spn88Eb | Serpin 88Eb |
| CG4817 | Ssrp | Structure specific recognition protein |
| CG5374 | T-cp1 | Tcp1-like |
| CG14027 | TotM | Turandot M |
| CG12846 | Tsp42Ed | Tetraspanin 42Ed |
| CG6120 | Tsp96F | Tetraspanin 96F |
| CG17836 | Xrp1 |  |
| CG3705 | aay | astray |
| CG9127 | ade2 | adenosine 2 |
| CG31628 | ade3 | adenosine 3 |
| CG3989 | ade5 | ade5 |
| CG7380 | baf | barrier to autointegration factor |
| CG5295 | bmm | brummer |
| CG12132 | c11.1 | c11.1 |
| CG10460 | cer | crammer |
| CG9553 | chic | chickadee |
| CG17100 | cwo | clockwork orange |
| CG10798 | dm | diminutive |
| CG1044 | dos | daughter of sevenless |
| CG4153 | eIF-2beta | Eukaryotic initiation factor 2beta |
| CG5202 | escl | escl |
| CG9734 | glob1 | globin 1 |
| CG4533 | l(2)efl | lethal (2) essential for life |
| CG7850 | puc | puckered |
| CG4067 | pug | pugilist |
| CG10360 | ref(2)P | refractory to sigma P |
| CG12292 | spict | spichthyin |
| CG32130 | stv | starvin |
| CG11844 | vig2 | vig2 |
| CG31764 | vir-1 | virus-induced RNA 1 |
| CG42365 |  |  |
| CG42807 |  |  |

Age down O2 down

| Gene > Secondary Identifier | Gene > Symbol | Gene > Name |
| --- | --- | --- |
| CG10593 | Acer | Angiotensin-converting enzyme-related |
| CG8982 | Acp26Aa | Accessory gland-specific peptide 26Aa |
| CG7157 | Acp36DE | Accessory gland peptide 36DE |
| CG1262 | Acp62F | Accessory gland peptide 62F |
| CG3801 | Acp76A | Accessory gland-specific peptide 76A |
| CG9540 | Ag5r2 | Antigen 5-related 2 |
| CG11140 | Aldh-III | Aldehyde dehydrogenase type III |
| CG1462 | Aph-4 | Alkaline phosphatase 4 |
| CG5711 | Arr1 | Arrestin 1 |
| CG6378 | BM-40-SPARC | BM-40-SPARC |
| CG13095 | Bace | beta-site APP-cleaving enzyme |
| CG10026 | CG10026 |  |
| CG10116 | CG10116 |  |
| CG10237 | CG10237 |  |
| CG10340 | CG10340 |  |
| CG10467 | CG10467 |  |
| CG10472 | CG10472 |  |
| CG10477 | CG10477 |  |
| CG10513 | CG10513 |  |
| CG10514 | CG10514 |  |
| CG10587 | CG10587 |  |
| CG10592 | CG10592 |  |
| CG10657 | CG10657 |  |
| CG10827 | CG10827 |  |
| CG10866 | CG10866 |  |
| CG11112 | CG11112 |  |
| CG11200 | CG11200 | Carbonyl reductase |
| CG11378 | CG11378 |  |
| CG11598 | CG11598 |  |
| CG11752 | CG11752 |  |
| CG11892 | CG11892 |  |
| CG11911 | CG11911 |  |
| CG11912 | CG11912 |  |
| CG12057 | CG12057 |  |
| CG12079 | CG12079 |  |
| CG12203 | CG12203 |  |
| CG12374 | CG12374 |  |
| CG12990 | CG12990 |  |
| CG13091 | CG13091 |  |
| CG13101 | CG13101 |  |
| CG13155 | CG13155 |  |
| CG13255 | CG13255 |  |
| CG13492 | CG13492 |  |
| CG13607 | CG13607 |  |
| CG13833 | CG13833 |  |
| CG14034 | CG14034 |  |
| CG14072 | CG14072 |  |
| CG14120 | CG14120 |  |
| CG14141 | CG14141 |  |
| CG14629 | CG14629 |  |
| CG14661 | CG14661 |  |
| CG15254 | CG15254 |  |
| CG15255 | CG15255 |  |
| CG15353 | CG15353 |  |
| CG15635 | CG15635 |  |
| CG1648 | CG1648 |  |
| CG16749 | CG16749 |  |
| CG17029 | CG17029 |  |
| CG17032 | CG17032 |  |
| CG17097 | CG17097 |  |
| CG17192 | CG17192 |  |
| CG17472 | CG17472 |  |
| CG17571 | CG17571 |  |
| CG17633 | CG17633 |  |
| CG1809 | CG1809 |  |
| CG18179 | CG18179 |  |
| CG18180 | CG18180 |  |
| CG18284 | CG18284 |  |
| CG18301 | CG18301 |  |
| CG18302 | CG18302 |  |
| CG18327 | CG18327 |  |
| CG18493 | CG18493 |  |
| CG18585 | CG18585 |  |
| CG18624 | CG18624 |  |
| CG1946 | CG1946 |  |
| CG2070 | CG2070 |  |
| CG2663 | CG2663 |  |
| CG2930 | CG2930 |  |
| CG30025 | CG30025 |  |
| CG30031 | CG30031 |  |
| CG30427 | CG30427 |  |
| CG3106 | CG3106 |  |
| CG31086 | CG31086 |  |
| CG31148 | CG31148 |  |
| CG31233 | CG31233 |  |
| CG31265 | CG31265 |  |
| CG31872 | CG31872 |  |
| CG31883 | CG31883 |  |
| CG3192 | CG3192 |  |
| CG32444 | CG32444 |  |
| CG3290 | CG3290 |  |
| CG3301 | CG3301 |  |
| CG33259 | CG33259 |  |
| CG3560 | CG3560 |  |
| CG3734 | CG3734 |  |
| CG3739 | CG3739 |  |
| CG3940 | CG3940 |  |
| CG4000 | CG4000 |  |
| CG42235 | CG42235 |  |
| CG42351 | CG42351 |  |
| CG4462 | CG4462 |  |
| CG4729 | CG4729 |  |
| CG4734 | CG4734 |  |
| CG4753 | CG4753 |  |
| CG4847 | CG4847 |  |
| CG5023 | CG5023 |  |
| CG5107 | CG5107 |  |
| CG5150 | CG5150 |  |
| CG5162 | CG5162 |  |
| CG5618 | CG5618 |  |
| CG5804 | CG5804 |  |
| CG5932 | CG5932 |  |
| CG5945 | CG5945 |  |
| CG5991 | CG5991 |  |
| CG6293 | CG6293 |  |
| CG6295 | CG6295 |  |
| CG6660 | CG6660 |  |
| CG6726 | CG6726 |  |
| CG6733 | CG6733 |  |
| CG6839 | CG6839 |  |
| CG7542 | CG7542 |  |
| CG7916 | CG7916 |  |
| CG7953 | CG7953 |  |
| CG8093 | CG8093 |  |
| CG8147 | CG8147 |  |
| CG8420 | CG8420 |  |
| CG8560 | CG8560 |  |
| CG8562 | CG8562 |  |
| CG8628 | CG8628 |  |
| CG8661 | CG8661 |  |
| CG8708 | CG8708 |  |
| CG8745 | CG8745 |  |
| CG8773 | CG8773 |  |
| CG8834 | CG8834 |  |
| CG8907 | CG8907 |  |
| CG8997 | CG8997 |  |
| CG9259 | CG9259 |  |
| CG9306 | CG9306 |  |
| CG9399 | CG9399 |  |
| CG9463 | CG9463 |  |
| CG9465 | CG9465 |  |
| CG9466 | CG9466 |  |
| CG9468 | CG9468 |  |
| CG9497 | CG9497 |  |
| CG9673 | CG9673 |  |
| CG9682 | CG9682 |  |
| CG30042 | Cpr49Ab | Cuticular protein 49Ab |
| CG8505 | Cpr49Ae | Cuticular protein 49Ae |
| CG15077 | Cyp12b2 | Cyp12b2 |
| CG5137 | Cyp312a1 | Cyp312a1 |
| CG3360 | Cyp313a1 | Cyp313a1 |
| CG8302 | Cyp4aa1 | Cyp4aa1 |
| CG3540 | Cyp4d14 | Cyp4d14 |
| CG6730 | Cyp4d21 | Cyp4d21 |
| CG2062 | Cyp4e1 | Cytochrome P450-4e1 |
| CG4654 | Dp | DP transcription factor |
| CG32072 | Elo68alpha | Elongase 68alpha |
| CG9092 | Gal | beta galactosidase |
| CG9042 | Gpdh | Glycerol 3 phosphate dehydrogenase |
| CG43388 | Hk | Hyperkinetic |
| CG8464 | HtrA2 | HtrA2 |
| CG8869 | Jon25Bii | Jonah 25Bii |
| CG8871 | Jon25Biii | Jonah 25Biii |
| CG8579 | Jon44E | Jonah 44E |
| CG10475 | Jon65Ai | Jonah 65Ai |
| CG6580 | Jon65Aii | Jonah 65Aii |
| CG6483 | Jon65Aiii | Jonah 65Aiii |
| CG6467 | Jon65Aiv | Jonah 65Aiv |
| CG6298 | Jon74E | Jonah 74E |
| CG31034 | Jon99Cii | Jonah 99Cii |
| CG31362 | Jon99Ciii | Jonah 99Ciii |
| CG18030 | Jon99Fi | Jonah 99Fi |
| CG2229 | Jon99Fii | Jonah 99Fii |
| CG4178 | Lsp1beta | Larval serum protein 1 beta |
| CG1165 | LysS | Lysozyme S |
| CG8695 | Mal-A3 | Maltase A3 |
| CG11669 | Mal-A7 | Maltase A7 |
| CG13431 | Mgat1 | UDP-GlcNAc:a-3-D-mannoside-beta-1,2-N-acetylglucosaminyltransferase I |
| CG4123 | Mipp1 | Multiple inositol polyphosphate phosphatase 1 |
| CG9074 | Mst57Da | Male-specific RNA 57Da |
| CG5016 | Mst57Db | Male-specific RNA 57Db |
| CG4986 | Mst57Dc | Male-specific RNA 57Dc |
| CG6004 | Muc68D | Mucin 68D |
| CG12813 | Npc2d | Niemann-Pick type C-2d |
| CG6164 | Npc2f | Niemann-Pick type C-2f |
| CG13061 | Nplp3 | Neuropeptide-like precursor 3 |
| CG7592 | Obp99b | Odorant-binding protein 99b |
| CG14745 | PGRP-SC2 | PGRP-SC2 |
| CG31794 | Pax | Paxillin |
| CG2668 | Peb | Protein ejaculatory bulb |
| CG2665 | PebII | Protein ejaculatory bulb II |
| CG18594 | Pebp1 | Phosphatidylethanolamine-binding protein 1 |
| CG11064 | Rfabg | Retinoid- and fatty acid-binding glycoprotein |
| CG4812 | Ser8 | Ser8 |
| CG8137 | Spn2 | Serine protease inhibitor 2 |
| CG6663 | Spn77Bb | Serpin 77Bb |
| CG18255 | Strn-Mlck | Stretchin-Mlck |
| CG9496 | Tsp29Fb | Tetraspanin 29Fb |
| CG6649 | Ugt35b | UDP-glycosyltransferase 35b |
| CG7678 | Vha100-4 | Vacuolar H[+] ATPase subunit 100-4 |
| CG1121 | alpha-Est8 | alpha-Esterase-8 |
| CG18104 | arg | arginase |
| CG18211 | betaTry | betaTrypsin |
| CG33555 | btsz | bitesize |
| CG12351 | deltaTry | deltaTrypsin |
| CG18681 | epsilonTry | epsilonTrypsin |
| CG6783 | fabp | fatty acid bindin protein |
| CG30028 | gammaTry | gammaTrypsin |
| CG7754 | iotaTry | iotaTrypsin |
| CG12388 | kappaTry | kappaTry |
| CG3861 | kdn | knockdown |
| CG7532 | l(2)34Fc | lethal (2) 34Fc |
| CG13240 | l(2)35Di | lethal (2) 35Di |
| CG12350 | lambdaTry | lambdaTry |
| CG16834 | lectin-33A | lectin-33A |
| CG1803 | regucalcin | regucalcin |
| CG9675 | spheroide | spheroide |
| CG17976 | sut3 | sugar transporter 3 |
| CG4979 | sxe2 | sex-specific enzyme 2 |
| CG12385 | thetaTry | thetaTrypsin |
| CG5269 | vib | vibrator |
| CG6457 | yip7 | yippee interacting protein 7 |

Age up and H2O2 up

| Gene > Secondary Identifier | Gene > Symbol | Gene > Name |
| --- | --- | --- |
| CG12876 | ALiX | ALG-2 interacting protein X |
| CG3821 | Aats-asp | Aspartyl-tRNA synthetase |
| CG11654 | Ahcy13 | Adenosylhomocysteinase at 13 |
| CG5730 | AnnIX | Annexin IX |
| CG13941 | Arc2 | Arc2 |
| CG10146 | AttA | Attacin-A |
| CG18372 | AttB | Attacin-B |
| CG10103 | CG10103 |  |
| CG10126 | CG10126 |  |
| CG10641 | CG10641 |  |
| CG10916 | CG10916 |  |
| CG11009 | CG11009 |  |
| CG11030 | CG11030 |  |
| CG11089 | CG11089 |  |
| CG11686 | CG11686 |  |
| CG11880 | CG11880 |  |
| CG11899 | CG11899 |  |
| CG12012 | CG12012 |  |
| CG12112 | CG12112 |  |
| CG12896 | CG12896 |  |
| CG13117 | CG13117 |  |
| CG13323 | CG13323 |  |
| CG13324 | CG13324 |  |
| CG13905 | CG13905 |  |
| CG13947 | CG13947 |  |
| CG14245 | CG14245 |  |
| CG14246 | CG14246 |  |
| CG14401 | CG14401 |  |
| CG14906 | CG14906 |  |
| CG14907 | CG14907 |  |
| CG15043 | CG15043 |  |
| CG15065 | CG15065 |  |
| CG15784 | CG15784 |  |
| CG1943 | CG1943 |  |
| CG2064 | CG2064 |  |
| CG2201 | CG2201 |  |
| CG2909 | CG2909 |  |
| CG3008 | CG3008 |  |
| CG3011 | CG3011 |  |
| CG3036 | CG3036 |  |
| CG31638 | CG31638 |  |
| CG31705 | CG31705 |  |
| CG32103 | CG32103 |  |
| CG33056 | CG33056 |  |
| CG33229 | CG33229 |  |
| CG3590 | CG3590 |  |
| CG3999 | CG3999 |  |
| CG4199 | CG4199 |  |
| CG4269 | CG4269 |  |
| CG5346 | CG5346 |  |
| CG5493 | CG5493 |  |
| CG5869 | CG5869 |  |
| CG5955 | CG5955 |  |
| CG5966 | CG5966 |  |
| CG6188 | CG6188 |  |
| CG6353 | CG6353 |  |
| CG6767 | CG6767 |  |
| CG7192 | CG7192 |  |
| CG7267 | CG7267 |  |
| CG8112 | CG8112 |  |
| CG8206 | CG8206 |  |
| CG8317 | CG8317 |  |
| CG8678 | CG8678 |  |
| CG8791 | CG8791 |  |
| CG9336 | CG9336 |  |
| CG9547 | CG9547 |  |
| CG9989 | CG9989 |  |
| CG4618 | CHMP2B | Charged multivesicular body protein 2b |
| CG8107 | CalpB | Calpain-B |
| CG1373 | CecC | Cecropin C |
| CG8864 | Cyp28a5 | Cyp28a5 |
| CG10842 | Cyp4p1 | Cytochrome P450-4p1 |
| CG10248 | Cyp6a8 | Cytochrome P450-6a8 |
| CG10246 | Cyp6a9 | Cytochrome P450-6a9 |
| CG18188 | Damm | Death associated molecule related to Mch2 |
| CG10578 | DnaJ-1 | DnaJ-like-1 |
| CG2718 | Gs1 | Glutamine synthetase 1 |
| CG4181 | GstD2 | Glutathione S transferase D2 |
| CG12242 | GstD5 | Glutathione S transferase D5 |
| CG7399 | Hn | Henna |
| CG4183 | Hsp26 | Heat shock protein 26 |
| CG5436 | Hsp68 | Heat shock protein 68 |
| CG18743 | Hsp70Ab | Heat-shock-protein-70Ab |
| CG31449 | Hsp70Ba | Heat-shock-protein-70Ba |
| CG31359 | Hsp70Bb | Heat-shock-protein-70Bb |
| CG5834 | Hsp70Bbb | Hsp70Bbb |
| CG6489 | Hsp70Bc | Heat-shock-protein-70Bc |
| CG1242 | Hsp83 | Heat shock protein 83 |
| CG4472 | Idgf1 | Imaginal disc growth factor 1 |
| CG10160 | ImpL3 | Ecdysone-inducible gene L3 |
| CG8913 | Irc | Immune-regulated catalase |
| CG9080 | Listericin | Listericin |
| CG9120 | LysX | Lysozyme X |
| CG18362 | Mio | Mlx interactor |
| CG7438 | Myo31DF | Myosin 31DF |
| CG18466 | Nmdmc | NAD-dependent methylenetetrahydrofolate dehydrogenase |
| CG17725 | Pepck | Phosphoenolpyruvate carboxykinase |
| CG12405 | Prx2540-1 | Peroxiredoxin 2540-1 |
| CG11765 | Prx2540-2 | Peroxiredoxin 2540-2 |
| CG11992 | Rel | Relish |
| CG8282 | Snx6 | Snx6 |
| CG3926 | Spat | Serine pyruvate aminotransferase |
| CG11331 | Spn27A | Serpin 27A |
| CG7219 | Spn28D | Serpin 28D |
| CG6687 | Spn88Eb | Serpin 88Eb |
| CG14027 | TotM | Turandot M |
| CG12846 | Tsp42Ed | Tetraspanin 42Ed |
| CG7171 | Uro | Urate oxidase |
| CG17836 | Xrp1 |  |
| CG3705 | aay | astray |
| CG9127 | ade2 | adenosine 2 |
| CG31628 | ade3 | adenosine 3 |
| CG3989 | ade5 | ade5 |
| CG12132 | c11.1 | c11.1 |
| CG5848 | cact | cactus |
| CG10460 | cer | crammer |
| CG7207 | cert | ceramide transfer protein |
| CG4944 | cib | ciboulot |
| CG10540 | cpa | capping protein alpha |
| CG10798 | dm | diminutive |
| CG1044 | dos | daughter of sevenless |
| CG3365 | drongo | drongo |
| CG2086 | drpr | draper |
| CG6575 | glec | gliolectin |
| CG9734 | glob1 | globin 1 |
| CG1487 | krz | kurtz |
| CG4533 | l(2)efl | lethal (2) essential for life |
| CG7850 | puc | puckered |
| CG6339 | rad50 | rad50 |
| CG10360 | ref(2)P | refractory to sigma P |
| CG3595 | sqh | spaghetti squash |
| CG32130 | stv | starvin |
| CG31764 | vir-1 | virus-induced RNA 1 |
| CG42365 |  |  |

Age down H2O2 down

| Gene > Secondary Identifier | Gene > Symbol | Gene > Name |
| --- | --- | --- |
| CG8896 | 18w | 18 wheeler |
| CG8189 | ATPsyn-b | ATP synthase, subunit b |
| CG10593 | Acer | Angiotensin-converting enzyme-related |
| CG11140 | Aldh-III | Aldehyde dehydrogenase type III |
| CG1462 | Aph-4 | Alkaline phosphatase 4 |
| CG6378 | BM-40-SPARC | BM-40-SPARC |
| CG12487 | BobA | Brother of Bearded A |
| CG10026 | CG10026 |  |
| CG10345 | CG10345 |  |
| CG10467 | CG10467 |  |
| CG10587 | CG10587 |  |
| CG10592 | CG10592 |  |
| CG10827 | CG10827 |  |
| CG11112 | CG11112 |  |
| CG11474 | CG11474 |  |
| CG11752 | CG11752 |  |
| CG11912 | CG11912 |  |
| CG12105 | CG12105 |  |
| CG12158 | CG12158 |  |
| CG12203 | CG12203 |  |
| CG13091 | CG13091 |  |
| CG13465 | CG13465 |  |
| CG13492 | CG13492 |  |
| CG13607 | CG13607 |  |
| CG13833 | CG13833 |  |
| CG14072 | CG14072 |  |
| CG14120 | CG14120 |  |
| CG1444 | CG1444 |  |
| CG14482 | CG14482 |  |
| CG14629 | CG14629 |  |
| CG14661 | CG14661 |  |
| CG14712 | CG14712 |  |
| CG15534 | CG15534 |  |
| CG15841 | CG15841 |  |
| CG1648 | CG1648 |  |
| CG16904 | CG16904 |  |
| CG17029 | CG17029 |  |
| CG17032 | CG17032 |  |
| CG1809 | CG1809 |  |
| CG18302 | CG18302 |  |
| CG18493 | CG18493 |  |
| CG18585 | CG18585 |  |
| CG2930 | CG2930 |  |
| CG30427 | CG30427 |  |
| CG3106 | CG3106 |  |
| CG31145 | CG31145 |  |
| CG31148 | CG31148 |  |
| CG31883 | CG31883 |  |
| CG3290 | CG3290 |  |
| CG3301 | CG3301 |  |
| CG33523 | CG33523 |  |
| CG3683 | CG3683 |  |
| CG3734 | CG3734 |  |
| CG3739 | CG3739 |  |
| CG3823 | CG3823 |  |
| CG3940 | CG3940 |  |
| CG4000 | CG4000 |  |
| CG4053 | CG4053 |  |
| CG42235 | CG42235 |  |
| CG4847 | CG4847 |  |
| CG5037 | CG5037 |  |
| CG5107 | CG5107 |  |
| CG5150 | CG5150 |  |
| CG5561 | CG5561 |  |
| CG5618 | CG5618 |  |
| CG5804 | CG5804 |  |
| CG5903 | CG5903 |  |
| CG5991 | CG5991 |  |
| CG6463 | CG6463 |  |
| CG6555 | CG6555 |  |
| CG6660 | CG6660 |  |
| CG6726 | CG6726 |  |
| CG6733 | CG6733 |  |
| CG7430 | CG7430 |  |
| CG7580 | CG7580 |  |
| CG8093 | CG8093 |  |
| CG8147 | CG8147 |  |
| CG8329 | CG8329 |  |
| CG8560 | CG8560 |  |
| CG8562 | CG8562 |  |
| CG8708 | CG8708 |  |
| CG8773 | CG8773 |  |
| CG9463 | CG9463 |  |
| CG9466 | CG9466 |  |
| CG9468 | CG9468 |  |
| CG9497 | CG9497 |  |
| CG9682 | CG9682 |  |
| CG8505 | Cpr49Ae | Cuticular protein 49Ae |
| CG8733 | Cyp305a1 | Cyp305a1 |
| CG5137 | Cyp312a1 | Cyp312a1 |
| CG3360 | Cyp313a1 | Cyp313a1 |
| CG8302 | Cyp4aa1 | Cyp4aa1 |
| CG3540 | Cyp4d14 | Cyp4d14 |
| CG2062 | Cyp4e1 | Cytochrome P450-4e1 |
| CG6050 | EfTuM | Elongation factor Tu mitochondrial |
| CG32072 | Elo68alpha | Elongase 68alpha |
| CG3853 | Glut3 | Glucose transporter type 3 |
| CG9042 | Gpdh | Glycerol 3 phosphate dehydrogenase |
| CG8464 | HtrA2 | HtrA2 |
| CG8579 | Jon44E | Jonah 44E |
| CG8695 | Mal-A3 | Maltase A3 |
| CG11669 | Mal-A7 | Maltase A7 |
| CG13431 | Mgat1 | UDP-GlcNAc:a-3-D-mannoside-beta-1,2-N-acetylglucosaminyltransferase I |
| CG4123 | Mipp1 | Multiple inositol polyphosphate phosphatase 1 |
| CG11661 | Nc73EF | Neural conserved at 73EF |
| CG6164 | Npc2f | Niemann-Pick type C-2f |
| CG3250 | Os-C | Os-C |
| CG6666 | SdhC | Succinate dehydrogenase C |
| CG5474 | SsRbeta | Signal sequence receptor beta |
| CG9564 | Try29F | Trypsin 29F |
| CG6649 | Ugt35b | UDP-glycosyltransferase 35b |
| CG7678 | Vha100-4 | Vacuolar H[+] ATPase subunit 100-4 |
| CG12602 | Vha100-5 | Vacuolar H[+] ATPase subunit 100-5 |
| CG1031 | alpha-Est1 | alpha-Esterase-1 |
| CG3057 | colt | congested-like trachea |
| CG7445 | fln | flightin |
| CG7754 | iotaTry | iotaTrypsin |
| CG12388 | kappaTry | kappaTry |
| CG7010 | l(1)G0334 | lethal (1) G0334 |
| CG10655 | l(2)37Bb | lethal (2) 37Bb |
| CG9762 | l(3)neo18 | lethal (3) neo18 |
| CG12350 | lambdaTry | lambdaTry |
| CG16834 | lectin-33A | lectin-33A |
| CG17280 | levy | levy |
| CG3712 | mRpL33 | mitochondrial ribosomal protein L33 |
| CG42639 | proPO-A1 | prophenol oxidase A1 |
| CG16944 | sesB | stress-sensitive B |
| CG9675 | spheroide | spheroide |
| CG9032 | sun | stunted |
| CG4979 | sxe2 | sex-specific enzyme 2 |
| CG5269 | vib | vibrator |

Age up and Heat Shock up

| Gene > Secondary Identifier | Gene > Symbol | Gene > Name |
| --- | --- | --- |
| CG3821 | Aats-asp | Aspartyl-tRNA synthetase |
| CG3481 | Adh | Alcohol dehydrogenase |
| CG3484 | Adhr | Adh-related |
| CG13941 | Arc2 | Arc2 |
| CG10621 | CG10621 |  |
| CG11009 | CG11009 |  |
| CG11030 | CG11030 |  |
| CG12224 | CG12224 |  |
| CG12896 | CG12896 |  |
| CG14245 | CG14245 |  |
| CG14246 | CG14246 |  |
| CG14906 | CG14906 |  |
| CG15043 | CG15043 |  |
| CG15784 | CG15784 |  |
| CG2065 | CG2065 |  |
| CG31638 | CG31638 |  |
| CG32103 | CG32103 |  |
| CG33056 | CG33056 |  |
| CG33229 | CG33229 |  |
| CG5290 | CG5290 |  |
| CG5869 | CG5869 |  |
| CG5966 | CG5966 |  |
| CG7033 | CG7033 |  |
| CG7130 | CG7130 |  |
| CG8620 | CG8620 |  |
| CR18228 | CR18228 |  |
| CG8439 | Cct5 | T-complex Chaperonin 5 |
| CG8977 | Cctgamma | Cctgamma |
| CG3642 | Clp | Clipper |
| CG10242 | Cyp6a23 | Cyp6a23 |
| CG10578 | DnaJ-1 | DnaJ-like-1 |
| CG10810 | Drs | Drosomycin |
| CG4181 | GstD2 | Glutathione S transferase D2 |
| CG5164 | GstE1 | Glutathione S transferase E1 |
| CG17531 | GstE7 | Glutathione S transferase E7 |
| CG17533 | GstE8 | Glutathione S transferase E8 |
| CG4460 | Hsp22 | Heat shock protein 22 |
| CG4463 | Hsp23 | Heat shock protein 23 |
| CG4183 | Hsp26 | Heat shock protein 26 |
| CG4466 | Hsp27 | Heat shock protein 27 |
| CG4456 | Hsp67Bb | Heat shock gene 67Bb |
| CG4190 | Hsp67Bc | Heat shock gene 67Bc |
| CG5436 | Hsp68 | Heat shock protein 68 |
| CG18743 | Hsp70Ab | Heat-shock-protein-70Ab |
| CG31449 | Hsp70Ba | Heat-shock-protein-70Ba |
| CG31359 | Hsp70Bb | Heat-shock-protein-70Bb |
| CG5834 | Hsp70Bbb | Hsp70Bbb |
| CG6489 | Hsp70Bc | Heat-shock-protein-70Bc |
| CG1242 | Hsp83 | Heat shock protein 83 |
| CG11143 | Inos | Inos |
| CG9080 | Listericin | Listericin |
| CG17725 | Pepck | Phosphoenolpyruvate carboxykinase |
| CG12405 | Prx2540-1 | Peroxiredoxin 2540-1 |
| CG11765 | Prx2540-2 | Peroxiredoxin 2540-2 |
| CG4484 | Slc45-1 | Slc45 ortholog 1 |
| CG7219 | Spn28D | Serpin 28D |
| CG5374 | T-cp1 | Tcp1-like |
| CG6633 | Ugt86Dd | Ugt86Dd |
| CG3705 | aay | astray |
| CG7380 | baf | barrier to autointegration factor |
| CG1044 | dos | daughter of sevenless |
| CG17820 | fit | female-specific independent of transformer |
| CG4533 | l(2)efl | lethal (2) essential for life |
| CG10360 | ref(2)P | refractory to sigma P |
| CG32130 | stv | starvin |
| CG2708 | unc-45 |  |

Age down and Heat Shock down

| Gene > Secondary Identifier | Gene > Symbol | Gene > Name |
| --- | --- | --- |
| CG8896 | 18w | 18 wheeler |
| CG1262 | Acp62F | Accessory gland peptide 62F |
| CG13095 | Bace | beta-site APP-cleaving enzyme |
| CG10026 | CG10026 |  |
| CG10345 | CG10345 |  |
| CG10467 | CG10467 |  |
| CG10472 | CG10472 |  |
| CG10477 | CG10477 |  |
| CG10570 | CG10570 |  |
| CG10592 | CG10592 |  |
| CG10731 | CG10731 |  |
| CG10827 | CG10827 |  |
| CG11911 | CG11911 |  |
| CG12990 | CG12990 |  |
| CG13833 | CG13833 |  |
| CG14120 | CG14120 |  |
| CG14661 | CG14661 |  |
| CG15254 | CG15254 |  |
| CG15255 | CG15255 |  |
| CG15534 | CG15534 |  |
| CG16904 | CG16904 |  |
| CG17752 | CG17752 |  |
| CG18180 | CG18180 |  |
| CG18302 | CG18302 |  |
| CG18493 | CG18493 |  |
| CG18585 | CG18585 |  |
| CG2070 | CG2070 |  |
| CG31086 | CG31086 |  |
| CG31148 | CG31148 |  |
| CG3290 | CG3290 |  |
| CG3734 | CG3734 |  |
| CG3739 | CG3739 |  |
| CG3823 | CG3823 |  |
| CG3940 | CG3940 |  |
| CG4053 | CG4053 |  |
| CG4734 | CG4734 |  |
| CG5107 | CG5107 |  |
| CG5150 | CG5150 |  |
| CG5804 | CG5804 |  |
| CG5945 | CG5945 |  |
| CG6295 | CG6295 |  |
| CG6660 | CG6660 |  |
| CG6839 | CG6839 |  |
| CG7542 | CG7542 |  |
| CG7916 | CG7916 |  |
| CG7953 | CG7953 |  |
| CG8093 | CG8093 |  |
| CG8147 | CG8147 |  |
| CG8708 | CG8708 |  |
| CG8834 | CG8834 |  |
| CG8997 | CG8997 |  |
| CG9463 | CG9463 |  |
| CG9466 | CG9466 |  |
| CG9468 | CG9468 |  |
| CG9497 | CG9497 |  |
| CG9673 | CG9673 |  |
| CG9682 | CG9682 |  |
| CG30042 | Cpr49Ab | Cuticular protein 49Ab |
| CG5137 | Cyp312a1 | Cyp312a1 |
| CG3360 | Cyp313a1 | Cyp313a1 |
| CG6730 | Cyp4d21 | Cyp4d21 |
| CG8433 | Ext2 | Ext2 |
| CG8579 | Jon44E | Jonah 44E |
| CG6467 | Jon65Aiv | Jonah 65Aiv |
| CG6298 | Jon74E | Jonah 74E |
| CG8695 | Mal-A3 | Maltase A3 |
| CG11669 | Mal-A7 | Maltase A7 |
| CG4123 | Mipp1 | Multiple inositol polyphosphate phosphatase 1 |
| CG11661 | Nc73EF | Neural conserved at 73EF |
| CG12813 | Npc2d | Niemann-Pick type C-2d |
| CG6164 | Npc2f | Niemann-Pick type C-2f |
| CG7592 | Obp99b | Odorant-binding protein 99b |
| CG3250 | Os-C | Os-C |
| CG14745 | PGRP-SC2 | PGRP-SC2 |
| CG4812 | Ser8 | Ser8 |
| CG12602 | Vha100-5 | Vacuolar H[+] ATPase subunit 100-5 |
| CG1031 | alpha-Est1 | alpha-Esterase-1 |
| CG18444 | alphaTry | alphaTrypsin |
| CG8084 | ana | anachronism |
| CG18211 | betaTry | betaTrypsin |
| CG7754 | iotaTry | iotaTrypsin |
| CG12388 | kappaTry | kappaTry |
| CG12350 | lambdaTry | lambdaTry |
| CG16834 | lectin-33A | lectin-33A |
| CG3712 | mRpL33 | mitochondrial ribosomal protein L33 |
| CG1803 | regucalcin | regucalcin |
| CG16944 | sesB | stress-sensitive B |
| CG9675 | spheroide | spheroide |
| CG4979 | sxe2 | sex-specific enzyme 2 |

Age up and Irradiation up

| Gene > Secondary Identifier | Gene > Symbol | Gene > Name |
| --- | --- | --- |
| CG12876 | ALiX | ALG-2 interacting protein X |
| CG3821 | Aats-asp | Aspartyl-tRNA synthetase |
| CG5178 | Act88F | Actin 88F |
| CG11654 | Ahcy13 | Adenosylhomocysteinase at 13 |
| CG5730 | AnnIX | Annexin IX |
| CG9968 | Anxb11 | Annexin B11 |
| CG13941 | Arc2 | Arc2 |
| CG10146 | AttA | Attacin-A |
| CG18372 | AttB | Attacin-B |
| CG10103 | CG10103 |  |
| CG10126 | CG10126 |  |
| CG10638 | CG10638 |  |
| CG10641 | CG10641 |  |
| CG10916 | CG10916 |  |
| CG11009 | CG11009 |  |
| CG11030 | CG11030 |  |
| CG11089 | CG11089 |  |
| CG1139 | CG1139 |  |
| CG11686 | CG11686 |  |
| CG11880 | CG11880 |  |
| CG11899 | CG11899 |  |
| CG12012 | CG12012 |  |
| CG12224 | CG12224 |  |
| CG12896 | CG12896 |  |
| CG13116 | CG13116 |  |
| CG13117 | CG13117 |  |
| CG13887 | CG13887 |  |
| CG13905 | CG13905 |  |
| CG14109 | CG14109 |  |
| CG14207 | CG14207 |  |
| CG14245 | CG14245 |  |
| CG14246 | CG14246 |  |
| CG14401 | CG14401 |  |
| CG14545 | CG14545 |  |
| CG14695 | CG14695 |  |
| CG14906 | CG14906 |  |
| CG14907 | CG14907 |  |
| CG15065 | CG15065 |  |
| CG1572 | CG1572 |  |
| CG15784 | CG15784 |  |
| CG16978 | CG16978 |  |
| CG17904 | CG17904 |  |
| CG18473 | CG18473 |  |
| CG1943 | CG1943 |  |
| CG2064 | CG2064 |  |
| CG2201 | CG2201 |  |
| CG2233 | CG2233 |  |
| CG2909 | CG2909 |  |
| CG3008 | CG3008 |  |
| CG3011 | CG3011 |  |
| CG3036 | CG3036 |  |
| CG31638 | CG31638 |  |
| CG31705 | CG31705 |  |
| CG32103 | CG32103 |  |
| CG33229 | CG33229 |  |
| CG34325 | CG34325 |  |
| CG3448 | CG3448 |  |
| CG3590 | CG3590 |  |
| CG3999 | CG3999 |  |
| CG4199 | CG4199 |  |
| CG4269 | CG4269 |  |
| CG5080 | CG5080 |  |
| CG5290 | CG5290 |  |
| CG5346 | CG5346 |  |
| CG5493 | CG5493 |  |
| CG5597 | CG5597 |  |
| CG5955 | CG5955 |  |
| CG5966 | CG5966 |  |
| CG6043 | CG6043 |  |
| CG6066 | CG6066 |  |
| CG6188 | CG6188 |  |
| CG6272 | CG6272 |  |
| CG6353 | CG6353 |  |
| CG6767 | CG6767 |  |
| CG6972 | CG6972 |  |
| CG7033 | CG7033 |  |
| CG7130 | CG7130 |  |
| CG7192 | CG7192 |  |
| CG7267 | CG7267 |  |
| CG8112 | CG8112 |  |
| CG8317 | CG8317 |  |
| CG8678 | CG8678 |  |
| CG9336 | CG9336 |  |
| CG9547 | CG9547 |  |
| CG9689 | CG9689 |  |
| CG9989 | CG9989 |  |
| CG4618 | CHMP2B | Charged multivesicular body protein 2b |
| CG8439 | Cct5 | T-complex Chaperonin 5 |
| CG8977 | Cctgamma | Cctgamma |
| CG10843 | Cyp4p3 | Cyp4p3 |
| CG5729 | Dgp-1 | Dgp-1 |
| CG10816 | Dro | Drosocin |
| CG3903 | Gli | Gliotactin |
| CG6176 | Grip75 | Grip75 |
| CG2718 | Gs1 | Glutamine synthetase 1 |
| CG4181 | GstD2 | Glutathione S transferase D2 |
| CG5164 | GstE1 | Glutathione S transferase E1 |
| CG7399 | Hn | Henna |
| CG4460 | Hsp22 | Heat shock protein 22 |
| CG4456 | Hsp67Bb | Heat shock gene 67Bb |
| CG18743 | Hsp70Ab | Heat-shock-protein-70Ab |
| CG31449 | Hsp70Ba | Heat-shock-protein-70Ba |
| CG31359 | Hsp70Bb | Heat-shock-protein-70Bb |
| CG5834 | Hsp70Bbb | Hsp70Bbb |
| CG6489 | Hsp70Bc | Heat-shock-protein-70Bc |
| CG1242 | Hsp83 | Heat shock protein 83 |
| CG4472 | Idgf1 | Imaginal disc growth factor 1 |
| CG4559 | Idgf3 | Imaginal disc growth factor 3 |
| CG10160 | ImpL3 | Ecdysone-inducible gene L3 |
| CG11143 | Inos | Inos |
| CG5247 | Irbp | Inverted repeat-binding protein |
| CG8913 | Irc | Immune-regulated catalase |
| CG10922 | La | La autoantigen-like |
| CG12369 | Lac | Lachesin |
| CG9080 | Listericin | Listericin |
| CG18362 | Mio | Mlx interactor |
| CG7438 | Myo31DF | Myosin 31DF |
| CG2330 | Neurochondrin | Neurochondrin |
| CG18466 | Nmdmc | NAD-dependent methylenetetrahydrofolate dehydrogenase |
| CG12752 | Nxt1 | NTF2-related export protein 1 |
| CG11709 | PGRP-SA | Peptidoglycan recognition protein SA |
| CG17725 | Pepck | Phosphoenolpyruvate carboxykinase |
| CG6876 | Prp31 |  |
| CG12405 | Prx2540-1 | Peroxiredoxin 2540-1 |
| CG11765 | Prx2540-2 | Peroxiredoxin 2540-2 |
| CG31864 | Qtzl | Quetzalcoatl |
| CG11992 | Rel | Relish |
| CG5371 | RnrL | Ribonucleoside diphosphate reductase large subunit |
| CG9633 | RpA-70 | Replication Protein A 70 |
| CG4173 | 2-Sep | Septin-2 |
| CG3926 | Spat | Serine pyruvate aminotransferase |
| CG11331 | Spn27A | Serpin 27A |
| CG18525 | Spn5 | Serine protease inhibitor 5 |
| CG6687 | Spn88Eb | Serpin 88Eb |
| CG4817 | Ssrp | Structure specific recognition protein |
| CG12846 | Tsp42Ed | Tetraspanin 42Ed |
| CG12844 | Tsp42Eh | Tetraspanin 42Eh |
| CG12843 | Tsp42Ei | Tetraspanin 42Ei |
| CG6120 | Tsp96F | Tetraspanin 96F |
| CG7171 | Uro | Urate oxidase |
| CG17836 | Xrp1 |  |
| CG3705 | aay | astray |
| CG9127 | ade2 | adenosine 2 |
| CG31628 | ade3 | adenosine 3 |
| CG3989 | ade5 | ade5 |
| CG12132 | c11.1 | c11.1 |
| CG5848 | cact | cactus |
| CG10460 | cer | crammer |
| CG9553 | chic | chickadee |
| CG4944 | cib | ciboulot |
| CG3365 | drongo | drongo |
| CG2086 | drpr | draper |
| CG4153 | eIF-2beta | Eukaryotic initiation factor 2beta |
| CG5202 | escl | escl |
| CG15825 | fon | fondue |
| CG9734 | glob1 | globin 1 |
| CG1487 | krz | kurtz |
| CG4533 | l(2)efl | lethal (2) essential for life |
| CG7769 | pic | piccolo |
| CG2467 | pot | papillote |
| CG7850 | puc | puckered |
| CG6339 | rad50 | rad50 |
| CG10360 | ref(2)P | refractory to sigma P |
| CG2948 | rev7 | rev7 |
| CG1697 | rho-4 | rhomboid-4 |
| CG10964 | sni | sniffer |
| CG3595 | sqh | spaghetti squash |
| CG32130 | stv | starvin |
| CG6863 | tok | tolkin |
| CG2708 | unc-45 |  |
| CG31764 | vir-1 | virus-induced RNA 1 |
| CG42365 |  |  |
| CG42807 |  |  |

Age down and Irradiation down

| Gene > Secondary Identifier | Gene > Symbol | Gene > Name |
| --- | --- | --- |
| CG8896 | 18w | 18 wheeler |
| CG12251 | AQP | aquaporin |
| CG4412 | ATPsyn-Cf6 | ATPase coupling factor 6 |
| CG10593 | Acer | Angiotensin-converting enzyme-related |
| CG9244 | Acon | Aconitase |
| CG11140 | Aldh-III | Aldehyde dehydrogenase type III |
| CG1462 | Aph-4 | Alkaline phosphatase 4 |
| CG5166 | Atx2 | Ataxin-2 |
| CG12487 | BobA | Brother of Bearded A |
| CG10026 | CG10026 |  |
| CG10345 | CG10345 |  |
| CG10467 | CG10467 |  |
| CG10570 | CG10570 |  |
| CG10734 | CG10734 |  |
| CG11023 | CG11023 |  |
| CG11112 | CG11112 |  |
| CG11165 | CG11165 |  |
| CG11378 | CG11378 |  |
| CG11752 | CG11752 |  |
| CG11912 | CG11912 |  |
| CG12493 | CG12493 |  |
| CG13091 | CG13091 |  |
| CG13101 | CG13101 |  |
| CG13155 | CG13155 |  |
| CG13465 | CG13465 |  |
| CG13607 | CG13607 |  |
| CG13898 | CG13898 |  |
| CG14072 | CG14072 |  |
| CG14120 | CG14120 |  |
| CG14141 | CG14141 |  |
| CG14277 | CG14277 |  |
| CG1444 | CG1444 |  |
| CG14482 | CG14482 |  |
| CG14509 | CG14509 |  |
| CG14589 | CG14589 |  |
| CG14629 | CG14629 |  |
| CG14661 | CG14661 |  |
| CG14676 | CG14676 |  |
| CG15434 | CG15434 |  |
| CG15534 | CG15534 |  |
| CG15617 | CG15617 |  |
| CG15841 | CG15841 |  |
| CG1648 | CG1648 |  |
| CG16904 | CG16904 |  |
| CG16964 | CG16964 |  |
| CG17029 | CG17029 |  |
| CG18063 | CG18063 |  |
| CG1809 | CG1809 |  |
| CG18302 | CG18302 |  |
| CG18327 | CG18327 |  |
| CG18493 | CG18493 |  |
| CG18585 | CG18585 |  |
| CG2070 | CG2070 |  |
| CG30427 | CG30427 |  |
| CG3088 | CG3088 |  |
| CG3106 | CG3106 |  |
| CG31086 | CG31086 |  |
| CG31148 | CG31148 |  |
| CG31391 | CG31391 |  |
| CG31802 | CG31802 |  |
| CG31883 | CG31883 |  |
| CG3290 | CG3290 |  |
| CG3301 | CG3301 |  |
| CG33259 | CG33259 |  |
| CG33521 | CG33521 |  |
| CG3560 | CG3560 |  |
| CG3610 | CG3610 |  |
| CG3734 | CG3734 |  |
| CG3823 | CG3823 |  |
| CG3927 | CG3927 |  |
| CG3940 | CG3940 |  |
| CG4000 | CG4000 |  |
| CG4021 | CG4021 |  |
| CG42235 | CG42235 |  |
| CG4847 | CG4847 |  |
| CG5023 | CG5023 |  |
| CG5037 | CG5037 |  |
| CG5050 | CG5050 |  |
| CG5107 | CG5107 |  |
| CG5150 | CG5150 |  |
| CG5618 | CG5618 |  |
| CG5755 | CG5755 |  |
| CG5804 | CG5804 |  |
| CG5903 | CG5903 |  |
| CG5991 | CG5991 |  |
| CG6293 | CG6293 |  |
| CG6555 | CG6555 |  |
| CG6660 | CG6660 |  |
| CG6726 | CG6726 |  |
| CG6733 | CG6733 |  |
| CG7069 | CG7069 |  |
| CG7409 | CG7409 |  |
| CG7430 | CG7430 |  |
| CG7580 | CG7580 |  |
| CG7716 | CG7716 |  |
| CG7768 | CG7768 |  |
| CG7804 | CG7804 |  |
| CG8093 | CG8093 |  |
| CG8097 | CG8097 |  |
| CG8147 | CG8147 |  |
| CG8329 | CG8329 |  |
| CG8560 | CG8560 |  |
| CG8562 | CG8562 |  |
| CG8773 | CG8773 |  |
| CG8834 | CG8834 |  |
| CG9463 | CG9463 |  |
| CG9466 | CG9466 |  |
| CG9468 | CG9468 |  |
| CG9682 | CG9682 |  |
| CG9921 | CG9921 |  |
| CR11700 | CR11700 |  |
| CG6022 | Cchl | Cytochrome c heme lyase |
| CG14405 | CheB38c | Chemosensory protein B 38c |
| CG2249 | CoVIIc | Cytochrome c oxidase subunit VIIc |
| CG14235 | CoVIb | Cytochrome c oxidase subunit VIb |
| CG15077 | Cyp12b2 | Cyp12b2 |
| CG8733 | Cyp305a1 | Cyp305a1 |
| CG5137 | Cyp312a1 | Cyp312a1 |
| CG3360 | Cyp313a1 | Cyp313a1 |
| CG8302 | Cyp4aa1 | Cyp4aa1 |
| CG3540 | Cyp4d14 | Cyp4d14 |
| CG2062 | Cyp4e1 | Cytochrome P450-4e1 |
| CG17903 | Cyt-c-p | Cytochrome c proximal |
| CG32072 | Elo68alpha | Elongase 68alpha |
| CG3853 | Glut3 | Glucose transporter type 3 |
| CG9042 | Gpdh | Glycerol 3 phosphate dehydrogenase |
| CG43388 | Hk | Hyperkinetic |
| CG8464 | HtrA2 | HtrA2 |
| CG8579 | Jon44E | Jonah 44E |
| CG12192 | Klp59D | Klp59D |
| CG11669 | Mal-A7 | Maltase A7 |
| CG4123 | Mipp1 | Multiple inositol polyphosphate phosphatase 1 |
| CG6004 | Muc68D | Mucin 68D |
| CG11661 | Nc73EF | Neural conserved at 73EF |
| CG6164 | Npc2f | Niemann-Pick type C-2f |
| CG4307 | Oscp | Oligomycin sensitivity-conferring protein |
| CG31794 | Pax | Paxillin |
| CG17246 | SdhA | Succinate dehydrogenase A |
| CG9214 | Tob | Tob |
| CG12408 | TpnC4 | Troponin C isoform 4 |
| CG9564 | Try29F | Trypsin 29F |
| CG6649 | Ugt35b | UDP-glycosyltransferase 35b |
| CG2512 | alphaTub84D | alpha-Tubulin at 84D |
| CG5268 | blp | black pearl |
| CG4832 | cnn | centrosomin |
| CG10192 | eIF4G2 | eukaryotic translation initiation factor 4G2 |
| CG7445 | fln | flightin |
| CG7754 | iotaTry | iotaTrypsin |
| CG12388 | kappaTry | kappaTry |
| CG7010 | l(1)G0334 | lethal (1) G0334 |
| CG6105 | l(2)06225 | lethal (2) 06225 |
| CG13240 | l(2)35Di | lethal (2) 35Di |
| CG9762 | l(3)neo18 | lethal (3) neo18 |
| CG12350 | lambdaTry | lambdaTry |
| CG16834 | lectin-33A | lectin-33A |
| CG17280 | levy | levy |
| CG3712 | mRpL33 | mitochondrial ribosomal protein L33 |
| CG43113 | orb2 |  |
| CG8764 | ox | oxen |
| CG7815 | ran-like | ran-like |
| CG16944 | sesB | stress-sensitive B |
| CG1417 | slgA | sluggish A |
| CG9032 | sun | stunted |
| CG4979 | sxe2 | sex-specific enzyme 2 |
| CG5269 | vib | vibrator |
| CG34438 | Cap-G |  |
| CG34434 | CG34434 |  |
