# Supplementary material for: Gene expression changes in response to aging compared to heat stress, oxidative stress and ionizing radiation in Drosophila melanogaster
Source: Aging (Albany NY). 2012 Nov 30;4(11):768–89. doi: 10.18632/aging.100499 (PMC3560439; doi:10.18632/aging.100499)
Supplement: Supplementary file 4 [file aging-04-768-s004.docx]

Supplemental Table S3. Features common to aging and individual stresses (sugar excluded)

a) GO enrichment terms for genes up-regulated in aging and in hyperoxia

| GO:0009408 | response to heat(16) | 2.64E-11 |
| --- | --- | --- |
| GO:0009266 | response to temperature stimulus(17) | 1.15E-10 |
| GO:0006950 | response to stress(34) | 9.95E-08 |
| GO:0035079 | polytene chromosome puffing(5) | 3.88E-05 |
| GO:0035080 | heat shock-mediated polytene chromosome( puffing(5) | 3.88E-05 |
| GO:0009628 | response to abiotic stimulus(17) | 8.38E-05 |
| GO:0044271 | cellular nitrogen compound biosynthetic process(14) | 0.00202 |
| GO:0034605 | cellular response to heat(6) | 0.002657 |
| GO:0019731 | antibacterial humoral response(7) | 0.009046 |
| GO:0009156 | ribonucleoside monophosphate biosynthetic process(5) | 0.032356 |
| GO:0009161 | ribonucleoside monophosphate metabolic process(5) | 0.032356 |
| GO:0006564 | L-serine biosynthetic process(3) | 0.04487 |

b) GO enrichment terms for genes that down-regulated in aging and in hyperoxia

| GO:0006508 | Proteolysis(56) | 6.01E-18 |
| --- | --- | --- |
| GO:0045297 | post-mating behavior(7) | 9.33E-04 |
| GO:0008152 | metabolic process(131) | 0.00614 |

c) GO enrichment terms for genes up-regulated in aging and hydrogen peroxide

| GO:0009408 | response to heat(11) | 4.75E-06 |
| --- | --- | --- |
| GO:0035079 | polytene chromosome puffing(5) | 1.44E-05 |
| GO:0035080 | heat shock-mediated polytene chromosome puffing(6) | 1.44E-05 |
| GO:0006950 | response to stress(27) | 2.86E-05 |
| GO:0009266 | response to temperature stimulus(11) | 7.87E-05 |
| GO:0034605 | cellular response to heat(6) | 8.32E-04 |
| GO:0044271 | cellular nitrogen compound biosynthetic process(13) | 0.001277 |
| GO:0009069 | serine family amino acid metabolic process(5) | 0.005345 |
| GO:0044281 | small molecule metabolic process(24) | 0.005367 |
| GO:0006564 | L-serine biosynthetic process(3) | 0.024923 |
| GO:0051707 | response to other organism(11) | 0.032029 |
| GO:0009607 | response to biotic stimulus(11) | 0.035404 |

d) GO enrichment terms for genes down-regulated in aging and hydrogen peroxide

| GO:0006091 | generation of precursor metabolites and energy(12) | 0.001493 |
| --- | --- | --- |
| GO:0022900 | electron transport chain(9) | 0.001518 |
| GO:0055114 | oxidation-reduction process(22) | 0.002986 |
| GO:0045333 | cellular respiration(10) | 0.003694 |
| GO:0015980 | energy derivation by oxidation of organic compounds(10) | 0.008153 |
| GO:0022904 | respiratory electron transport chain(8) | 0.010626 |
| GO:0042775 | mitochondrial ATP synthesis coupled electron transport(7) | 0.043253 |

e) GO enrichment terms for genes up-regulated in aging and heat stress

| GO:0009408 | response to heat(18) | 1.04E-21 |
| --- | --- | --- |
| GO:0009266 | response to temperature stimulus(18) | 1.68E-19 |
| GO:0009628 | response to abiotic stimulus(18) | 8.84E-13 |
| GO:0006950 | response to stress(22) | 1.70E-08 |
| GO:0006457 | protein folding(11) | 1.90E-07 |
| GO:0035079 | polytene chromosome puffing(5) | 4.29E-07 |
| GO:0035080 | heat shock-mediated polytene chromosome puffing(5) | 4.29E-07 |
| GO:0034605 | cellular response to heat(6) | 1.27E-05 |
| GO:0001666 | response to hypoxia(6) | 0.001193 |
| GO:0070482 | response to oxygen levels(6) | 0.002157 |
| GO:0042221 | response to chemical stimulus(14) | 0.031137 |

f) GO enrichment terms for genes down-regulated in aging and heat stress

| GO:0006508 | Proteolysis(25) | 1.75E-07 |
| --- | --- | --- |

g) GO enrichment terms for genes up-regulated in aging and Ionizing radiation

| GO:0006950 | response to stress(33) | | | | 2.17E-06 |  |
| --- | --- | --- | --- | --- | --- | --- |
| GO:0009408 | response to heat(12) | | | | 5.07E-06 |  |
| GO:0035079 | polytene chromosome puffing(5) | | | | 5.04E-05 |  |
| GO:0035080 | heat shock-mediated polytene chromosome puffing(5) | | | | 5.04E-05 |  |
| GO:0009266 | response to temperature stimulus(12) | | | | 1.05E-04 |  |
| GO:0034605 | cellular response to heat(6) | | | | 0.003611 |  |
| GO:0009069 | serine family amino acid metabolic process(5) | | | | 0.018171 |  |
| GO:0044271 | cellular nitrogen compound biosynthetic process(13) | | | | 0.022327 |  |
| GO:0033554 | cellular response to stress(18) | | | | 0.024714 |  |
|  | |  |  | | | |
|  | |  | |  | | |

h) GO enrichment terms for genes down-regulated in aging and Ionizing radiation

| GO:0006091 | generation of precursor metabolites and energy(17) | 2.30E-07 |
| --- | --- | --- |
| GO:0045333 | cellular respiration(14) | 1.95E-06 |
| GO:0015980 | energy derivation by oxidation of organic compounds(14) | 6.16E-06 |
| GO:0022900 | electron transport chain(11) | 4.47E-05 |
| GO:0055114 | oxidation-reduction process(26) | 2.65E-04 |
| GO:0006119 | oxidative phosphorylation(10) | 2.91E-04 |
| GO:0022904 | respiratory electron transport chain(10) | 2.91E-04 |
| GO:0042775 | mitochondrial ATP synthesis coupled electron transport(9) | 9.52E-04 |
| GO:0042773 | ATP synthesis coupled electron transport(9) | 0.00164 |
