# Supplementary material for: Gene expression changes in response to aging compared to heat stress, oxidative stress and ionizing radiation in Drosophila melanogaster
Source: Aging (Albany NY). 2012 Nov 30;4(11):768–89. doi: 10.18632/aging.100499 (PMC3560439; doi:10.18632/aging.100499)
Supplement: Supplementary file 17 [file aging-04-768-s017.docx]

| **Supplemental Table S5.** | | | | |
| --- | --- | --- | --- | --- |
| **Duplicate genes** | | | | |
| **Identifier New genes Affy probe ID Lists gene appears in** | | | | |
| CG15675 | CG42365 | 153101_at | age up, H2O2 up, IR up, O2 up |  |
|  | CG42362 |  |  |  |
|  | CG42363 |  |  |  |
|  | CG42364 |  |  |  |
| Resolution: BLAST results using affy probe data: probe hits CG42365 | | | |  |
| CG13335 | CG42807 | 152245_at | age up, IR up, O2 up |  |
|  | CG42808 |  |  |  |
| Resolution: BLAST results using affy probe data: probe hits CG42807 | | | |  |
| CG8772 | CG42708 | 152880_at | H2O2 up, O2 up |  |
|  | CG8776 (nemy) |  |  |  |
| Resolution: BLAST results using affy probe data: probe hits CG42708 | | | |  |
| CG33936 | CG43675 | 149551 | H2O2 up, IR up, age down |  |
|  | CG43674 |  |  |  |
| Resolution: BLAST results using affy probe data: probe hits CG43675 | | | |  |
| CG12816 | CG34107 | 149622_at | HS up, age down |  |
|  | CG42795 |  |  |  |
| Resolution: BLAST results using affy probe data: probe hits CG34107 | | | |  |
| CG13353 | CG42288 | 147185_at | HS up, age down |  |
|  | CG42287 |  |  |  |
| Resolution: BLAST results using affy probe data: probe hits CG42288 | | | |  |
| CG6784 | CG42827 | 150441_at | O2 up, age down |  |
|  | CG42828 |  |  |  |
| Resolution: BLAST results using affy probe data: probe hits CG42827 | | | |  |
| CG17054 | CG34438 (Cap-G) | 147139_at | age down, IR down |  |
|  | CG34439 |  |  |  |
| Resolution: BLAST results using affy probe data: probe hits CG34438 (Cap-G) | | | |  |
| CG17763 | CG34434 | 147768_at | age down, IR down |  |
|  | CG34435 |  |  |  |
| Resolution: BLAST results using affy probe data: probe hits CG34434 | | | |  |
| CG11079 | CG34424 | 147768_at | H2O2 down, IR down, HS down |  |
|  | CG34423 |  |  |  |
| Resolution: BLAST results using affy probe data: probe hits CG34424 | | | |  |
| CG9511 | CG42369 | 145897_at | H2O2 down, IR down, HS down |  |
|  | CG42368 |  |  |  |
| Resolution: BLAST results using affy probe data: probe hits CG42369 | | | |  |
| CG30492 | CG43340 | 145897_at | H2O2 down, O2 down |  |
|  | CG43341 |  |  |  |
| Resolution: BLAST results using affy probe data: probe hits CG43440 | | | |  |
| CG32158 | CG43373 | 141806_at | age up, HS down |  |
|  | CG42514 |  |  |  |
| Unresolved-affy probe ambiguously mapped to two Entrez Gene identifiers | | | |  |
| CG9216 | CG42353 | 151936_at | age up |  |
|  | CG42354 |  |  |  |
| Unresolved-affy probe ambiguously mapped to two Entrez Gene identifiers | | | |  |
| CG12240 | CG42567 (DnaJ-60) | 142946_at | H2O2 up, age down |  |
|  | CG42568 |  |  |  |
| Unresolved-affy probe ambiguously mapped to two Entrez Gene identifiers | | | |  |
| CG31722 | CG17140 | 146199_at | H2O2 up |  |
|  | CG17139 |  |  |  |
| Unresolved-affy probe ambiguously mapped to two Entrez Gene identifiers | | | |  |
| CG13981 | CG42368 | 145895_at | H2O2 up |  |
|  | CG42370 |  |  |  |
|  | CG42369 |  |  |  |
| Unresolved-affy probe ambiguously mapped to three Entrez Gene identifiers | | | |  |
| CG5134 | CG42518 | 153608_at | H2O2 up |  |
|  | CG42517 (MED9) |  |  |  |
| Unresolved-affy probe ambiguously mapped to two Entrez Gene identifiers | | | |  |
| CG12455 | CG42818 | 144270_at | H2O2 up |  |
|  | CG42817 |  |  |  |
| Resolution: BLAST results using affy probe data: probe hits CG42818 | | | |  |
| CG7319 | CG42631 (mtTFB1) | 154901_at | IR up |  |
|  | CG42630 |  |  |  |
| Unresolved-affy probe ambiguously mapped to two Entrez Gene identifiers | | | |  |
| CG31169 | CG43343 | 153590_at | HS up |  |
|  | CG43342 |  |  |  |
| Resolution: BLAST results using affy probe data: probe hits CG43343 | | | |  |
| CG33936 | CG43674 | 149551_at | HS up |  |
|  | CG43675 |  |  |  |
| Unresolved-affy probe ambiguously mapped to two Entrez Gene identifiers | | | |  |
| CG12677 | CG34177 | 151183_at | HS up |  |
|  | CG34178 |  |  |  |
| Unresolved-affy probe ambiguously mapped to two Entrez Gene identifiers | | | |  |
| CG13037 | CG42648 (mRpS34) | 148886_at |  |  |
|  | CG33257 |  |  |  |
| Resolution: BLAST results using affy probe data: probe hits CG42648 (mRpS34) | | | |  |
| CG8742 | CG42636 (Gyc76C) | 143732_at | HS up |  |
|  | CG42637 |  |  |  |
| Unresolved-affy probe ambiguously mapped to two Entrez Gene identifiers | | | |  |
| CG6069 | CG34130 | 150653_at | HS up |  |
|  | CG34129 |  |  |  |
| Resolution: BLAST results using affy probe data: probe hits CG34130 | | | |  |
| CG6640 | CG42825 | 152757_at | HS up |  |
|  | CG42826 |  |  |  |
| Resolution: BLAST results using affy probe data: probe hits CG42825 | | | |  |
|  | | | |  |
| **Unresolved identifiers** | | | |  |
| Unresolved identifiers : H2O2 up | | | |  |
| CG13235 | | | |  |
| CG2042 | | | | |
| CG12587 | | | | |
| CG15292 | | | | |
| CG11260 | | | | |
| CG17967 | | | | |
| CG14809 | | | | |
| CG14368 | | | | |
| CG32636 | | | | |
| CG14773 | | | | |
| CG9094 | | | | |
| CG15494 | | | | |
| CG12378 | | | | |
| CG13291 | | | | |
| CG14494 | | | | |
| Unresolved identifiers : IR up | | | | |
| CG15911 | | | | |
| Unresolved identifiers : O2 up | | | | |
| CG15057 | | | | |
| Unresolved identifiers : H2O2 up, HS up | | | | |
| CG12454 | | | | |
| Unresolved identifiers : H2O2 down | | | | |
| CG17745 | | | | |
| CG12667 | | | | |
| Unresolved identifiers : H2O2 down, IR down | | | | |
| CG12650 | | | | |
| Unresolved identifiers : H2O2 down, IR down | | | | |
| CG13828 | | | | |
| Unresolved identifiers : age down, H2O2 down, O2 down | | | | |
| CG12656 | | | | |
| Unresolved identifiers : H2O2 up, sugar up, H2O2 down, IR down, HS do | | | | |
| CG13542 | | | | |
| Unresolved identifiers : sugar down | | | | |
| CG14460 | | | | |
| Unresolved identifiers : age down, H2O2 down, IR down, O2 down, sugar down | | | | |
| CG33173 | | | | |
| Unresolved identifiers : sugar up, IR down, HS down | | | | |
| CG16937 | | | | |
|  | | | | |
|  | | | | |
