# Supplementary material for: Gene expression changes in response to aging compared to heat stress, oxidative stress and ionizing radiation in Drosophila melanogaster
Source: Aging (Albany NY). 2012 Nov 30;4(11):768–89. doi: 10.18632/aging.100499 (PMC3560439; doi:10.18632/aging.100499)
Supplement: Supplementary file 18 [file aging-04-768-s018.pdf]

Supplemental Table S6. Sugar up-regulated genes

Gene > Secondary Identifier	Gene > Symbol	Gene > Name	Gene > DB identifier
CG10033	for	foraging	FBgn0000721
CG1007	emc	extra macrochaetae	FBgn0000575
CG10128	tra2	transformer 2	FBgn0003742
CG10207	NaPi-T	Na[+]-dependent inorganic phosphate cotransporter	FBgn0016684
CG10241	Cyp6a17	Cyp6a17	FBgn0015714
CG10289	CG10289		FBgn0035688
CG10433	CG10433		FBgn0034638
CG1059	Karybeta3	Karyopherin beta 3	FBgn0087013
CG10645	lama	lamina ancestor	FBgn0016031
CG10701	Moe	Moesin	FBgn0011661
CG10706	SK	small conductance calcium-activated potassium channel	FBgn0029761
CG10810	Drs	Drosomycin	FBgn0010381
CG10812	dro5	drosomycin-5	FBgn0035434
CG10824	CG10824		FBgn0038865
CG10833	Cyp28d1	Cyp28d1	FBgn0031689
CG1088	Vha26	Vacuolar H[+]-ATPase 26kD E subunit	FBgn0015324
CG10912	CG10912		FBgn0034296
CG11154	ATPsyn-beta	ATP synthase-beta	FBgn0010217
CG11165	CG11165		FBgn0033238
CG11198	ACC	Acetyl-CoA carboxylase	FBgn0033246
CG11259	MICAL-like	MICAL-like	FBgn0036333
CG11279	CG11279		FBgn0036342
CG11301	Mes4	Mes4	FBgn0034726
CG11400	CG11400		FBgn0034198
CG11407	CG11407		FBgn0038733
CG11589	VhaM9.7-c	Vacuolar H[+] ATPase subunit M9.7-c	FBgn0028664
CG11661	Nc73EF	Neural conserved at 73EF	FBgn0010352
CG11711	Mob2	Mob2	FBgn0259481
CG11791	CG11791		FBgn0039266
CG11796	CG11796		FBgn0036992
CG11842	CG11842		FBgn0039629

CG11893	CG11893		FBgn0039316
CG12008	kst	karst	FBgn0004167
CG1210	Pdk1	Phosphoinositide -dependent kinase 1	FBgn0020386
CG12116	CG12116		FBgn0030041
CG12163	CG12163		FBgn0260462
CG12288	CG12288		FBgn0032620
CG12338	CG12338		FBgn0033543
CG12737	Crag	Calmodulin-binding protein related to a Rab3 GDP/GTP exchange protein	FBgn0025864
CG12775	RpL21	Ribosomal protein L21	FBgn0032987
CG12780	CG12780		FBgn0033301
CG12868	CG12868		FBgn0033945
CG12895	CG12895		FBgn0033523
CG13215	CG13215		FBgn0033592
CG13272	CG13272		FBgn0086673
CG13315	CG13315		FBgn0040827
CG13849	Nop56	Nop56	FBgn0038964
CG13868	CG13868		FBgn0034501
CG14029	vri	vrille	FBgn0016076
CG1412	RhoGAP19D	RhoGAP19D	FBgn0031118
CG14125	CG14125		FBgn0036232
CG14176	Or67b	Odorant receptor 67b	FBgn0036019
CG14400	CG14400		FBgn0032896
CG14535	CG14535		FBgn0031955
CG14648	lost	lost	FBgn0263594
CG14687	CG14687		FBgn0037835
CG14782	CG14782		FBgn0025381
CG14933	CG14933		FBgn0040968
CG14935	Mal-B2	Maltase B2	FBgn0032382
CG14949	CG14949		FBgn0035358
CG15019	CG15019		FBgn0035541
CG15040	CG15040		FBgn0030940
CG15067	CG15067		FBgn0034331
CG15432	CG15432		FBgn0031603
CG15745	CG15745		FBgn0030469
CG15870	CG15870		FBgn0033798
CG1600	CG1600		FBgn0033188
CG1607	CG1607		FBgn0039844
CG16707	vsg	visgun	FBgn0045823
CG16836	CG16836		FBgn0040735
CG16844	IM3	Immune induced molecule 3	FBgn0040736

CG16986	CG16986		FBgn0035356
CG17210	scpr-B	SCP-containing protein B	FBgn0037888
CG17369	Vha55	Vacuolar H[+]-ATPase 55kD B subunit	FBgn0005671
CG17599	CG17599		FBgn0031196
CG1793	MED26	Mediator complex subunit 26	FBgn0039923
CG18106	IM2	Immune induced molecule 2	FBgn0025583
CG18107	CG18107		FBgn0034330
CG18108	IM1	Immune induced molecule 1	FBgn0034329
CG18135	CG18135		FBgn0036837
CG18193	CG18193		FBgn0037579
CG1821	RpL31	Ribosomal protein L31	FBgn0025286
CG18321	miple2	miple2	FBgn0029002
CG18609	CG18609		FBgn0034382
CG18619	CG18619		FBgn0032202
CG1984	djl	don juan like	FBgn0037463
CG2050	mod	modulo	FBgn0002780
CG2173	Rs1	Rs1	FBgn0021995
CG2196	salt	salty dog	FBgn0039872
CG2216	Fer1HCH	Ferritin 1 heavy chain homologue	FBgn0015222
CG2248	Rac1	Rac1	FBgn0010333
CG2249	CG2249		FBgn0040773
CG2257	Ubc-E2H	Ubc-E2H	FBgn0029996
CG2471	Sclp	Sclp	FBgn0030357
CG2671	l(2)gl	lethal (2) giant larvae	FBgn0002121
CG2699	Pi3K21B	Pi3K21B	FBgn0020622
CG2790	CG2790		FBgn0027599
CG2947	HIP-R	Hsc/Hsp70-interacting protein related	FBgn0029676
CG2998	RpS28b	Ribosomal protein S28b	FBgn0030136
CG30015	CG30015		FBgn0050015
CG30456	CG30456		FBgn0050456
CG31098	CG31098		FBgn0051098
CG31151	wge	winged eye	FBgn0051151
CG31212	Ino80	Ino80	FBgn0086613
CG3164	CG3164		FBgn0025683
CG3174	Fmo-2	Flavin-containing monooxygenase 2	FBgn0033079
CG31778	CG31778		FBgn0051778
CG3186	eIF-5A	eIF-5A	FBgn0034967
CG3201	Mlc-c	Myosin light chain cytoplasmic	FBgn0004687
CG32043	CG32043		FBgn0052043

CG3226	CG3226		FBgn0029882
CG32428	CG32428		FBgn0052428
CG32464	l(3)82Fd	l(3)82Fd	FBgn0013576
CG32479	CG32479		FBgn0052479
CG32549	CG32549		FBgn0052549
CG32593	Flo-2	flotillin 2	FBgn0024753
CG32649	CG32649		FBgn0052649
CG32656	Muc11A	Mucin 11A	FBgn0052656
CG32758	CG32758		FBgn0052758
CG32789	HIP	Hsc/Hsp70-interacting protein	FBgn0260484
CG32813	CG32813		FBgn0052813
CG3320	Rab1	Rab-protein 1	FBgn0016700
CG33255	CG33255		FBgn0053255
CG33278	CG33278		FBgn0053278
CG3350	bigmax	bigmax	FBgn0039509
CG3354	Mst77F	Male-specific transcript 77F	FBgn0086915
CG3376	CG3376		FBgn0034997
CG3400	Pfrx	6-phosphofructo-2-kinase	FBgn0027621
CG3413	wdp	windpipe	FBgn0034718
CG3497	Su(H)	Suppressor of Hairless	FBgn0004837
CG3644	bic	bicaudal	FBgn0000181
CG3662	CG3662		FBgn0031285
CG3712	mRpL33	mitochondrial ribosomal protein L33	FBgn0040907
CG3823	CG3823		FBgn0029863
CG3920	l(2)k16918	lethal (2) k16918	FBgn0021800
CG3983	ns1	nucleostemin 1	FBgn0038473
CG3997	RpL39	Ribosomal protein L39	FBgn0023170
CG4067	pug	pugilist	FBgn0020385
CG4111	RpL35	Ribosomal protein L35	FBgn0029785
CG42321	CG42321		FBgn0259221
CG4233	Got2	Glutamate oxaloacetate transaminase 2	FBgn0001125
CG42331	CG42331		FBgn0259233
CG42390	CG42390		FBgn0259736
CG42543	mp	multiplexin	FBgn0260660
CG42563	inaF-D	inaF-D	FBgn0260812
CG4264	Hsc70-4	Heat shock protein cognate 4	FBgn0001219
CG4312	MtnB	Metallothionein B	FBgn0002869
CG43427	CG43427		FBgn0263346
CG4364	CG4364		FBgn0032138

CG4399	east	enhanced adult sensory threshold	FBgn0261954
CG4427	cbt	cabut	FBgn0043364
CG4491	noc	no ocelli	FBgn0005771
CG4501	bgm	bubblegum	FBgn0027348
CG4580	CG4580		FBgn0032585
CG4710	Pino	Pinocchio	FBgn0016926
CG4716	CG4716		FBgn0033820
CG4783	CG4783		FBgn0038756
CG4994	Mpcp	Mitochondrial phosphate carrier protein	FBgn0026409
CG5193	TfIIB	Transcription factor IIB	FBgn0004915
CG5203	CHIP	CHIP	FBgn0027052
CG5271	RpS27A	Ribosomal protein S27A	FBgn0003942
CG5277	Ip259	Intronic Protein 259	FBgn0025366
CG5295	bmm	brummer	FBgn0036449
CG5330	Nap1	Nucleosome assembly protein 1	FBgn0015268
CG5373	Pi3K59F	Phosphotidylinositol 3 kinase 59F	FBgn0015277
CG5395	nmd	no mitochondrial derivative	FBgn0005322
CG5411	Pde8	Phosphodiesterase 8	FBgn0034886
CG5508	CG5508		FBgn0027579
CG5527	CG5527		FBgn0039564
CG5535	CG5535		FBgn0036764
CG5547	Pect	Phosphoethanolamine cytidylyltransferase	FBgn0032482
CG5560	dob	doppelganger von brummer	FBgn0030607
CG5654	yps	ypsilon schachtel	FBgn0022959
CG5794	CG5794		FBgn0039214
CG5820	Gp150	Gp150	FBgn0013272
CG5874	Nelf-A	Negative elongation factor A	FBgn0038872
CG5887	desat1	desat1	FBgn0086687
CG5915	Rab7	Rab-protein 7	FBgn0015795
CG6004	Muc68D	Mucin 68D	FBgn0036203
CG6115	CG6115		FBgn0040985
CG6169	Dcp2	Decapping protein 2	FBgn0036534
CG6213	Vha13	Vacuolar H[+] ATPase G-subunit	FBgn0026753
CG6311	Edc3	Enhancer of decapping 3	FBgn0036735
CG6357	CG6357		FBgn0033875
CG6438	amon	amontillado	FBgn0023179

CG6455	CG6455		FBgn0019960
CG6500	Bx	Beadex	FBgn0000242
CG6501	ns2	nucleostemin 2	FBgn0034243
CG6597	CG6597		FBgn0036967
CG6721	Gap1	GTPase-activating protein 1	FBgn0004390
CG6764	RpL24-like	Ribosomal protein L24-like	FBgn0037899
CG6843	CG6843		FBgn0036827
CG6904	CG6904		FBgn0038293
CG6910	CG6910		FBgn0036262
CG6957	Oscillin	Oscillin	FBgn0031717
CG6980	CG6980		FBgn0039228
CG6998	ctp	cut up	FBgn0011760
CG7007	VhaPPA1-1	Vacuolar H[+] ATPase subunit PPA1-1	FBgn0028662
CG7069	CG7069		FBgn0038952
CG7106	lectin-28C	lectin-28C	FBgn0040099
CG7224	CG7224		FBgn0031971
CG7338	CG7338		FBgn0037073
CG7380	baf	barrier to autointegration factor	FBgn0031977
CG7398	Trn	Transportin	FBgn0024921
CG7425	eff	effete	FBgn0011217
CG7452	Syx17	Syntaxin 17	FBgn0035540
CG7535	GluClalpha	GluClalpha	FBgn0024963
CG7574	bip1	bip1	FBgn0026263
CG7586	Mcr	Macroglobulin complement-related	FBgn0020240
CG7664	crp	cropped	FBgn0001994
CG7695	CG7695		FBgn0038631
CG7763	CG7763		FBgn0040503
CG7808	RpS8	Ribosomal protein S8	FBgn0039713
CG7917	Nlp	Nucleoplasmin	FBgn0016685
CG7962	CdsA	CDP diglyceride synthetase	FBgn0010350
CG8029	VhaAC45	Vacuolar H[+] ATPase accessory protein AC45	FBgn0262515
CG8053	eIF-1A	Eukaryotic initiation factor 1A	FBgn0026250
CG8094	Hex-C	Hexokinase C	FBgn0001187
CG8147	CG8147		FBgn0043791
CG8153	mus210	mutagen-sensitive 210	FBgn0004698
CG8156	Arf51F	ADP ribosylation factor 51F	FBgn0013750
CG8174	SRPK	SRPK	FBgn0026370

CG8229	CG8229		FBgn0033356
CG8280	Ef1alpha48D	Elongation factor 1alpha48D	FBgn0000556
CG8344	RpIII128	RNA polymerase III 128kD subunit	FBgn0004463
CG8345	Cyp6w1	Cyp6w1	FBgn0033065
CG8353	CG8353		FBgn0032002
CG8358	CG8358		FBgn0037727
CG8385	Arf79F	ADP ribosylation factor 79F	FBgn0010348
CG8404	Sox15	Sox box protein 15	FBgn0005613
CG8416	Rho1	Rho1	FBgn0014020
CG8430	Got1	Glutamate oxaloacetate transaminase 1	FBgn0001124
CG8444	VhaM8.9		FBgn0037671
CG8448	mrj	mrj	FBgn0034091
CG8468	CG8468		FBgn0033913
CG8542	Hsc70-5	Heat shock protein cognate 5	FBgn0001220
CG8588	pst	pastrel	FBgn0035770
CG8651	trx	trithorax	FBgn0003862
CG8677	CG8677		FBgn0026577
CG8759	Nacalpha	Nascent polypeptide associated complex protein alpha subunit	FBgn0086904
CG8764	ox	oxen	FBgn0011227
CG8801	CG8801		FBgn0028473
CG8874	Fps85D	Fps oncogene analog	FBgn0000723
CG8965	CG8965		FBgn0031745
CG9091	RpL37a	Ribosomal protein L37a	FBgn0030616
CG9171	CG9171		FBgn0031738
CG9214	Tob	Tob	FBgn0028397
CG9261	nrv2	nervana 2	FBgn0015777
CG9277	betaTub56D	beta-Tubulin at 56D	FBgn0003887
CG9324	Pomp	Pomp	FBgn0032884
CG9434	Fst	Frost	FBgn0037724
CG9472	brv1	brivido-1	FBgn0036874
CG9621	Adgf-D	Adenosine deaminase-related growth factor D	FBgn0038172
CG9648	Max	Max	FBgn0017578
CG9655	nes	nessy	FBgn0026630
CG9696	dom	domino	FBgn0020306
CG9837	CG9837		FBgn0037635
CG9894	CG9894		FBgn0031453

CG9928	CG9928		FBgn0032472
CR4575	CR4575		
CG42747	CG42747		

Sugar down-regulated genes

Gene > Secondary Identifier	Gene > Symbol	Gene > Name	Gene > DB identifier
CG10037	vvl	ventral veins lacking	FBgn0086680
CG10096	CG10096		FBgn0038032
CG10097	CG10097		FBgn0038033
CG10120	Men	Malic enzyme	FBgn0002719
CG10125	zpg	zero population growth	FBgn0024177
CG10160	ImpL3	Ecdysone-inducible gene L3	FBgn0001258
CG1019	Mlp84B	Muscle LIM protein at 84B	FBgn0014863
CG10221	Hrd3		FBgn0028475
CG10237	CG10237		FBgn0032783
CG10374	Lsd-1	Lipid storage droplet-1	FBgn0039114
CG10472	CG10472		FBgn0035670
CG10475	Jon65Ai	Jonah 65Ai	FBgn0035667
CG10513	CG10513		FBgn0039311
CG10514	CG10514		FBgn0039312
CG10516	CG10516		FBgn0036549
CG10578	DnaJ-1	DnaJ-like-1	FBgn0263106
CG10657	CG10657		FBgn0036289
CG10706	SK	small conductance calcium-activated potassium channel	FBgn0029761
CG10747	CG10747		FBgn0032845
CG10840	eIF5B	eIF5B	FBgn0026259
CG10852	Acp63F	Accessory gland peptide 63F	FBgn0015585
CG10866	CG10866		FBgn0035475
CG10960	CG10960		FBgn0036316
CG11037	CG11037		FBgn0037038
CG11051	Nplp2	Neuropeptide-like precursor 2	FBgn0040813
CG11064	Rfabg	Retinoid- and fatty acid-binding glycoprotein	FBgn0087002
CG11143	Inos	Inos	FBgn0025885
CG11166	Eaf	ELL-associated factor	FBgn0033166
CG11200	CG11200	Carbonyl reductase	FBgn0034500

CG11379	CG11379		FBgn0040362
CG11505	CG11505		FBgn0035424
CG1152	Gld	Glucose dehydrogenase	FBgn0001112
CG11598	CG11598		FBgn0038067
CG11601	CG11601		FBgn0031244
CG11624	Ubi-p63E	Ubiquitin-63E	FBgn0003943
CG11635	CG11635		FBgn0033283
CG1165	LysS	Lysozyme S	FBgn0004430
CG11719	Mst98Ca	Male-specific RNA 98Ca	FBgn0002865
CG1179	LysB	Lysozyme B	FBgn0004425
CG1180	LysE	Lysozyme E	FBgn0004428
CG11892	CG11892		FBgn0039313
CG11911	CG11911		FBgn0031249
CG11942	skpE	skpE	FBgn0031074
CG12057	CG12057		FBgn0030098
CG12120	t	tan	FBgn0086367
CG12262	CG12262		FBgn0035811
CG12287	pdm2	POU domain protein 2	FBgn0004394
CG1231	CG1231		FBgn0035134
CG12351	deltaTry	deltaTrypsin	FBgn0010358
CG12374	CG12374		FBgn0033774
CG12385	thetaTry	thetaTrypsin	FBgn0011555
CG1242	Hsp83	Heat shock protein 83	FBgn0001233
CG12498	CG12498		FBgn0040356
CG12534	Alr	Augmenter of liver regeneration	FBgn0031068
CG1262	Acp62F	Accessory gland peptide 62F	FBgn0020509
CG12679	CG12679		FBgn0031103
CG12703	CG12703		FBgn0031069
CG12730	CG12730		FBgn0029771
CG12813	Npc2d	Niemann-Pick type C-2d	FBgn0037782
CG1287	CG1287		FBgn0037506
CG1288	CG1288		FBgn0250845
CG12902	CG12902		FBgn0033512
CG12990	CG12990		FBgn0030859
CG13061	Nplp3	Neuropeptide-like precursor 3	FBgn0042201
CG13095	Bace	beta-site APP-cleaving enzyme	FBgn0032049
CG1316	CG1316		FBgn0035526
CG1327	Ccp84Ac	Ccp84Ac	FBgn0004781
CG1330	Ccp84Ae	Ccp84Ae	FBgn0004779
CG1331	Ccp84Af	Ccp84Af	FBgn0004778
CG13310	CG13310		FBgn0035928

CG13801	CG13801		FBgn0035332
CG13873	Obp56g	Odorant-binding protein 56g	FBgn0034474
CG13907	CG13907		FBgn0035173
CG1394	CG1394		FBgn0030277
CG13977	Cyp6a18	Cyp6a18	FBgn0039519
CG13989	CG13989		FBgn0031786
CG14011	CG14011		FBgn0031722
CG14028	cype	cyclope	FBgn0015031
CG14034	CG14034		FBgn0250847
CG14080	Mkp3	Mitogen-activated protein kinase phosphatase 3	FBgn0036844
CG14191	CG14191		FBgn0030981
CG14220	CG14220		FBgn0031036
CG14224	Ubqn	Ubiquilin	FBgn0031057
CG14245	CG14245		FBgn0039452
CG14246	CG14246		FBgn0040608
CG14302	CG14302		FBgn0038647
CG14375	CCHa2	CCHamide-2	FBgn0038147
CG14471	CG14471		FBgn0033049
CG14513	yemalpha	yemanuclein alpha	FBgn0005596
CG14515	CG14515		FBgn0039648
CG14560	msopa	male-specific opa containing gene	FBgn0004414
CG14688	CG14688		FBgn0037819
CG14745	PGRP-SC2	PGRP-SC2	FBgn0043575
CG14746	PGRP-SC1a	PGRP-SC1a	FBgn0043576
CG14770	CG14770		FBgn0029573
CG14879	CG14879		FBgn0038419
CG14938	crol	crooked legs	FBgn0020309
CG14995	CG14995		FBgn0035497
CG15008	Cpr64Ac	Cuticular protein 64Ac	FBgn0035512
CG15147	CG15147		FBgn0032654
CG15155	CG15155		FBgn0032669
CG15199	CG15199		FBgn0030270
CG15239	CG15239		FBgn0029681
CG15278	CG15278		FBgn0032554
CG15353	CG15353		FBgn0040718
CG15358	CG15358		FBgn0031373
CG15572	CG15572		FBgn0029702
CG15616	Acp53C14b	Acp53C14b	FBgn0034153
CG15635	CG15635		FBgn0031617
CG15704	CG15704		FBgn0034103
CG15708	CG15708		FBgn0034099

CG15862	Pka-R2	cAMP-dependent protein kinase R2	FBgn0022382
CG1633	Jafrac1	thioredoxin peroxidase 1	FBgn0040309
CG1657	CG1657		FBgn0030286
CG16711	CG16711		FBgn0036032
CG16738	slp1	sloppy paired 1	FBgn0003430
CG16749	CG16749		FBgn0037678
CG16772	CG16772		FBgn0032835
CG1678	CG1678		FBgn0031176
CG1683	Ant2	Adenine nucleotide translocase 2	FBgn0025111
CG16926	CG16926		FBgn0040732
CG17097	CG17097		FBgn0032275
CG17192	CG17192		FBgn0039472
CG17248	n-syb	n-synaptobrevin	FBgn0013342
CG17320	ScpX	Sterol carrier protein X-related thiolase	FBgn0015808
CG17323	CG17323		FBgn0032713
CG17325	CG17325		FBgn0040993
CG1742	Mgstl	Microsomal glutathione S-transferase-like	FBgn0025814
CG17472	CG17472		FBgn0032868
CG1751	Spase25	Spase 25-subunit	FBgn0030306
CG17525	GstE4	Glutathione S transferase E4	FBgn0063496
CG17571	CG17571		FBgn0259998
CG17597	CG17597		FBgn0032715
CG17633	CG17633		FBgn0032144
CG17637	CG17637		FBgn0037004
CG17666	CG17666		FBgn0036311
CG17673	Acp70A	Accessory gland peptide 70A	FBgn0003034
CG17725	Pepck	Phosphoenolpyruvate carboxykinase	FBgn0003067
CG17751	CG17751		FBgn0038717
CG17752	CG17752		FBgn0038718
CG1787	Hexo2	Hexosaminidase 2	FBgn0041629
CG17924	Acp95EF	Accessory gland-specific peptide 95EF	FBgn0002863
CG1803	regucalcin	regucalcin	FBgn0030362
CG18030	Jon99Fi	Jonah 99Fi	FBgn0039778
CG18064	Met75Cb	Met75Cb	FBgn0028415
CG18170	CG18170		FBgn0035239
CG18180	CG18180		FBgn0036024
CG18211	betaTry	betaTrypsin	FBgn0010357
CG18258	CG18258		FBgn0030827

CG18284	CG18284		FBgn0043825
CG18301	CG18301		FBgn0032265
CG18444	alphaTry	alphaTrypsin	FBgn0003863
CG18594	Pebp1	Phosphatidylethanolamine-binding protein 1	FBgn0038973
CG18681	epsilonTry	epsilonTrypsin	FBgn0010425
CG1951	CG1951		FBgn0039623
CG2034	CG2034		FBgn0015359
CG2229	Jon99Fii	Jonah 99Fii	FBgn0039777
CG2244	MTA1-like	MTA1-like	FBgn0027951
CG2254	CG2254		FBgn0029994
CG2310	CG2310		FBgn0039665
CG2665	PebII	Protein ejaculatory bulb II	FBgn0011694
CG2668	Peb	Protein ejaculatory bulb	FBgn0004181
CG2675	Csat	Csat	FBgn0024994
CG2781	CG2781		FBgn0037534
CG2943	CG2943		FBgn0037530
CG30025	CG30025		FBgn0050025
CG30028	gammaTry	gammaTrypsin	FBgn0010359
CG30031	CG30031		FBgn0050031
CG30042	Cpr49Ab	Cuticular protein 49Ab	FBgn0050042
CG30084	Zasp52	Z band alternatively spliced PDZ-motif protein 52	FBgn0083919
CG30158	CG30158		FBgn0050158
CG30183	CG30183		FBgn0050183
CG3019	su(w[a])	suppressor of white-apricot	FBgn0003638
CG30372	CG30372		FBgn0050372
CG30431	CG30431		FBgn0050431
CG31022	PH4alpha EFB	prolyl-4-hydroxylase-alpha EFB	FBgn0039776
CG31034	Jon99Cii	Jonah 99Cii	FBgn0003356
CG31039	Jon99Ci	Jonah 99Ci	FBgn0003358
CG31072	Lerp	lysosomal enzyme receptor protein	FBgn0051072
CG31094	LpR1	Lipophorin receptor 1	FBgn0066101
CG31233	CG31233		FBgn0051233
CG31362	Jon99Ciii	Jonah 99Ciii	FBgn0003357
CG3143	foxo	forkhead box, sub-group O	FBgn0038197
CG31522	CG31522		FBgn0051522
CG31688	CG31688		FBgn0263355
CG31772	CG31772		FBgn0051772
CG31795	IA-2	IA-2 ortholog	FBgn0031294

CG31872	CG31872		FBgn0051872
CG32031	Argk	Arginine kinase	FBgn0000116
CG32096	rols	rolling pebbles	FBgn0041096
CG32226	CG32226		FBgn0052226
CG32436	CG32436		FBgn0052436
CG3244	Clect27	C-type lectin 27kD	FBgn0031629
CG32473	CG32473		FBgn0052473
CG32479	CG32479		FBgn0052479
CG32548	CG32548		FBgn0052548
CG32694	CG32694		FBgn0052694
CG32703	CG32703		FBgn0052703
CG32815	CG32815		FBgn0052815
CG33110	CG33110		FBgn0053110
CG33113	Rtnl1	Rtnl1	FBgn0053113
CG33173	CG33173		FBgn0053173
CG3329	Prosbeta 2	Proteasome beta2 subunit	FBgn0023174
CG33519	Unc-89	Unc-89	FBgn0053519
CG33555	btsz	bitesize	FBgn0053555
CG33791	CG33791		FBgn0035240
CG33936	CG33936		FBgn0053936
CG3401	betaTub6 0D	beta-Tubulin at 60D	FBgn0003888
CG34120	CG34120		FBgn0083956
CG34205	CG34205		FBgn0085234
CG34330	CG34330		FBgn0085359
CG34381	CG34381		FBgn0085410
CG34383	CG34383		FBgn0085412
CG34394	CG34394		FBgn0085423
CG3481	Adh	Alcohol dehydrogenase	FBgn0000055
CG3484	Adhr	Adh-related	FBgn0000056
CG3581	CG3581		FBgn0038697
CG3612	blw	bellwether	FBgn0011211
CG3725	Ca-P60A	Calcium ATPase at 60A	FBgn0263006
CG3780	Spx	Spliceosomal protein on the X	FBgn0015818
CG3801	Acp76A	Accessory gland-specific peptide 76A	FBgn0015586
CG3861	kdn	knockdown	FBgn0261955
CG3868	CG3868		FBgn0036422
CG3902	CG3902		FBgn0036824
CG3971	Baldspot	Baldspot	FBgn0260960
CG4068	CG4068		FBgn0029738
CG4097	Pros26	Proteasome 26kD subunit	FBgn0002284
CG4105	Cyp4e3	Cytochrome P450-4e3	FBgn0015035

CG4147	Hsc70-3	Heat shock protein cognate 3	FBgn0001218
CG4178	Lsp1beta	Larval serum protein 1 beta	FBgn0002563
CG42268	CG42268		FBgn0259163
CG42339	CG42339		FBgn0259241
CG42349	Pkcdelta	Protein kinase C delta	FBgn0259680
CG42351	CG42351		FBgn0259682
CG42540	CG42540		FBgn0260657
CG42551	larp	La related protein	FBgn0261618
CG42572	MCPH1	Microcephalin	FBgn0260959
CG42610	Fhos		FBgn0261259
CG42666	CG42666		FBgn0261548
CG42669	CG42669		FBgn0261551
CG42683	CG42683		FBgn0261569
CG4272	Axud1		FBgn0261647
CG42732	CG42732		FBgn0261698
CG4286	CG4286		FBgn0034601
CG43119	Ect4	Ect4	FBgn0262579
CG43286	cnc	cap-n-collar	FBgn0262975
CG43442	Nha2	Na[+]/H[+] hydrogen antiporter 2	FBgn0263390
CG43658	CG43658		FBgn0263706
CG4379	Pka-C1	cAMP-dependent protein kinase 1	FBgn0000273
CG4451	Hs6st	Heparan sulfate 6-O-sulfotransferase	FBgn0038755
CG4527	slik	Sterile20-like kinase	FBgn0035001
CG4581	Thiolase	Thiolase	FBgn0025352
CG4600	yip2	yippee interacting protein 2	FBgn0040064
CG4654	Dp	DP transcription factor	FBgn0011763
CG4666	CG4666		FBgn0029838
CG4690	Tsp5D	Tetraspanin 5D	FBgn0029837
CG4692	CG4692		FBgn0035032
CG4734	CG4734		FBgn0033826
CG4750	loopin-1	loopin-1	FBgn0259795
CG4753	CG4753		FBgn0036622
CG4812	Ser8	Ser8	FBgn0019928
CG4898	Tm1	Tropomyosin 1	FBgn0003721
CG4986	Mst57Dc	Male-specific RNA 57Dc	FBgn0011670
CG5016	Mst57Db	Male-specific RNA 57Db	FBgn0011669
CG5021	CG5021		FBgn0035944
CG5155	CG5155		FBgn0031905
CG5162	CG5162		FBgn0030828

CG5177	CG5177		FBgn0031908
CG5326	CG5326		FBgn0038983
CG5389	CG5389		FBgn0036568
CG5594	kcc	kazachoc	FBgn0261794
CG5767	CG5767		FBgn0034292
CG5773	CG5773		FBgn0034290
CG5902	CG5902		FBgn0039136
CG5932	CG5932		FBgn0036996
CG5945	CG5945		FBgn0032494
CG6051	CG6051		FBgn0039492
CG6186	Tsf1	Transferrin 1	FBgn0022355
CG6202	Surf4	Surfeit 4	FBgn0019925
CG6271	CG6271		FBgn0039476
CG6289	Spn77Bc	Serpin 77Bc	FBgn0036970
CG6295	CG6295		FBgn0039471
CG6298	Jon74E	Jonah 74E	FBgn0023197
CG6342	Irp-1B	Iron regulatory protein 1B	FBgn0024957
CG6372	S-Lap1	Sperm-Leucylaminopeptidase 1	FBgn0035915
CG6457	yip7	yippee interacting protein 7	FBgn0040060
CG6467	Jon65Aiv	Jonah 65Aiv	FBgn0250815
CG6483	Jon65Aiii	Jonah 65Aiii	FBgn0035665
CG6492	Ucp4A	Ucp4A	FBgn0030872
CG6543	CG6543		FBgn0033879
CG6580	Jon65Aii	Jonah 65Aii	FBgn0035666
CG6663	Spn77Bb	Serpin 77Bb	FBgn0036969
CG6730	Cyp4d21	Cyp4d21	FBgn0031925
CG6763	CG6763		FBgn0039069
CG6783	fabp	fatty acid bindin protein	FBgn0037913
CG6806	Lsp2	Larval serum protein 2	FBgn0002565
CG7052	TepII	Thiolester containing protein II	FBgn0041182
CG7157	Acp36DE	Accessory gland peptide 36DE	FBgn0011559
CG7176	Idh	Isocitrate dehydrogenase	FBgn0001248
CG7203	CG7203		FBgn0031942
CG7228	pes	peste	FBgn0031969
CG7287	Lcp65Aa	Lcp65Aa	FBgn0020645
CG7349	CG7349		FBgn0030975
CG7478	Act79B	Actin 79B	FBgn0000045
CG7532	l(2)34Fc	lethal (2) 34Fc	FBgn0261534
CG7542	CG7542		FBgn0036738
CG7592	Obp99b	Odorant-binding protein 99b	FBgn0039685

CG7720	CG7720		FBgn0038652
CG7781	CG7781		FBgn0032021
CG7916	CG7916		FBgn0028534
CG7920	CG7920		FBgn0039737
CG7953	CG7953		FBgn0028533
CG8116	CG8116		FBgn0037614
CG8129	CG8129		FBgn0037684
CG8137	Spn2	Serine protease inhibitor 2	FBgn0028987
CG8193	CG8193		FBgn0033367
CG8299	CG8299		FBgn0034052
CG8566	unc-104	unc-104	FBgn0034155
CG8577	PGRP-SC1b	PGRP-SC1b	FBgn0033327
CG8622	Acp53Ea	Accessory gland-specific peptide 53Ea	FBgn0015584
CG8626	Acp53C14a	Acp53C14a	FBgn0034152
CG8628	CG8628		FBgn0250836
CG8654	CG8654		FBgn0034479
CG8661	CG8661		FBgn0030837
CG8722	Nup44A	Nucleoporin 44A	FBgn0033247
CG8786	CG8786		FBgn0036897
CG8867	Jon25Bi	Jonah 25Bi	FBgn0020906
CG8869	Jon25Bii	Jonah 25Bii	FBgn0031654
CG8871	Jon25Biii	Jonah 25Biii	FBgn0031653
CG8952	CG8952		FBgn0030688
CG8982	Acp26Aa	Accessory gland-specific peptide 26Aa	FBgn0002855
CG8994	exu	exuperantia	FBgn0000615
CG8997	CG8997		FBgn0028920
CG9057	Lsd-2	Lipid storage droplet-2	FBgn0030608
CG9074	Mst57Da	Male-specific RNA 57Da	FBgn0011668
CG9090	CG9090		FBgn0034497
CG9092	Gal	beta galactosidase	FBgn0001089
CG9111	LysC	Lysozyme C	FBgn0004426
CG9118	LysD	Lysozyme D	FBgn0004427
CG9130	CG9130		FBgn0035197
CG9132	CG9132		FBgn0030791
CG9133	CG9133		FBgn0035198
CG9159	Kr-h2	Kruppel homolog 2	FBgn0028419
CG9259	CG9259		FBgn0032913
CG9297	CG9297		FBgn0038181
CG9334	Spn3	Serine protease inhibitor 3	FBgn0028986
CG9379	by	blistery	FBgn0000244

CG9380	CG9380		FBgn0035094
CG9396	CG9396		FBgn0037714
CG9399	CG9399		FBgn0037715
CG9494	Tsp29Fa	Tetraspanin 29Fa	FBgn0032074
CG9528	retm	real-time	FBgn0031814
CG9539	Sec61alpha	Sec61alpha	FBgn0086357
CG9540	Ag5r2	Antigen 5-related 2	FBgn0020508
CG9570	CG9570		FBgn0031085
CG9572	CG9572		FBgn0031089
CG9619	CG9619		FBgn0036862
CG9673	CG9673		FBgn0030775
CG9748	bel	belle	FBgn0263231
CG9753	AdoR	Adenosine receptor	FBgn0039747
CG9861	CG9861		FBgn0034844
CG9919	CG9919		FBgn0030742
CR18854	CR18854		FBgn0042174
CR31781	CR31781		FBgn0051781
CG43313	CG43313		FBgn0263005
