# Supplementary material for: Gene expression changes in response to aging compared to heat stress, oxidative stress and ionizing radiation in Drosophila melanogaster
Source: Aging (Albany NY). 2012 Nov 30;4(11):768–89. doi: 10.18632/aging.100499 (PMC3560439; doi:10.18632/aging.100499)
Supplement: Supplementary file 19 [file aging-04-768-s019.docx]

**Supplemental Table S7. Enriched GO terms in gene expression changes caused by sugar transfer**

1. GO enrichment terms for genes up-regulated by sugar transfer

None found

1. GO enrichment terms for genes down-regulated by sugar transfer

| GO:0006508 | proteolysis(49) | 2.86E-06 |
| --- | --- | --- |
| GO:0045297 | post-mating behavior(8) | 6.09E-04 |
| GO:0007320 | insemination(6) | 0.009566 |
| GO:0006629 | lipid metabolic process(24) | 0.047881 |
