# Supplementary material for: Gene expression changes in response to aging compared to heat stress, oxidative stress and ionizing radiation in Drosophila melanogaster
Source: Aging (Albany NY). 2012 Nov 30;4(11):768–89. doi: 10.18632/aging.100499 (PMC3560439; doi:10.18632/aging.100499)
Supplement: Supplementary file 20 [file aging-04-768-s020.docx]

**Supplemental Table S8. Enriched GO terms in genes altered by hydrogen peroxide (sugar effects included)**

1. GO enrichment terms for genes up-regulated in hydrogen peroxide (sugar effects included)

| GO:0050896 | response to stimulus(201) | | | 4.60E-07 | |
| --- | --- | --- | --- | --- | --- |
| GO:0009653 | anatomical structure morphogenesis(136) | | | 1.59E-06 | |
| GO:0007275 | multicellular organismal development(203) | | | 1.63E-05 | |
| GO:0048856 | anatomical structure development(202) | | | 1.60E-04 | |
| GO:0032502 | developmental process(226) | | | 1.68E-04 | |
| GO:0032501 | multicellular organismal process(249) | | | 2.02E-04 | |
| GO:0042221 | response to chemical stimulus(75) | | | 3.08E-04 | |
| GO:0051716 | cellular response to stimulus(134) | | | 8.64E-04 | |
| GO:0048468 | cell development(97) | | | 9.51E-04 | |
| GO:0050794 | regulation of cellular process(205) | | | 9.73E-04 | |
| GO:0065007 | biological regulation(232) | | | 0.00112 | |
| GO:0007165 | signal transduction(110) | | | 0.001661 | |
| GO:0050789 | regulation of biological process(216) | | | 0.002002 | |
| GO:0007154 | cell communication(138) | | | 0.002041 | |
| GO:0003006 | developmental process involved in reproduction(62) | | | 0.003867 | |
| GO:0009791 | post-embryonic development(58) | | | 0.003884 | |
| GO:0002165 | instar larval or pupal development(56) | | | 0.003952 | |
| GO:0051704 | multi-organism process(45) | | | 0.004304 | |
| GO:0009790 | embryo development(60) | | | 0.005251 | |
| GO:0023052 | signaling(134) | | 0.005509 | |  |
| GO:0016265 | death(38) | | 0.007544 | |  |
| GO:0009886 | post-embryonic morphogenesis(49) | | 0.008312 | |  |
| GO:0050793 | regulation of developmental process(51) | | 0.014845 | |  |
| GO:0048610 | cellular process involved in reproduction(66) | | 0.015483 | |  |
| GO:0048477 | oogenesis(54) | | 0.01809 | |  |
| GO:0022414 | reproductive process(88) | | 0.021964 | |  |
| GO:0048707 | instar larval or pupal morphogenesis(47) | | 0.022837 | |  |
| GO:0007559 | histolysis(18) | | 0.02439 | |  |
| GO:0016271 | tissue death(18) | | 0.02439 | |  |
| GO:0007552 | metamorphosis(49) | | 0.024404 | |  |
| GO:0030182 | neuron differentiation(59) | | 0.025197 | |  |
| GO:0051707 | response to other organism(29) | | 0.027074 | |  |
| GO:0007292 | female gamete generation(54) | | 0.030276 | |  |
| GO:0007423 | sensory organ development(49) | | 0.032088 | |  |
| GO:0006950 | response to stress(74) | | 0.032796 | |  |
| GO:0009607 | response to biotic stimulus(29) | | 0.033398 | |  |
| GO:0048731 | system development(158) | 0.038977 | | |  |
| GO:0012501 | programmed cell death(38) | 0.048279 | | |  |
| GO:0022603 | regulation of anatomical structure morphogenesis(30) | 0.048886 | | |  |

1. GO enrichment terms for genes down-regulated in hydrogen peroxide (sugar effects included)

| GO:0055114 | oxidation-reduction process(111) | 2.30E-12 |
| --- | --- | --- |
| GO:0006508 | proteolysis(125) | 3.65E-12 |
| GO:0005975 | carbohydrate metabolic process(73) | 1.11E-07 |
| GO:0044262 | cellular carbohydrate metabolic process(45) | 4.02E-07 |
| GO:0006629 | lipid metabolic process(64) | 1.55E-06 |
| GO:0032787 | monocarboxylic acid metabolic process(24) | 2.58E-04 |
| GO:0019318 | hexose metabolic process(22) | 2.63E-04 |
| GO:0006631 | fatty acid metabolic process(18) | 6.86E-04 |
| GO:0006066 | alcohol metabolic process(33) | 0.004615 |
| GO:0008152 | metabolic process(528) | 0.005934 |
| GO:0005996 | monosaccharide metabolic process(22) | 0.00787 |
| GO:0044281 | small molecule metabolic process(105) | 0.008164 |
| GO:0006082 | organic acid metabolic process(46) | 0.012088 |
| GO:0019752 | carboxylic acid metabolic process(46) | 0.012088 |
| GO:0043436 | oxoacid metabolic process(46) | 0.012088 |
| GO:0006091 | generation of precursor metabolites and energy(32) | 0.037811 |
