# Supplementary material for: Gene expression changes in response to aging compared to heat stress, oxidative stress and ionizing radiation in Drosophila melanogaster
Source: Aging (Albany NY). 2012 Nov 30;4(11):768–89. doi: 10.18632/aging.100499 (PMC3560439; doi:10.18632/aging.100499)
Supplement: Supplementary file 21 [file aging-04-768-s021.docx]

**Supplemental Table S9. Enriched GO terms in genes altered by ionizing radiation (sugar effects included)**

1. GO enrichment terms for genes up-regulated in Ionizing radiation (sugar effects included)

| GO:0006950 | response to stress(94) | 2.58E-11 | | | |
| --- | --- | --- | --- | --- | --- |
| GO:0033554 | cellular response to stress(55) | 5.56E-07 | |  |  |
| GO:0009069 | serine family amino acid metabolic process(10) | 5.11E-05 | |  |  |
| GO:0006520 | cellular amino acid metabolic process(29) | 4.98E-04 | |  |  |
| GO:0009056 | catabolic process(61) | 6.93E-04 | |  |  |
| GO:0044106 | cellular amine metabolic process(30) | 0.001951 | |  |  |
| GO:0006974 | response to DNA damage stimulus(35) | 0.002467 | |  |  |
| GO:0006563 | L-serine metabolic process(5) | 0.003036 | |  |  |
| GO:0008652 | cellular amino acid biosynthetic process(12) | 0.009553 |  |  |  |
| GO:0044281 | small molecule metabolic process(74) | 0.009616 |  |  |  |
| GO:0006082 | organic acid metabolic process(34) | 0.013549 |  |  |  |
| GO:0019752 | carboxylic acid metabolic process(34) | 0.013549 |  |  |  |
| GO:0043436 | oxoacid metabolic process(34) | 0.013549 |  |  |  |
| GO:0044271 | cellular nitrogen compound biosynthetic process(29) | 0.019953 | | |  |
| GO:0030163 | protein catabolic process(22) | 0.048772 | | |  |

1. GO enrichment terms for genes down-regulated in Ionizing radiation (sugar effects included)

| GO:0055114 | oxidation-reduction process(84) | 1.09E-09 |
| --- | --- | --- |
| GO:0006508 | proteolysis(87) | 1.22E-06 |
| GO:0006091 | generation of precursor metabolites and energy(31) | 8.54E-05 |
| GO:0045297 | post-mating behavior(11) | 2.54E-04 |
| GO:0006629 | lipid metabolic process(45) | 0.002102 |
| GO:0015980 | energy derivation by oxidation of organic compounds(24) | 0.003842 |
| GO:0051704 | multi-organism process(45) | 0.013874 |
| GO:0044281 | small molecule metabolic process(79) | 0.038327 |
| GO:0046034 | ATP metabolic process(12) | 0.0446 |
