# Supplementary material for: Gene expression changes in response to aging compared to heat stress, oxidative stress and ionizing radiation in Drosophila melanogaster
Source: Aging (Albany NY). 2012 Nov 30;4(11):768–89. doi: 10.18632/aging.100499 (PMC3560439; doi:10.18632/aging.100499)
Supplement: Supplementary file 22 [file aging-04-768-s022.docx]

**Supplemental Table S10. Enriched GO terms in genes altered by hydrogen peroxide (sugar effects excluded)**

1. GO enrichment terms for genes up-regulated in hydrogen peroxide (sugar effects excluded)

| GO:0009653 | anatomical structure morphogenesis(130) | 1.37E-06 | |
| --- | --- | --- | --- |
| GO:0050896 | response to stimulus(189) | 1.95E-06 | |
| GO:0007275 | multicellular organismal development(196) | 2.73E-06 | |
| GO:0048856 | anatomical structure development(196) | 1.62E-05 | |
| GO:0032502 | developmental process(217) | 4.49E-05 | |
| GO:0032501 | multicellular organismal process(239) | 4.65E-05 | |
| GO:0042221 | response to chemical stimulus(71) | 5.71E-04 | |
| GO:0051716 | cellular response to stimulus(128) | 6.86E-04 | |
| GO:0048468 | cell development(93) | 7.21E-04 | |
| GO:0050794 | regulation of cellular process(195) | 8.13E-04 | |
| GO:0065007 | biological regulation(219) | 0.00192 | |
| GO:0050789 | regulation of biological process(205) | 0.001983 | |
| GO:0003006 | developmental process involved in reproduction(60) | | 0.002305 |
| GO:0007165 | signal transduction(104) | 0.002851 | |
| GO:0009790 | embryo development(58) | 0.003363 | |
| GO:0007154 | cell communication(130) | 0.004316 | |
| GO:0051704 | multi-organism process(43) | 0.005179 | |
| GO:0048610 | cellular process involved in reproduction(64) | 0.007999 | |
| GO:0022414 | reproductive process(85) | 0.011218 | |
| GO:0023052 | signaling(126) | 0.012452 | |
| GO:0050793 | regulation of developmental process(49) | 0.013228 | |
| GO:0048477 | oogenesis(52) | 0.014271 | |
| GO:0048731 | system development(152) | 0.015364 | |
| GO:0007292 | female gamete generation(52) | 0.023701 | |
| GO:0051707 | response to other organism(28) | 0.024779 | |
| GO:0009791 | post-embryonic development(53) | 0.029291 | |
| GO:0009607 | response to biotic stimulus(28) | 0.030431 | |
| GO:0016265 | death(35) | 0.033171 | |
| GO:0002165 | instar larval or pupal development(51) | 0.033329 | |

b) GO enrichment terms for genes that down-regulated in hydrogen peroxide (sugar effects excluded)

| GO:0055114 | oxidation-reduction process(113) | 2.63E-14 |
| --- | --- | --- |
| GO:0005975 | carbohydrate metabolic process(66) | 4.59E-08 |
| GO:0044262 | cellular carbohydrate metabolic process(42) | 6.85E-08 |
| GO:0019318 | hexose metabolic process(22) | 1.04E-05 |
| GO:0044281 | small molecule metabolic process(100) | 2.69E-05 |
| GO:0006631 | fatty acid metabolic process(18) | 4.50E-05 |
| GO:0006066 | alcohol metabolic process(33) | 7.27E-05 |
| GO:0006629 | lipid metabolic process(53) | 1.09E-04 |
| GO:0032787 | monocarboxylic acid metabolic process(22) | 2.30E-04 |
| GO:0005996 | monosaccharide metabolic process(22) | 3.76E-04 |
| GO:0006082 | organic acid metabolic process(44) | 5.63E-04 |
| GO:0019752 | carboxylic acid metabolic process(44) | 5.63E-04 |
| GO:0043436 | oxoacid metabolic process(44) | 5.63E-04 |
| GO:0042180 | cellular ketone metabolic process(45) | 0.00305 |
| GO:0006006 | glucose metabolic process(14) | 0.015429 |
| GO:0044255 | cellular lipid metabolic process(35) | 0.01572 |
| GO:0044275 | cellular carbohydrate catabolic process(13) | 0.020119 |
| GO:0046164 | alcohol catabolic process(13) | 0.020119 |
| GO:0006508 | proteolysis(83) | 0.024911 |
| GO:0006091 | generation of precursor metabolites and energy(29) | 0.024939 |
| GO:0044282 | small molecule catabolic process(19) | 0.038591 |
