# Supplementary material for: Gene expression changes in response to aging compared to heat stress, oxidative stress and ionizing radiation in Drosophila melanogaster
Source: Aging (Albany NY). 2012 Nov 30;4(11):768–89. doi: 10.18632/aging.100499 (PMC3560439; doi:10.18632/aging.100499)
Supplement: Supplementary file 23 [file aging-04-768-s023.docx]

**Supplemental Table S11. Enriched GO terms in genes altered by ionizing radiation (sugar effects excluded)**

a) GO enrichment terms for genes up-regulated in Ionizing radiation (sugar effects excluded)

| GO:0006950 | response to stress(87) | 1.72E-09 |
| --- | --- | --- |
| GO:0033554 | cellular response to stress(52) | 2.55E-06 |
| GO:0009069 | serine family amino acid metabolic process(10) | 3.20E-05 |
| GO:0006520 | cellular amino acid metabolic process(28) | 6.49E-04 |
| GO:0009056 | catabolic process(58) | 0.001558 |
| GO:0044106 | cellular amine metabolic process(29) | 0.00232 |
| GO:0006563 | L-serine metabolic process(5) | 0.002383 |
| GO:0008652 | cellular amino acid biosynthetic process(12) | 0.005736 |
| GO:0006082 | organic acid metabolic process(33) | 0.01293 |
| GO:0019752 | carboxylic acid metabolic process(33) | 0.01293 |
| GO:0043436 | oxoacid metabolic process(33) | 0.01293 |
| GO:0006974 | response to DNA damage stimulus(32) | 0.019493 |
| GO:0030163 | protein catabolic process(22) | 0.022491 |
| GO:0044281 | small molecule metabolic process(70) | 0.024083 |
| GO:0006564 | L-serine biosynthetic process(4) | 0.043807 |
| GO:0035079 | polytene chromosome puffing(5) | 0.045541 |
| GO:0035080 | heat shock-mediated polytene chromosome puffing(5) | 0.045541 |

b) GO enrichment terms for genes down-regulated in Ionizing radiation (sugar effects excluded)

| GO:0055114 | oxidation-reduction process(79) | 1.18E-11 |
| --- | --- | --- |
| GO:0006091 | generation of precursor metabolites and energy(29) | 2.44E-05 |
| GO:0044281 | small molecule metabolic process(78) | 5.33E-05 |
| GO:0015980 | energy derivation by oxidation of organic compounds(22) | 0.002787 |
| GO:0006082 | organic acid metabolic process(32) | 0.032492 |
| GO:0019752 | carboxylic acid metabolic process(32) | 0.032492 |
| GO:0043436 | oxoacid metabolic process(32) | 0.032492 |
| GO:0009407 | toxin catabolic process(4) | 0.043117 |
| GO:0042178 | xenobiotic catabolic process(4) | 0.043117 |
| GO:0046701 | insecticide catabolic process(4) | 0.043117 |
